# Mapping the global distribution of spotted fever group rickettsiae: a systematic review with modelling analysis

**DOI:** 10.1016/S2589-7500(22)00212-6

**Published:** 2022-11-21

**Authors:** Yuan-Yuan Zhang, Yan-Qun Sun, Jin-Jin Chen, Ai-Ying Teng, Tao Wang, Hao Li, Simon I Hay, Li-Qun Fang, Yang Yang, Wei Liu

**Affiliations:** State Key Laboratory of Pathogen and Biosecurity, Beijing Institute of Microbiology and Epidemiology, Beijing, China (Y-Y Zhang BSc, Y-Q Sun MPH, J-J Chen MSc, A-Y Teng MSc, T Wang BSc, Prof H Li PhD, Prof L-Q Fang PhD, Prof W Liu PhD); Department of Health Metrics Sciences, School of Medicine (Prof S I Hay DSc), and Institute for Health Metrics and Evaluation (Prof S I Hay), University of Washington, Seattle, WA, USA; Shanghai Institute of Infectious Disease and Biosecurity, School of Public Health, Fudan University, Shanghai, China (Prof L-Q Fang); Department of Statistics, Franklin College of Arts and Sciences, University of Georgia, Athens, GA, USA (Prof Y Yang PhD); Department of Laboratorial Science and Technology, School of Public Health, Peking University, Beijing, China (Prof W Liu); Center for Global Health, School of Public Health, Nanjing Medical University, Nanjing, China (Prof W Liu)

## Abstract

**Background:**

Emerging and re-emerging spotted fever group (SFG) rickettsioses are increasingly recognised worldwide as threats to public health, yet their global distribution and associated risk burden remain poorly understood.

**Methods:**

In this systematic review and modelling analysis, we mapped global distributions of all confirmed species of SFG rickettsiae (SFGR) detected in animals, vectors, and human beings, using data collected from the literature. We assessed ecological drivers for the distributions of 17 major SFGR species using machine learning algorithms, and mapped model-predicted risks.

**Findings:**

Between Jan 1, 1906, and March 31, 2021, we found reports of 48 confirmed SFGR species, with 66 133 human infections worldwide, with a large spatial variation across the continents. 198 vector species were detected to carry 47 of these *Rickettsia* spp. (146 ticks, 24 fleas, 15 mosquitoes, six mites, four lice, two keds, and one bug). Based on model-predicted global distributions of the 17 major SFGR species, we found five spatial clusters aggregated by ecological similarity in terms of environmental and ecoclimatic features. *Rickettsia felis* is the leading SFGR species to which 4.4 billion (95% CI 3.8–5.3 billion) people are at risk, followed by *Rickettsia conorii* (3.7 billion) and *Rickettsia africae* (3.6 billion).

**Interpretation:**

The wide spectrum of vectors is contributing substantially to the increasing incidence of SFGR infections among humans. Awareness, diagnosis, and surveillance of SFGR infections should be improved in the high-risk regions, especially in areas where human infections are underreported.

**Funding:**

National Key Research and Development Program of China.

## Editorial note

The Lancet Group takes a neutral position with respect to territorial claims in published maps and institutional affiliations.

## Introduction

Spotted fever group rickettsiae (SFGR) are a group of obligate intracellular bacteria belonging to the genus *Rickettsia* within the family Rickettsiaceae in the order Rickettsiales. SFGR are found worldwide, infecting a wide array of wild and domestic vertebrates mainly through tick bites. Other hematophagous arthropods that serve as vectors include lice, mites, mosquitoes, and fleas.^1,2^ SFGR are prevalent in nature, but human cases have mostly been reported in the USA and Europe and surveillance of human infection is inadequate.^3^

The biological variety and geographical scope of recognised tick-associated rickettsiae have dramatically increased since the 1980s, probably driven by advances in molecular diagnostic techniques,^4^ including identification of novel rickettsiae species by high-throughput sequencing.^5^ In addition to local surveillance studies on SFGR pathogens and associated human disease burdens, systematic reviews and meta-analyses have also been done to piece together data at larger scale, up to that of continents.^1,6,7^ However, a systematic study of the spatial distribution, ecological niches, and clinical manifestations of SFGR at the global scale has not been done. Such a study is needed for development of guidelines for diagnosis, surveillance, and control of SFGR.

We present a comprehensive review on the global distribution of SFGR. We used a machine-learning approach to study the ecological niche of important SFGR species and contributions of environmental, ecoclimatic, and biological variables at the global scale with a resolution of 10 km × 10 km pixel.^8,9^ Using these ecological models, we mapped the risk of potential SFGR occurrences at locations with little or no epidemiological investigation^10^ to guide future field investigation and surveillance of SFG rickettsioses.

## Methods

### Literature search and data preparation

We searched PubMed, Web of Science, medRvix, and bioRvix for articles published between Jan 1, 1906, and March 31, 2021, using the search terms “Spotted fever group *rickettsia*” or “spotted fever” or “SFG *rickettsia*”, without any language restrictions. The search results were first screened for title or abstract, and then relevant papers underwent full-text screening. We also searched the GenBank database with the same search terms to identify any SFGR species that have been detected and sequenced. Studies were eligible if they described laboratory detections of SFGR in arthropod vectors, animals, or humans, which resulted from natural infections rather than laboratory challenges. We excluded studies that met any of the following criteria: data without laboratory-confirmed detection or definite species identifications of SFGR, drug or vaccine trials without geographical or clinical information of cases, studies focusing on molecular or cellular structures and functions or transstadial transmission of pathogens in laboratory settings but providing no information on the origin and location of those pathogens, and full texts unavailable or their key references cannot be found. More detailed inclusion and exclusion criteria are given in appendix 1 (p 7). All articles were screened by two independent reviewers (Y-QS and TW). The following types of infection events of SFGR were assembled: vectors with molecular assay or pathogen isolation evidence, animals (livestock and wildlife) with molecular evidence, confirmed human cases confirmed with molecular evidence or serological assay,^11^ and humans with serological evidence. Full details of qualified detection methods of these infection events are given in appendix 1 (p 8) and detailed data extraction, geopositioning of the occurrence data, and assembling occurrence data of SFGR species and covariates are included in appendix 1 (p 3). The details of the analysis on clinical spectrum of rickettsioses are provided in appendix 1 (p 4).

### Ecological modelling risk for SFGR occurrence

To explore the relationship between the probability of SFGR occurrences and environmental, ecoclimatic, and biological drivers that were known or hypothesised to contribute to the ecological suitability of SFGR,^6,12^ we compared the predictive performance of three ecological modelling approaches including Boosted Regression Trees (BRT), Random Forest model, and Least Absolute Shrinkage and Selection Operator logistic regression. Full details about the original spatial resolutions, time spans, and sources of the datasets on the 40 extracted ecological variables are provided in appendix 1 (pp 15–16). We calculated the mean of these variables over their corresponding time spans to be used as predictors for ecological modelling.^13^ We calculated the mean of data provided with a finer resolution than the study grid (10 × 10 km) to match the desired resolution. For BRT modelling, pseudo-absence locations were sampled randomly within a range of 30–3000 km around the occurrence locations with a 3:1 ratio.^14–15^ For each occurrence location, the range of sampling was determined by the shortest distance to other occurrence locations. We sampled 80% training set and 20% test set via random splitting and fitted a model, which was repeated 100 times.^13,16–17^ We obtained 100 models based on 100 resampled training datasets for each target species, to which we refer as a model assembly. The relative contributions of all predictors and the area-under-curve (AUC) of receiver operating characteristic (ROC) curves for test sets were averaged over the 100 models in the assembly to represent the final estimation results and predictive performance of the model assembly. The best threshold value used for final predictions of presence or absence of a given SFGR species at the global scale was based on the Youden index derived from the average ROC over the 100 models in the assembly.^17,18^ The detailed modelling processes are shown in appendix 1 (pp 4–5).

### Clustering SFGR with similar ecological niches and their spatial distribution

To explore similarity in ecological niches among the 17 predominant SFGR species, we did a hierarchical cluster analysis based on the Ward’s minimum variance method.^19^ Firstly, we selected predictors that were influential in the final model assembly (average relative contribution ≥3%) for at least one of 17 *Rickettsia* species. For each species, the following three quantities associated with each ecological predictor were calculated as features for clustering. The first quantity is the average relative contribution of this predictor in the model assembly, which is set to zero if this predictor was not used in the final model assembly for this *Rickettsia* sp. The second quantity is a measure for the difference in this predictor between case grids (containing an occurrence of the given *Rickettsia* sp.) and all grids. Specifically, we first calculated the median value of this predictor among all case grids and quartile intervals of the predictor among all grids in the world. We then assigned the numbers 1–4 according to which quartile interval the median lies in, eg, assign 1 (4) if the median lies in the lowest (highest) quartile. The third quantity is the linear correlation between the predictor and model-predicted presence probabilities of the given *Rickettsia* sp. among all grids (averaged over the 100 models in the assembly). These three quantities of all ecological predictors jointly serve as features for clustering. We created a dendrogram to show the clustering pattern of these 17 rickettsia species, together with a thematic matrix illustrating the features. We mapped geographical distributions of the identified clusters of SFGR by defining the presence of each cluster as the presence of any *Rickettisa* spp. in that cluster.

### Role of the funding source

The funder of the study had no role in the design and conduct of the study; collection, management, analysis, and interpretation of the data; preparation, review, or approval of the manuscript; and decision to submit the manuscript for publication.

## Results

**Figure 1:**
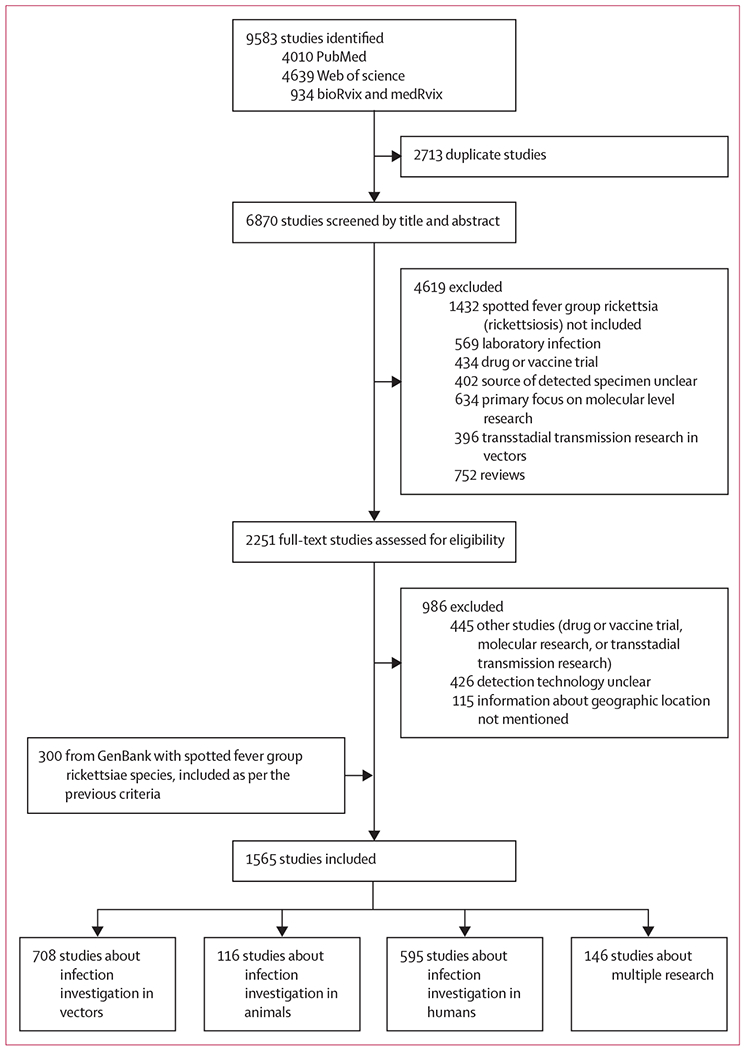
Trial profile

**Figure 2:**
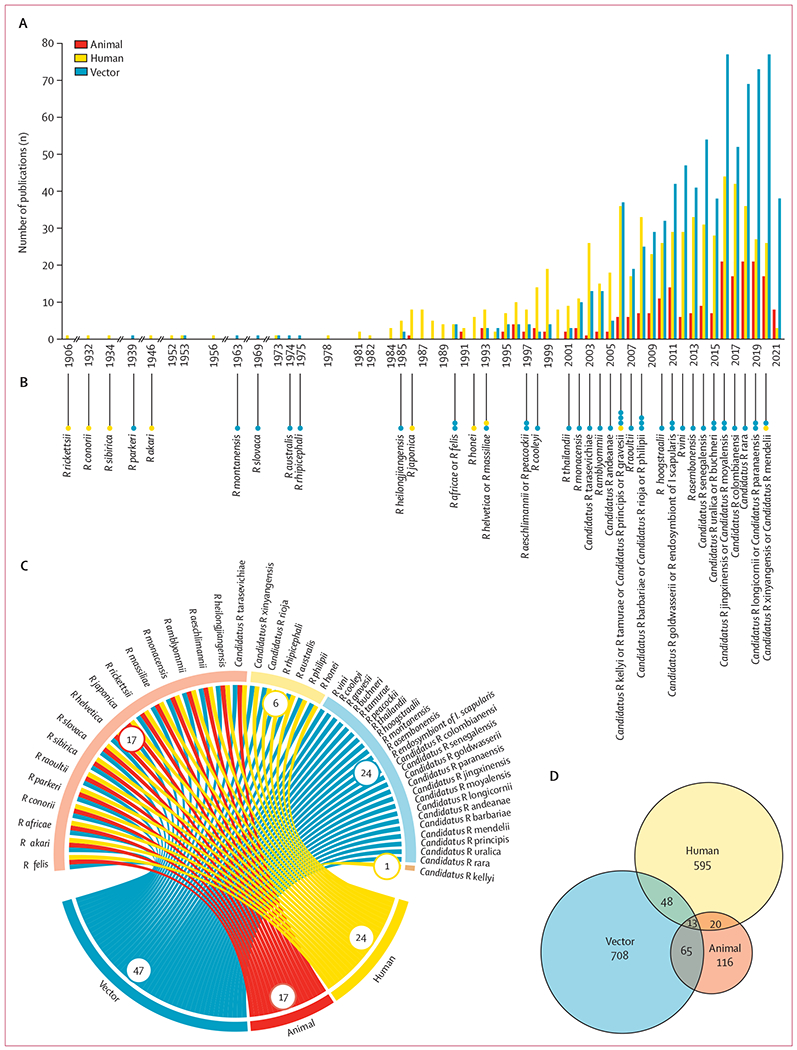
Publications about SFG rickettsiae in animals, vectors, and human beings (January, 1906, to March, 2021) (A) Annual number of publications on SFGR stratified by host type. (B) Year of first reporting for each SFGR species. Dots indicate source of first detection. (C) Chord diagram between SFGR species and host types. (D) Overall number of publications on SFGR detection in vectors, animals, and humans. FGR=spotted fever group rickettsiae.

We retrieved a total of 6870 unique studies, among which 5605 (81·6%) were excluded according to the exclusion criteria (figure 1). The remaining 1265 (18·4%) articles, together with 300 records obtained from GenBank, constitute the final dataset for our study (appendix 1 pp 17–18, appendix 2 and appendix 3). Of the 1565 publications included in this study, 708 (45·2%) included rickettsia detections only in vectors, 595 (38·0%) included infections only in humans, and 116 (7·4%) included infections only in animals (figure 2D). The remaining 146 publications included SFGR detections in at least two types of host, mainly involving vectors and animals (65 [44·5%] of 146) or in vectors and humans (48 [32·9%]). The number of publications each year on SFGR has increased since the 1980s, particularly for the detection in vectors, although with a notable setback in 2021, probably due to the COVID-19 pandemic (figure 2A). In total, 48 SFGR species (including 17 Candidatus Rickettsiae spp.) were reported, the earliest of which was *Rickettsia rickettsii* (reported in a single patient in 1906^20^) and most were detected in or after the second half of the 20th century. 39 (81%) of 48 *Candidatus* Rickettsiae spp. were first detected in arthropod vectors, and the rest (nine [19%]) were first detected in human beings (figure 2B). All but one reported *Rickettsia* spp. (*Candidatus* R kellyi) were detected in vectors, 24 were detected in humans, and 17 were detected in animals. 24 were detected only in vectors, and *Candidatus* R kellyi was the only one solely detected in humans. No *Rickettsia* sp. was solely detected in animals. Six *Rickettsia* spp. were detected in both vectors and humans but not in animals, and 17 were found inhabiting all three types of host (figure 2C). All *Rickettsia* spp. detected in both vectors and animals were also detected in humans.

**Figure 3:**
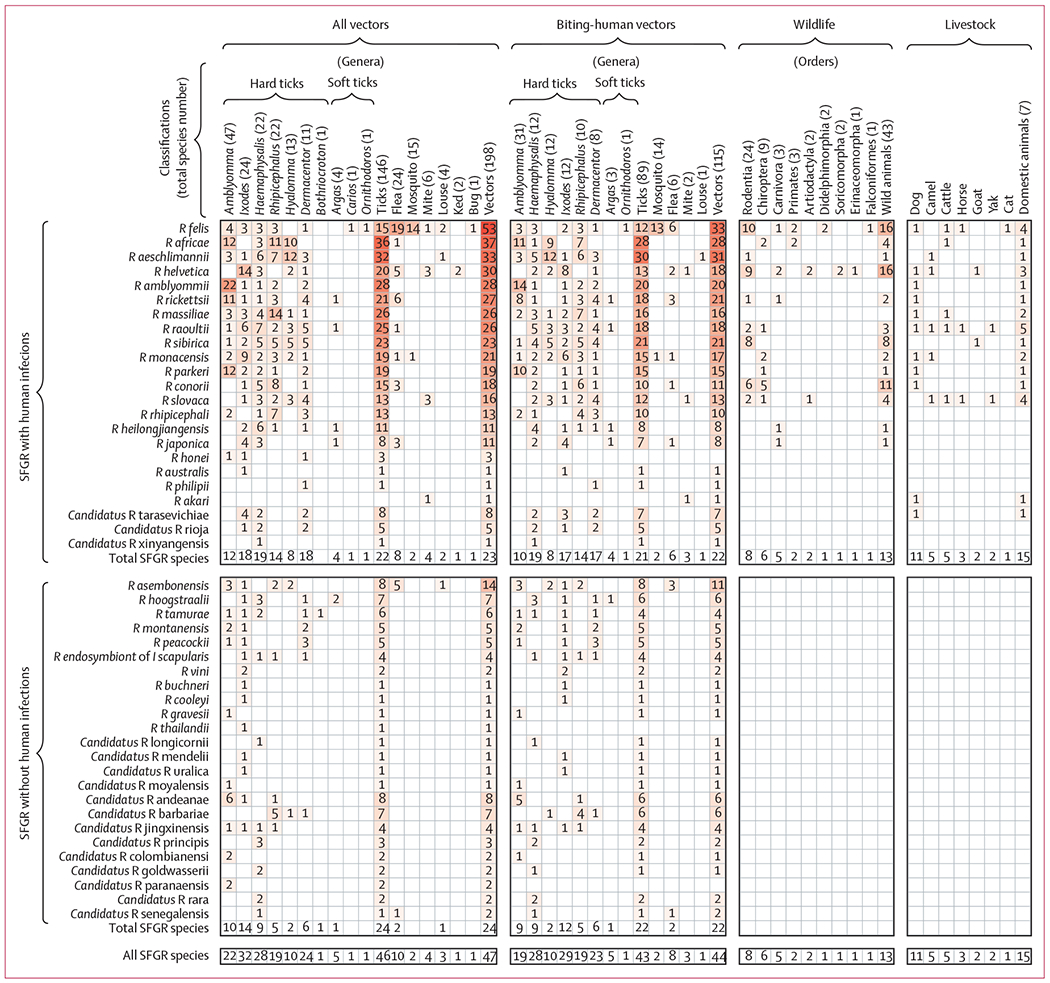
Numbers of vector and animal species from which each SFGR species was detected Vectors include ticks, fleas, mosquitoes, mites, louses, keds, and bugs. Ticks are further classified by genus. Animals include both wildlife and livestock, and wild animals are classified into orders. The total number of distinct host species harbouring SFGR in each host category is shown in the column header. The total number of rickettsia species detected in each host category is shown within the matrix. SFGR=spotted fever group rickettsiae.

Based on 834 publications involving SFGR detection in vectors, a total of 198 vector species were reported to carry 47 SFGR, composed primarily of ticks (146 species), followed by fleas (24 species), mosquitoes (15 species), mites (six species), lice (four species), keds (two species), and one bug (figure 3). Among all the identified tick-borne SFGR, *Rickettsia africae* was found to infect the greatest number of tick species (36 species), followed by *Rickettsia aeschlimannii* (32), *Rickettsia amblyommii* (28), *Rickettsia massiliae* (26), *Rickettsia raoultii* (25), *Rickettsia sibirica* (23), *R rickettsii* (21), and *Rickettsia helvetica* (20). *Rickettsia felis*, a primarily flea-borne *Rickettsia*, was found to infect the most vector species (53 species including 19 flea species), followed by *R africae* (37), *R aeschlimannii* (33), and *R helvetica* (30). Full details about vectors and SFGR species they carry and SFGR species found in two or more vector types are given in appendix 1 (pp 6, 37–38). Among the 214 publications involving SFGR detection in animals, 43 species of wild animals (in nine orders) and seven domestic animals were reported to have SFGR infections (figure 3). Order Rodentia had the most SFGR-carrying species and carried the greatest number of SFGR (24 wildlife species carried eight *Rickettsia* spp.), followed by Chiroptera (nine carried six), and Carnivora (three carried five). Details about animals and SFGR species they carry are given in appendix 1 (pp 6, 39). Based on 676 publications involving SFGR detection in humans, a total of 66 133 human cases with SFGR infections were reported across the world, of which 19 734 cases were confirmed by molecular assays and 46 399 by serological tests (appendix 1 p 20). The most predominant species among the 24 species associated with human infections were *R rickettsii* (42·2%) and *R conorii* (33·0%), followed by *R sibirica* (8·4%), *R felis* (3·2%), and *R japonica* (3·1%; appendix 1 p 20). Details about clinical spectrum of rickettsioses are given in appendix 1 (pp 6, 21–22).

**Figure 4:**
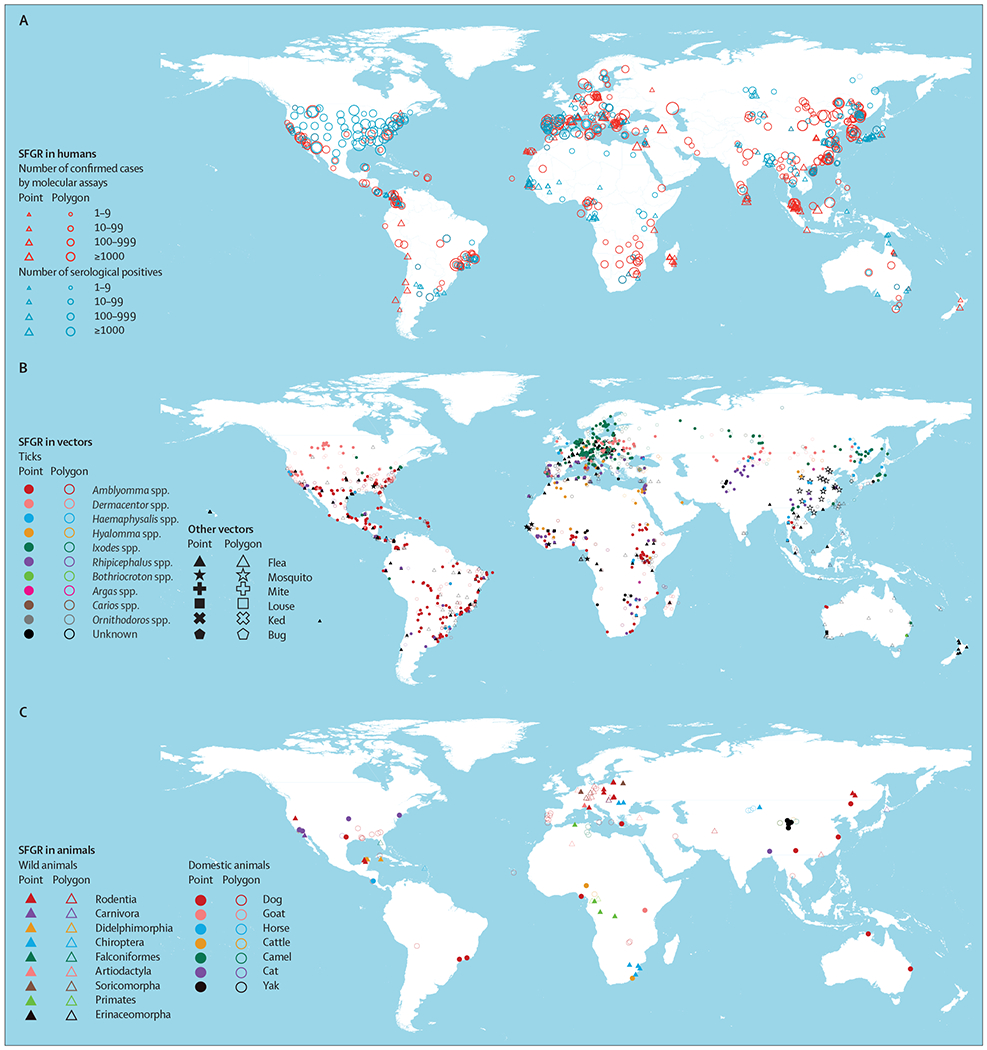
Global distributions of SFGR detection events (A) Humans. (B) Vectors. (C) Animals. SFGR=spotted fever group rickettsiae.

Most confirmed SFGR rickettsioses were distributed across North America, the Mediterranean region, and east Asia. Human infections detected by serological surveys were distributed over central America, southern Africa, southeast Asia, and east Asia (figure 4A). The spatial clustering pattern of SFGR detected in vectors varied by latitude or continent (figure 4B). SFGR detections in *Amblyomma* were mainly recorded in the Americas and Africa, especially in coastal regions, whereas detections in *Dermacentor* and *Ixodes* were mainly distributed in areas at high latitudes (approximately 30°N–60°N). Although SFGR were detected in *Rhipicephalus* worldwide, those in *Haemaphysalis* and *Hyalomma* were mainly found in Eurasia, with a few associated with *Hyalomma* distributed along the rim of the Sahara Desert. In general, SFGR detections in ticks were more widely distributed than those in other arthropod vectors. SFGR infections in wildlife were more frequently reported in Europe and Africa, whereas those infections in livestock were reported worldwide, with a higher frequency in dogs and cats in North America than in other domestic animals (figure 4C).

**Figure 5:**
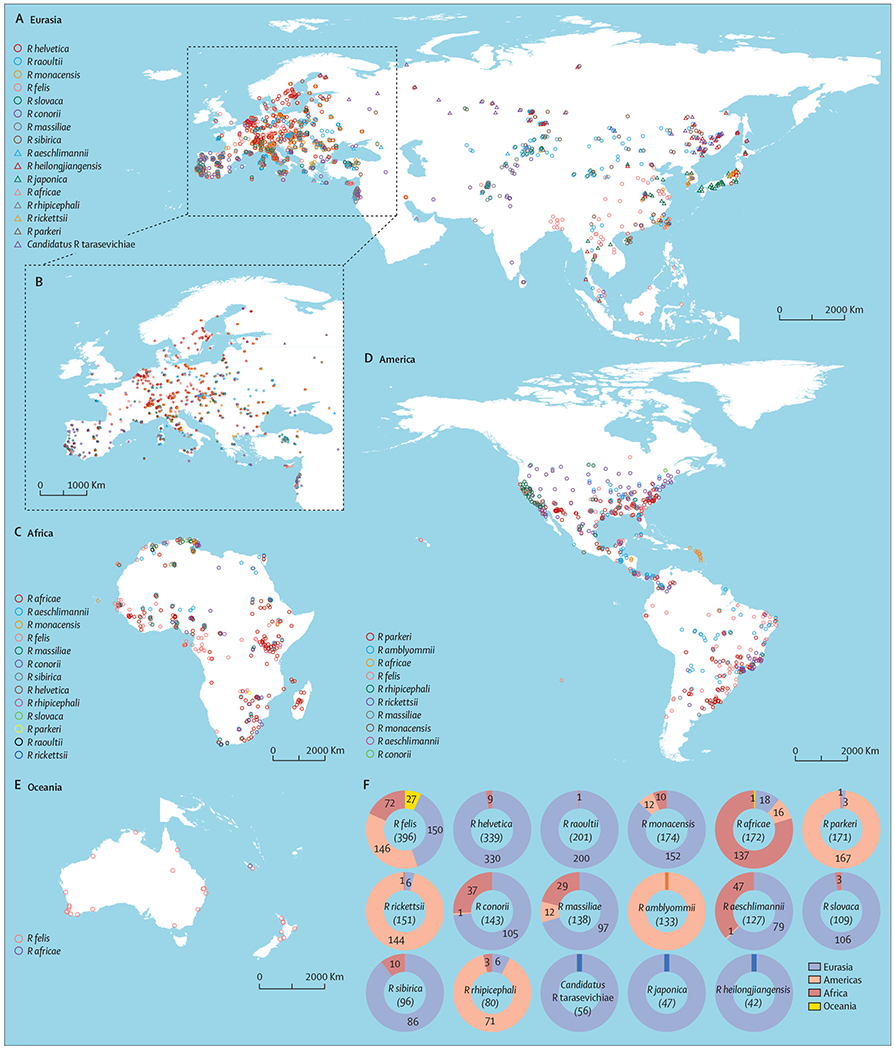
Global distributions of 17 predominant SFGR species in four continents (A) Eurasia. (B) Zoomed-in area of Europe. (C) Africa. (D) Americas. (E) Oceania. (F) Number of detection locations (after deduplication) in four continents for each predominant SFGR species. SFGR=spotted fever group rickettsiae.

The abundance of the predominant 17 SFGR species varies substantially across the four described continents. The greatest diversity of SFGR species was seen in Eurasia, in which 16 SFGR species were recorded (figure 5A; appendix 1 p 40), followed by 13 species in Africa (figure 5C), ten species in the Americas (figure 5D), and two species in Oceania (figure 5E). The continental distribution also differed among the SFGR species. *R felis* was the most widely recorded, covering all four inhabitable continents, with more observations along coastal areas. *R helvetica*, *R raoultii*, *R monacensis*, *R conorii*, *R massiliae*, *R aeschlimannii*, *R slovaca*, *R sibirica*, *Candidatus* R tarasevichiae, *R japonica*, and *R heilongjiangensis* were mainly distributed in Eurasia. *R parkeri*, *R rickettsii*, *R amblyommii,* and *R rhipicephali* were mainly found in the Americas. *R africae* was mainly observed in the coastal countries of Africa (figure 5F). By contrast, the distributions of the remaining 31 non-predominant rickettsiae were more locally focused (appendix 1 p 41).

Based on the average test AUC over 100 models in the model assembly for each algorithm, BRT and Random Forest outperformed Least Absolute Shrinkage and Selection Operator in prediction, and for most SFGR species BRT outperformed Random Forest (11 of 17; appendix 1 pp 42–44). Therefore, we selected BRT to do the final analysis and to map the global distribution of SFGR. The ecological models showed accurate predictions for all predominant SFGR species, with the average AUC of the testing ROC curves ranging from 0·936 for *R felis* to 0·984 for *R helvetica* (table). The model-estimated drivers and their relative contributions varied by species, but the most influential predictors were climatic drivers. And some animal-related factors, including sheep density, horse density, and mammalian richness, contributed substantially as well, with relative contribution more than 10% for eight SFGR species (*R africae*, *R heilongjiangensis*, *R massiliae*, *R raoultii*, *R rhipicephali*, *R rickettsii*, *R sibirica*, and *R slovaca*). The coverage of cropland was the most important driver for the distribution of *R massiliae*. Details about the drivers and their relative contributions to the predominant 17 SFGR are given in appendix 1 (pp 6, 23–24, 45–61). By overlaying population counts on the maps of SFGR-suitable areas, we assessed the potential impact of major SFGR species in terms of both at-risk population size and geographical range. *R felis* was predicted to affect the most people (4·4 billion, 95% CI 3·8–5·3 billion) and have the widest distribution (15·2 million km^2^, 12·0–20·0 million), followed by *R conorii* (3·7 billion people, 2·8–4·5 billion; and 11·21 million km^2^, 7·8–15·0 million), and *R africae* (3·6 billion people, 2·6–4·4 billion; and 9·9 million km^2^, 6·3–14·5 million; table). In general, the model-predicted areas with medium to high risks of SFGR infection are more extensive than the reported locations. Global maps overlaying recorded and predicted distributions of SFGR are shown in appendix 1 (pp 62–84). We compared the results of two machine learning models (BRT and Random Forest) and found that relative contributions of the top five factors for the two models are nearly identical and the ranks are similar (appendix 1 pp 25–26).

Based on the ecological similarity represented by environmental and ecoclimatic predictors, the 17 SFGR species were grouped into five clusters with clear patterns of spatial aggregation (appendix 1 p 85). *R helvetica* and *Candidatus* R tarasevichiae constitute Cluster I, which covers regions at high latitudes (30°N–60°N) that feature low temperature and high coverages of cropland (appendix 1 pp 68, 78, 85). *R massiliae*, *R conorii*, and *R aeschlimannii* were grouped into Cluster II, which was mainly found in South America, sub-Saharan Africa, the Mediterranean region, central Asia, east Asia, and South Australia; and features high temperature and precipitation, high coverages of cropland, and low elevations (appendix 1 pp 62, 65, 70, 85). Cluster III, composed of *R japonica*, *R heilongjiangensis*, and *R africae*, shares similar distributions with Cluster II but with additional risk areas in the east of North America. This cluster stretches over biogeographical areas characterised by high annual precipitation, high percentage of cropland, and high mammal richness (appendix 1 pp 63, 67, 69, 85). *R monacensis*, *R felis*, *R sibirica*, *R raoultii*, and *R slovaca* were grouped into Cluster IV, which is distributed in the same regions as Cluster III but with a wider scope in which the weather is warm and humid and vegetation and animals are abundant (appendix 1 pp 66, 71, 73, 76–77, 85). Cluster V comprises of *R rickettsii*, *R amblyommii*, *R parkeri*, and *R rhipicephali*, which are mainly distributed in the Americas, sub-Saharan Africa, and southeast Asia, and feature more grassland than cropland (appendix 1 pp 64, 72, 74–75, 85). Finally, we combined the species of each cluster and fitted BRT models to assess ecological drivers for each cluster. For Cluster I, the annual mean temperature contributed the most. The most influential contributors were precipitation of warmest quarter for Cluster II, annual precipitation for Cluster III, coverage of cropland for Cluster IV, and precipitation of coldest quarter for Cluster V (appendix 1 p 27).

## Discussion

This study is, to our knowledge, the first systematic review and analysis on the global distribution of SFGR and associated ecological drivers based on all publicly available data up to March, 2021. The increasing research attention on SFGR has led to the accumulation of much data that made this study possible.

Our findings highlight the crucial role of ticks as the primary reservoir and vector in the spread of SFGR. Rickettsiae survival could rely on efficient transstadial and transovarial transmission among ticks.^1,21^ By contrast, other arthropods including fleas, mosquitoes, mites, lice, keds, and bugs might have less important roles in the ecology of SFGR species. Remarkably, although most vector ticks have well defined ecological niches due to their adaptations to local environments, some ticks have expanded their habitats in recent decades, largely due to climate changes and human activities.^22,23^ These dynamic changes present new and increasing threats of tick-borne rickettsioses to humans, livestock, and wild animals. For example, the emergence of *Ha longicornis* in eight states of the USA suggests an increasing spread, and highlights the need for close monitoring of the ticks and related rickettsiae in these regions.^24,25^

The diagnosis of SFG rickettsioses is traditionally based on the patient’s history of tick bite and a physical examination of fever, rash, and eschar. There is often no specific presentation of the disease in its early course, making it challenging to diagnose the disease and differentiate the etiological pathogen. Our summary of clinical symptoms by major rickettsiae species could improve diagnosis of rickettsioses (appendix 1, pp 6, 21–22). Also, if the attacking tick species can be identified, it is possible to narrow down the potential pathogen (appendix 1 p 37). The species-specific distribution and risk maps presented in this study could also be valuable to diagnosis of rickettsioses and control of rickettsiae. Therefore, *R rickettsii* could be considered with priority when diagnosing suspected cases of rickettsiae in the Americas, as could *R conorii* in Europe and *R africae* in Africa.^26^ *R amblyommii, R parkeri,* and *R rhipicephali* share a similar distribution to *R rickettsii* in the Americas indicated by cluster V, in a similar way to that of *R massiliae* and *R aeschlimannii* in Europe, which should also be considered when facing suspected cases. Moreover, *R felis* infection should be considered the first diagnosis if the symptoms of spotted fever occurred without history of tick exposure or field activities, as *R felis* is globally distributed and transmitted by fleas.

The ecological niches for SFGR are complex. For example, *R sibirica*, *R heilongjiangensis*, and *Candidatus* R tarasevichiae thrive in cooler environments, whereas others prefer a warm temperature ranging from 10 to 30°C. It is therefore meaningful to group SFGR species by their ecological characteristics to better understand the overall risk of rickettsia exposure at any given place. We found five clusters of tick species that share similar ecological niches and geographical distributions. Such clustering offers additional information for risk assessment and field investigation. For instance, despite the low detection of *R monacensis*, *R sibirica*, *R raoultii*, and *R slovaca* in Africa (appendix 1 pp 71, 73, 76–77), they should be targets for survey in this region because they are grouped together with *R felis,* which has high prevalence of field detection and model-predicted risks.^27^ We predicted high risks in Africa for 15 rickettsiae, but only 13 rickettsiae have been reported and five were found at no more than ten locations. Given the diverse climates and an abundance of animals and vegetations in Africa, rickettsial infections were probably under-detected in this continent.^28^ Therefore, even with a comprehensive literature search, there is a high chance of underrepresentation of low-income countries in Africa for some easily neglected rickettsiae, such as *R sibirica* and *R helvetica*. Although our ecological models at the global scale might not correct for surveillance and reporting bias, they reveal potential high-risk areas that have been neglected before, especially in low-resource countries.

Our study has several limitations. In the modelling analyses, the time range of the ecological variables does not fully align with that of the reported SFGR occurrences. However, 99·8% of the occurrence records of the 17 major SFGR species were collected during or after the 1980s, similar to the time period of the ecological variables used in the modelling analysis (climate data 1980–2018, leaf area index 1981–2019; land cover 1992–2019). We note that the use of average ecological predictors and cumulative presence or absence over multiple years in our ecological modelling represents an overall assessment of long-term, suitable environmental conditions but ignores possible temporal evolution; similarly, averaging covariates within polygons when polygons are much larger than the model resolution (10 × 10 km^2^) could lead to ecological fallacy if the covariates are distributed highly unevenly within the polygon. The quality of field detection and reporting of SFGR infection varies by country and region, and our analysis might be biased by paucity of laboratory testing and reporting capacities in many low-income countries. Therefore, some pseudo-absence locations could be false negative, ie, having undetected existing species in the past or emerging new species in the future, which implies potentially under-estimated risks. Furthermore, debates are ongoing about the taxonomy for some species, which could affect the reliability of mapping and modelling for these species.

Despite the caveats mentioned above, this study provides an evidence-based, up-to-date, global picture of the distributions and ecological drivers of SFGR, together with a comprehensive assembly of SFGR occurrence data at the global scale for future research. In the future, the distribution and ecology of SFGR will continue to evolve, which should be closely monitored with data collected from properly designed field surveys of vectors and animal hosts, improved surveillance systems of human cases, and periodic serosurveys in healthy populations.

## Supplementary Material

1

2

3

## Figures and Tables

**Table:** The average AUC for test sets of the BRT models and model-predicted areas and population sizes with medium to high risk of exposure to the 17 SFGR species

	Average testing AUC (2·5-97·5% percentiles)	Population size (million)					Area (10 000 km^2^)				
		Eurasia	Africa	Americas	Oceania	Worldwide	Eurasia	Africa	Americas	Oceania	Worldwide
*Rickettsia felis*	0·936 (0·910–0·955)	2936·3 (2513·9–3504·2)	720·7 (572·7–924·5)	764·8 (665·8–897·7)	22·7 (17·2–27·6)	4444·4 (3812·8–5338·3)	602·8 (463·2–778·1)	283·6 (207·7–420·1)	602·8 (479·4–761·4)	34·1 (24·9–48·4)	1523·3 (1203·1–2004·5)

*Rickettsia conorii*	0·949 (0·911–0·973)	2695·5 (2046·6–3283·3)	582·7 (442·9–711·8)	394·9 (255·9-513–4)	21·4 (15·1–25·0)	3694·5 (2789·9–4498·3)	570·2 (435·8–718·3)	232·7 (156·6–323·5)	251·0 (150·0–376·4)	67·3 (47·7–101·6)	1121·3 (775·8–1499·1)

*Rickettsia africae*	0·943 (0·882–0·973)	2355·3 (1727·6–3015·7)	998·0 (691·7–1170·4)	250·8 (149·1–369·5)	14·4 (9·8–20·0)	3618·6 (2574·7–4426·9)	338·1 (220·6–502·1)	449·7 (278·6–628·1)	147·5 (75·2–277·8)	51·1 (31·6–90·1)	986·5 (625·8–1450·8)

*Rickettsia sibirica*	0·958 (0·928–0·986)	1955·6 (1398·9–2359·7)	156·5 (99·9–294·1)	404·2 (224·8–613·7)	6·8 (4·2–12·0)	2523·2 (1831·5–3275·7)	658·1 (477·0–864·7)	59·6 (35·4–125·3)	481·7 (300·6–734·6)	20·5 (12·9–37·7)	1220·0 (861·2–1756·8)

*Rickettsia rickettsii*	0·961 (0·934–0·980)	729·1 (489·2–1322·0)	498·5 (384·1–680·1)	756·7 (594·3–890·7)	13·6 (8·3–18·8)	1998·0 (1514·4–2779·8)	159·4 (96·5–318·3)	300·7 (200·6–474·7)	655·4 (468·5–878·0)	17·1 (9·4–31·2)	1132·5 (816·5–1659·6)

*Rickettsia massiliae*	0·950 (0·894–0·986)	1276·1 (862·1–1809·4)	492·7 (338·9–683·9)	211·5 (132·1–324·3)	11·8 (6·9–17·5)	1992·0 (1365·1–2796·1)	310·3 (210·4–462·7)	154·1 (101·8–253·2)	106·8 (55·8–214·3)	62·7 (31·2–91·8)	633·9 (422·5–980·3)

*Rickettsia slovaca*	0·972 (0·938–0·993)	1293·0 (844·9–1875·4)	219·1 (81·6–507·5)	332·0 (179·2–606·6)	5·9 (3·2–13·4)	1850·0 (1120·3–2858·7)	576·9 (396·3–827·5)	96·5 (42·8–233·0)	357·1 (210·1–654·5)	66·9 (33·7–95·8)	1097·4 (696·9–1729·5)

Rickettsia raoultii	0·949 (0·917–0·972)	1039·0 (619·4–1600·5)	301·6 (131·9–488·5)	468·1 (278·6–639·6)	1·2 (0·5–3·3)	1809·9 (1133·2–2610·2)	571·0 (374·3–881·5)	171·9 (72·9–345·7)	709·5 (370·4–1139·4)	6·5 (1·8–25·4)	1458·9 (918·9–2322·6)

*Rickettsia amblyommii*	0·950 (0·913–0·970)	1061·8 (627·1–1578·7)	259·2 (152·8–411·3)	390·4 (281·4–471·8)	2·5 (1·1–6·5)	1713·8 (1077·3–2440·8)	281·2 (174·3–442·6)	159·8 (88·5–288·2)	377·4 (278·4–505·0)	7·7 (3·8–17·8)	826·1 (565·1–1246·7)

*Rickettsia monacensis*	0·954 (0·919–0·978)	1408·3 (1015·3–1966·3)	83·3 (50·0–160·6)	145·1 (86·6–277·2)	11·2 (5·9–17·9)	1647·8 (1161·4–2466·2)	503·3 (374·1–693·3)	19·8 (12·3–37·8)	118·4 (72·6–207·8)	25·6 (14·3–42·6)	667·1 (479·8–942·1)

*Rickettsia aeschlimannii*	0·969 (0·919–0·989)	852·8 (531·9–1257·0)	416·4 (343·2–523·6)	77·6 (31·3–164·5)	8·6 (5·3–12·0)	1355·5 (919·8–1916·2)	448·4 (261·9–638·6)	210·8 (138·9–334·1)	45·8 (20·2–109·7)	65·9 (33·3–90·1)	771·0 (475·0–1159·3)

*Rickettsia heilongjiangensis*	0·964 (0·898–0·995)	942·9 (513·7–1934·4)	175·3 (44·4–488·5)	194·5 (73·5–397·7)	3·0 (0·7–8·5)	1315·7 (617·2–2774·8)	556·9 (337·3–851·4)	92·3 (22·4–260·0)	359·7 (163·2–708·2)	21·0 (6·5–39·3)	1030·0 (527·2–1806·7)

*Rickettsia japonica*	0·942 (0·864–0·997)	933·3 (673·7–1423·9)	40·4 (24·2–152·6)	155·4 (76·2–339·5)	9·7 (5·5–20·0)	1138·9 (798·1–1894·4)	249·2 (167·6–411·0)	36·6 (16·0–223·2)	188·2 (81·6–451·1)	29·6 (17·6–71·5)	503·7 (282·8–1077·9)

*Rickettsia parkeri*	0·973 (0·947–0·989)	372·8 (246·8–620·4)	72·3 (40·8–188·8)	578·8 (463·2–704·6)	5·3 (3·7–8·0)	1029·2 (795·6–1504·7)	37·1 (23·4–78·6)	21·5 (11·1–59·6)	536·8 (415·3–681·3)	3·6 (2·4–6·5)	599·0 (464·1–822·5)

*Rickettsia rhipicephali*	0·961 (0·894–0·992)	147·5 (58·9–490·3)	146·8 (64·4–275·9)	475·9 (285·7–646·7)	1·9 (0·3–10·4)	772·2 (427·3–1347·1)	55·5 (22·2–174·7)	57·5 (26·3–136·2)	461·8 (249·3–746·2)	5·4 (1·8–22·8)	580·2 (307·5–1048·9)

*Rickettsia helvetica*	0·984 (0·975–0·992)	517·4 (427·8–659·6)	31·0 (14·8–69·3)	192·1 (118·3–296·8)	6·0 (4·0–8·4)	746·4 (559·8–1034·3)	342·0 (281·1–431·7)	12·7 (5·3–31·3)	88·2 (43·9–175·8)	16·4 (9·0–27·3)	459·3 (345·2–660·6)

*Candidatus* Rickettsia tarasevichiae	0·969 (0·906–0·996)	77·9 (44·1–391·8)	0·4 (0·0–30·3)	1·5 (0·4–15·0)	0	79·8 (44·8–433·8)	249·2 (142·1–457·0)	0·0 (0·0–7·9)	31·3 (8·9–82·3)	0·0 (0·0–2·0)	280·6 (244·6–593·0)

AUC=area under the curve. BRT=boosted regression trees. SFGR=spotted fever group rickettsiae.

## Data Availability

All the data collected in this study are available in the appendices.

## Research in context

### Evidence before this study

On Nov 1, 2021, we searched PubMed, Web of Science, MedRvix, and bioRvix for all papers using the search terms (“spotted fever” [All Fields] OR “spotted fever group *Rickettsia*” [All Fields] OR “SFG *Rickettsia*” [All Fields]) AND (“spatial” [All Fields] OR “distribution” [All Fields]) with no date or language restrictions. Among the 156 papers found, 63 focused on field studies of vectors, animals, or humans, 36 were related to epidemiology, 36 reported genomic studies, and 21 were related to spatial analyses of spotted fever group rickettsiae (SFGR). Among the 21 spatial analyses, 13 studies described geospatial distributions of SFGR in specific countries or smaller areas. The other five spatial studies were done at the continent level or in a specific ecological zone with multiple countries. The remaining three studies reported review or meta-analyses of the global distribution of SFGR, but none involved ecological modelling or risk mapping. Satjanadumrong and colleagues (2019) discussed the organisms causing rickettsial spotted fever and related diseases, their arthropod vectors in Asia, and the impact of change in land use on their spread without modelling efforts. Blanton and colleagues (2019) updated the epidemiology, clinical manifestations, diagnosis, treatment, and prevention of diseases caused by organisms in the genus *Rickettsia* but did not summarise the data of reported human cases. To date, no study has quantified SFGR risk at the global scale with a high geospatial resolution.

### Added value of this study

In this study, we geolocated reported occurrences of SFGR at a 10 km × 10 km resolution based on 1565 published studies on detections of SFGR in vectors, animals, or humans between 1906 and 2021. We analysed the relationship between vectors and rickettsiae. We mapped the spatial distributions of reported locations of different arthropod vectors carrying SFGR and those of 48 confirmed *Rickettsia* spp. worldwide. Using ecological machine-learning models, we assessed contributions of potential environmental, ecoclimatic, and biological drivers to the spatial distributions of the 17 predominant *Rickettsia* spp. and predicted areas and size of the at-risk populations to these rickettsiae. These 17 predominant *Rickettsia* spp. form five spatial clusters, each representing unique combinations of environmental and ecoclimatic features.

### Implications of all the available evidence

Our work offers a comprehensive and up-to-date picture of the global distributions of SFGR. The potential risk areas of SFGR are more extensive than have been reported, indicating the need for additional surveillance of SFG rickettsial infections, especially in low-resource countries.
