# Supplementary material for: Mapping the global distribution of spotted fever group rickettsiae: a systematic review with modelling analysis
Source: Lancet Digit Health. Author manuscript; Available in PMC 2023 Mar 25. (PMC10039616; doi:10.1016/S2589-7500(22)00212-6)

THE LANCET
Digital Health

Supplementary appendix 1

This appendix formed part of the original submission and has been peer reviewed. We post it as supplied by the authors.

Supplement to: Zhang Y-Y, Sun Y-Q, Chen J-J, et al. Mapping the global distribution of spotted fever group rickettsiae: a systematic review with modelling analysis. *Lancet Digit Health* 2022; published online Nov 21. https://doi.org/10.1016/S2589-7500(22)00212-6.

Supplementary appendix

Supplement to: YY Zhang, et al. Mapping global distribution of spotted fever group rickettsiae: a systematic review with modeling analysis

Continent

Supplementary Methods 3
Supplementary Results 6
Supplementary table 1: The inclusion and exclusion criteria of screening publications 7
Supplementary table 2: The laboratory tests used to detect SFGR infections in the reviewed studies..... 8
Supplementary table 3: List of variables extracted from reviewed studies 9
Supplementary table 4: Vectors with evidence of biting human 10
Supplementary table 5: Original resolutions and extents of source datasets 15
Supplementary table 6: Variables used for ecological modeling of SFGR in this study 16
Supplementary table 7: The references for all the SFGR species 17
Supplementary table 8: The co-infection of SFGR species and their infected vectors. 19
Supplementary table 9: The number of different infection types of pathogenic rickettsiae 20
Supplementary table 10: Clinical characteristics of human infections with different SFGR species in the world. 21
Supplementary table 11: BRT-model-estimated mean (95% percentiles) relative contributions of top factors (RC>3%) to the spatial distribution of 17 SFGR. 23
Supplementary table 12: BRT and RF model-estimated mean (95% percentiles) relative contributions of top five factors to the spatial distribution of 17 SFGR. 25
Supplementary table 13: BRT-model-estimated mean (95% percentiles) relative contributions of top factors (RC>3%) to five clusters. 27
Supplementary figure 1: Correlation matrices of ecological variables used for screening multicollinearity before modeling the predominant 17 SFGR species. 28
Supplementary figure 2: The relationship matrix of SFGR species and involved ticks. 37
Supplementary figure 3: The relationship matrix of SFGR species and other vectors. 38
Supplementary figure 4: The relationship matrix of SFGR species and animals. 39
Supplementary figure 5: SFGR species richness (red circles) at the country level in four continents. .. 40
Supplementary figure 6: The globally geographical distributions of the non-predominant 31 SFGR species. 41
Supplementary Figure 7: Comparing predictive performance of the three machine-learning algorithms. 42
Supplementary Figure 8: Effects of major predictors (relative contributions >3%) for presence of *R. aeschlimannii* based on BRT models. 45
Supplementary Figure 9: Effects of major predictors (relative contributions >3%) for presence of *R. africae* based on BRT models. 46
Supplementary Figure 10: Effects of major predictors (relative contributions >3%) for presence of *R. amblyommii* based on BRT models. 47
Supplementary Figure 11: Effects of major predictors (relative contributions >3%) for presence of *R. conorii* based on BRT models. 48
Supplementary Figure 12: Effects of major predictors (relative contributions >3%) for presence of *R. felis* based on BRT models. 49
Supplementary Figure 13: Effects of major predictors (relative contributions >3%) for presence of *R. heilongjiangensis* based on BRT models. 50
Supplementary Figure 14: Effects of major predictors (relative contributions >3%) for presence of *R. helvetica* based on BRT models. 51
Supplementary Figure 15: Effects of major predictors (relative contributions >3%) for presence of *R. japonica* based on BRT models. 52
Supplementary Figure 16: Effects of major predictors (relative contributions >3%) for presence of *R. massiliae* based on BRT models. 53
Supplementary Figure 17: Effects of major predictors (relative contributions >3%) for presence of *R. monacensis* based on BRT models. 54
Supplementary Figure 18: Effects of major predictors (relative contributions >3%) for presence of *R. parkeri* based on BRT models. 55
Supplementary Figure 19: Effects of major predictors (relative contributions >3%) for presence of *R. raoultii* based on BRT models. 56

Supplementary Figure 20: Effects of major predictors (relative contributions >3%) for presence of *R. rhipicephali* based on BRT models. 57
Supplementary Figure 21: Effects of major predictors (relative contributions >3%) for presence of *R. rickettsii* based on BRT models. 58
Supplementary Figure 22: Effects of major predictors (relative contributions >3%) for presence of *R. sibirica* based on BRT models. 59
Supplementary Figure 23: Effects of major predictors (relative contributions >3%) for presence of *R. slovaca* based on BRT models. 60
Supplementary Figure 24: Effects of major predictors (relative contributions >3%) for presence of *Candidatus* R. tarasevichiae based on BRT models. 61
Supplementary figure 25: The global recorded and predicted distributions of *R. aeschlimannii*. 62
Supplementary figure 26: The global recorded and predicted distributions of *R. africae*. 63
Supplementary figure 27: The global recorded and predicted distributions of *R. amblyommii*. 64
Supplementary figure 28: The global recorded and predicted distributions of *R. conorii*. 65
Supplementary figure 29: The global recorded and predicted distributions of *R. felis*. 66
Supplementary figure 30: The global recorded and predicted distributions of *R. heilongjiangensis*. 67
Supplementary figure 31: The global recorded and predicted distributions of *R. helvetica*. 68
Supplementary figure 32: The global recorded and predicted distributions of *R. japonica*. 69
Supplementary figure 33: The global recorded and predicted distributions of *R. massiliae*. 70
Supplementary figure 34: The global recorded and predicted distributions of *R. monacensis*. 71
Supplementary figure 35: The global recorded and predicted distributions of *R. parkeri*. 72
Supplementary figure 36: The global recorded and predicted distributions of *R. raoultii*. 73
Supplementary figure 37: The global recorded and predicted distributions of *R. rhipicephali*. 74
Supplementary figure 38: The global recorded and predicted distributions of *R. rickettsii*. 75
Supplementary figure 39: The global recorded and predicted distributions of *R. sibirica*. 76
Supplementary figure 40: The global recorded and predicted distributions of *R. slovaca*. 77
Supplementary figure 41: The global recorded and predicted distributions of *Candidatus* R. tarasevichiae. 78
Supplementary figure 42: The global recorded distributions of six species rickettsiae. 79
Supplementary figure 43: The global recorded distributions of other six species rickettsiae. 80
Supplementary figure 44: The global recorded distributions of three species rickettsiae. 81
Supplementary figure 45: The global recorded distributions of six species *Candidatus* rickettsiae. 82
Supplementary figure 46: The global recorded distributions of other six species *Candidatus* rickettsiae. 83
Supplementary figure 47: The global recorded distributions of four species *Candidatus* rickettsiae. 84
Supplementary figure 48: Clustering of SFGR species based on their ecological features (A) and spatial distributions of the clusters (B-F). 85

Supplementary Methods

Data extraction

The following data were extracted from each selected article (appendix p 9): article title, authors, publication year, study period (sample collection time), study site (up to the highest resolution), latitude and longitude of the site, species of SFGR, detection method, type of host (arthropod [species], animal [species] or human being), the total number of tested samples, and the number of positive samples for each tested SFGR species. By searching previous databases, we found a total of 115 species of vectors with evidence of biting humans (appendix pp 10–14).

Geo-positioning of the occurrence data

To geocode occurrences of SFGR species, an occurrence is defined as one or more confirmed infection(s) with any SFGR species at a unique location (geocoordinates, polygons, or 10 km×10 km pixels) during any period, regardless of the type of the host or the time of detection. Serology studies were excluded when modeling due to potential cross-reactivity between SFGR species. Whenever available, we extracted geocoordinates from peer-reviewed articles reporting confirmed SFGR occurrences (detected by molecular assay or pathogen isolation) as "point" data. When point information was not available, we extracted the location as a two-dimensional bounded region, or a "polygon". A polygon is usually an administrative unit, such as a county, a city or a province. Occasionally, it could be a customized sampling region. For each polygon, the coordinates of its geographic centroid were queried from Google Maps. All location data were geopositioned with the highest possible precision and checked to ensure coordinates were accurate and duplicates were removed, so that each individual record used in our model represents a unique occurrence of SFGR detection. After that geocoordinates (latitude and longitude) of sites were second queried using Google Maps to make sure they match the locations mentioned in the articles as well as to remove duplicated sites. All occurrence data underwent quality control to ensure reliability and precision of geo-positioning. Specifically, all occurrence data were double checked by two investigators (YQS, JJC) independently, with special attention to the SFGR species and their sampling times and locations. In addition, names of study sites were updated if current names slightly differ from the ones used in the articles. In addition, classification of locations of occurrence as "Points" or "Polygons" were cross-checked by the two investigators.

Assembling occurrence data and covariates

We created a global grid-map with a resolution of 10km×10km using ArcGIS 10.7 (Esri Inc, Redlands, CA, USA) and then associated each grid with ecological variables. Each occurrence was matched to the grid-map according to its coordinate. For polygon-type occurrence records, we assigned the grid containing the centroid of the polygon as the occurrence grid. Only one occurrence was counted if multiple records were associated with the same grid. For ecological modeling, we need to associate ecological variables with each grid (occurrence or non-occurrence). The average of each ecological variable over its corresponding time span was calculated for each grid.[1] If the original occurrence record is a point location, association of point data with grids is straightforward and no more processing is needed. If the original occurrence record is a polygon, the assigned occurrence grid may not be the true location, and the ecological variables associated with that grid may not represent the true ecological condition for that occurrence. To minimize potential ecological fallacy, we first exclude all polygon occurrence records with an area larger than 1°×1° (14·1%, 228/1620) from ecological modeling because of insufficient resolution.[2] For polygon occurrence records with an area no larger than 1°×1°, we calculated the mean of each ecological variable across all grids within the polygon and associated the mean value with the occurrence grid, i.e., the grid containing the centroid of the polygon. All occurrence grids were considered as "cases". For each occurrence grid we sample pseudo-absence grids as "controls" with a case-to-control ratio of 1:3 for the modelling analysis.[3,4] For each occurrence grid, the sampling was restricted to a circle centered around the centroid of this occurrence grid, with a radius determined by the shortest distance to the centroids of other occurrence grids. The radius used ranges from 30km to 3000km across occurrence locations.

Clinical spectrum of rickettsioses

We focused on SFGR species with 10 or more human cases for whom symptoms were recorded to minimize the impact of small sample size. For each species, we summarized the frequency of each symptom and its proportion among all known symptoms. As it is likely that only prevalent symptoms were reported in many published studies, we estimated the number of each unreported symptom in each study in two ways: 1) frequency is 0, assuming the symptom was absent if not reported; and 2) frequency was the same as the minimum frequency among all reported symptoms. These two ways give us a lower bound and an upper bound for the frequency and proportion of each symptom for each pathogen. Here we define a symptom as a major symptom if the lower bound of proportion is ⩾30%.

BRT model

To avoid overfitting and to improve interpretability of the models, we first screened for multicollinearity among candidate predictors.[5] We first selected BIO1 (annual average temperature) and BIO12 (annual precipitation), the two most commonly used biological variables with easy interpretation, to be included in the models.[6] We then examined the correlation between these two variables and all other predictors and excluded those with correlation coefficients greater than 0·70. Among the remaining predictors, we further assessed correlations between all possible pairs, and we removed one variable from each pair according to the following criteria: (1) the variable highly correlated (correlation coefficient >0·70) with more of other variables; and (2) the variable that is less correlated with the outcome (appendix pp 29−37). We then fitted an initial model for each species, and predictors with relative contributions (RCs) greater than 3% were retained for the formal model-building. In the final model, we randomly divided the data into an 80% training set and a 20% test set and fitted a BRT model, which was repeated 100 times.[6,7] That is, we obtained 100 models based on the 100 training datasets for each target species, to which we refer as a model assembly. Using these presence and pseudo-absence locations and ecological predictors, BRT models were fitted using the 'gbm.step' function in "dismo" package in R 4.0.3 (R Foundation for Statistical Computing, Vienna, Austria) with a tree complexity of five, a learning rate of 0·005, and a bagging fraction of 75% based on their satisfactory performance in our previous research.[1,8,9] A 10-fold cross validation was used to identify the optimal number of trees using the gbm.step function in the R package "dismo". Due to both the data size (40 predictors) and the number of models runs ([17 SFGR species] × 100), we cannot afford a full cross-validation optimization for all model configuration parameters. However, we performed a sensitivity analysis using a learning rate of 0.01 for selected SFGR species but found no substantial difference in the contribution estimates.

Random Forest

RF (Random Forest) is another classical ensemble learning model widely used.[10] The training algorithm of RF is based on bootstrap aggregating. Each tree is trained on many bootstrap samples, and was then evaluated using the remaining data to produce more accurate classifications. The unknown class of an observation will be calculated by the majority vote of the out-of-bag predictions for that observation.[11] We optimized the key learning parameter, mtry, which defines the number of variables randomly sampled as candidates at each split, between the range of 2–20 in random search, with a 10-fold cross-validation process to avoid overfitting. The R packages "caret" and "randomForest" were used to develop the random forest model.

LASSO regression

We used L1-penalized least absolute shrinkage and selection regression for multivariable analyses, augmented with 10-fold cross validation for internal validation.[12] This is a logistic regression model that penalizes the absolute size of the coefficients, where the sum of absolute values of coefficients is multiplied by a weight coefficient λ and then added to the traditional loss function. With larger penalties, the estimates of weaker factors shrink toward zero, so that only the strongest predictors remain in the model. The optimal λ was chosen via 10-fold cross validation to minimize the average misclassification error. Subsequently, variables identified by LASSO regression analysis were entered into traditional logistic regression models without penalty (as there is no predictor of more interest than

others, double selection was not performed). The package “glmnet” in R was used to perform the LASSO regression, and optimal λ was chosen using the cv.glmnet function.

Model evaluation
Similar to the BRT, we obtained 100 models as a model assembly for RF and LASSO as well by random splitting the data into training and test sets. The RCs of all predictors and the AUCs for test sets were averaged over the 100 models in the assembly to represent the final estimation results and predictive performance of the model assembly. We selected the best algorithm in terms of the highest average test AUC to map the global distribution of SFGR. To determine model-predicted high-risk areas for each SFGR species, we chose a cut-off value that maximizes sensitivity + specificity along the average test ROC curve of the model assembly of the chosen algorithm.[13-15] Grids with an average predicted probability (over the 100 models) above the cut-off value were considered as having a high risk of presence of the corresponding rickettsiae species. For each species, the area and population size of model-predicted high-risk areas were calculated.

References

1 Zhao GP, Wang YX, Fan ZW, et al. Mapping ticks and tick-borne pathogens in China. *Nat Commun* 2021; **12:** 1075.
2 Messina JP, Pigott DM, Golding N, et al. The global distribution of Crimean-Congo hemorrhagic fever. *Trans R Soc Trop Med Hyg* 2015; **109:** 503-13.
3 Barbet-Massin M, Jiguet F, Albert CH, Thuiller W. Selecting pseudo-absences for species distribution models: how, where and how many? *Methods Ecol Evol* 2012; **3:** 327-38.
4 Vanderwal J, Shoo L, Graham C, et al. Selecting pseudo-absence data for presence-only distribution modeling: How far should you stray from what you know? *Ecol Modell* 2009; **220:** 589-594.
5 Miao D, Dai K, Zhao GP, et al. Mapping the global potential transmission hotspots for severe fever with thrombocytopenia syndrome by machine learning methods. *Emerg microbes Infect* 2020; **9:** 817-26.
6 Che TL, Jiang BG, Xu Q, et al. Mapping the risk distribution of *Borrelia burgdorferi* sensu lato in China from 1986 to 2020: a geospatial modelling analysis. *Emerg Microbes Infect* 2022; **11:** 1215-26.
7 Shearer FM, Longbottom J, Browne AJ, et al. Existing and potential infection risk zones of yellow fever worldwide: a modelling analysis. *Lancet Glob Health* 2018; **6:** e270-e78.
8 Chen WJ, Lai SJ, Yang Y, et al. Mapping the distribution of Anthrax in mainland China, 2005-2013. *PLoS Negl Trop Dis* 2016; **10:** e0004637.
9 Yao H, Wang Y, Mi X, et al. The scrub typhus in mainland China: spatiotemporal expansion and risk prediction underpinned by complex factors. *Emerg Microbes Infect* 2019; **8:** 909-19.
10 Walter M, Vogelgesang JR, Rubel F, Brugger K. Tick-borne encephalitis virus and its European distribution in ticks and endothermic mammals. *Microorganisms* 2020; **8**: 1065.
11 Zhang J, Yue M, Hu Y, et al. Risk prediction of two types of potential snail habitats in Anhui Province of China: Model-based approaches. *PLoS Negl Trop Dis* 2020; **14:** e0008178.
12 Liang W, Liang H, Ou L, et al. Development and validation of a clinical risk score to predict the occurrence of critical illness in hospitalized patients with COVID-19. *JAMA* 2020; **180:** 1081-89.
13 Wang T, Fan ZW, Ji Y, et al. Mapping the distributions of mosquitoes and mosquito-borne arboviruses in China. *Viruses* 2022; **14:** 691.
14 Schisterman EF, Perkins NJ, Liu A, Bondell H. Optimal cut-point and its corresponding Youden Index to discriminate individuals using pooled blood samples. *Epidemiology* 2005; **16:** 73-81.
15 Ruopp MD, Perkins NJ, Whitcomb BW, Schisterman EF. Youden Index and optimal cut-point estimated from observations affected by a lower limit of detection. *Biom J* 2008; **50:** 419-30.

Supplementary Results

Detection of SFGR in arthropod vectors

Six species of soft ticks belonging to three genera and 140 species of hard ticks in seven tick genera were found to harbor six and 46 SFGR species, respectively. Among those hard ticks that carry SFGR, genera *Ixodes* harbored the highest variety of SFGR (32 species carried by 24 *Ixodes* spp.), followed by *Haemaphysalis* (28 species by 22 *Haemaphysalis* spp.), *Dermacentor* (24 species carried by 11 *Dermacentor* spp.), *Amblyomma* (22 species carried by 47 *Amblyomma* spp.), *Rhipicephalus* (19 species carried by 22 *Rhipicephalus* spp.) and *Hyalomma* (10 species carried by 13 *Hyalomma* spp.). When considering tick species that bite humans and SFGR species that are pathogenic in humans, high risks to humans were posed by the following tick genera: *Amblyomma* (31 human-biting tick species harboring 10 pathogenic SFGR species), *Haemaphysalis* (12 harboring 19), *Ixodes* (12 harboring 17), *Dermacentor* (eight harboring 17), *Rhipicephalus* (10 harboring 14), and *Hyalomma* (12 harboring eight). Full details about vectors and SFGR species they carry were given in the appendix pp 38–39.

Among the 12 SFGR species found in two or more vector types, *R. felis* was infecting the most vector types (six: ticks, fleas, mosquitoes, mites, lice and bug), followed by *R. helvetica* (four: ticks, fleas, mites and keds), *R. monacensis* (three: ticks, fleas and mosquitoes) and *R. asembonensis* (three: ticks, fleas and lice). A few studies reported simultaneous detection of two SFGR species in a single tick, involving 12 different coinfection pairs of rickettsiae detected in 11 species of vectors. Coinfections of *Candidatus* R. tarasevichiae and *R. raoultii* were found in both *Dermacentor silvarum* and *Haemaphysalis concinna*, and coinfections of all pairs among *R. heilongjiangensis*, *Candidatus* R. tarasevichiae and *R. raoultii* were found in *Ha. concinna* (appendix p 19).

Detection of SFGR in animals

Among the seven SFGR-carrying livestock, dogs harbored the highest variety of SFGR (11 species), followed by camels (five) and cattle (five). Among all identified SFGR carried by rodents, *R. felis* infected the greatest number of rodent species (10 species), followed by *R. helvetica* (nine), *R. sibirica* (eight), and *R. conorii* (six). Taken together, *R. felis* and *R. helvetica* were found in the most types of wild animals (16 species), followed by *R. conorii* (11) and *R. sibirica* (eight). Additionally, *R. raoultii* was detected in the most types of livestock (five), followed by *R. felis* (four) and *R. slovaca* (four). Full details of SFGR and the infected animals were shown in appendix p 40.

Clinical spectrum of rickettsioses

There were 15 SFGR species confirmed in 10 or more human cases with clinical symptoms recorded, yielding a total number of 7477 patients with symptom profiles. Considering spotted fever group rickettsioses caused by any of the 15 SFGR species, cutaneous rash (83·4–85·1%) and fever (74·8–77·4%) were the two most common symptoms of SFG rickettsioses, followed by myalgia (49·0–56·2%), headache (46.9–57.4%) and eschar (46.1–55.1%), shown on appendix p 21. Fever was a major symptom for all 15 species, and headache, eschar, myalgia and cutaneous rash were major symptoms for 11, 10, 10 and eight species, respectively (appendix pp 21–22). Malaise and lymphadenopathy were major symptoms for five species. All other symptoms were major for no more than two species. In particular, gastrointestinal symptoms were rare.

Model-estimated variables and their RCs to the occurrence of the predominant 17 SFGR

The annual mean temperature was associated with a RC of ⩾10% for all 17 dominant SFGR species, with particularly high RCs (24·7%–28·3%) for *R. conorii*, *R. parkeri*, and *Candidatus* R. tarasevichiae (appendix pp 23−24). An annual mean temperature in the range of 10–30℃ was associated with a high probability of presence for most of the SFGR, with the exception of *R. sibirica*, *R. heilongjiangensis*, and *Candidatus* R. tarasevichiae for which <10℃ was preferred (appendix pp 46−62). Seven species, including *R. aeschlimannii*, *R. heilongjiangensis*, *R. helvetica*, *R. japonica*, *R. rhipicephali*, *R. sibirica*, and *R. slovaca*, were ecologically impacted by one or more of four precipitation-related variables (precipitation of warmest quarter, annual precipitation, precipitation seasonality and precipitation of coldest quarter).

Supplementary table 1: The inclusion and exclusion criteria of screening publications

Criteria	Guidance	Outcome
**Title/Abstract screening**		
#1: Pathogens	Does the Title/Abstract refer the pathogens which belong to SFGR?	If Yes, remain and evaluate #2. If No, exclude.
#2: Source of infection	Does the Title/Abstract refer the pathogens which are from natural environment?	If Yes, remain and evaluate #3. If No, exclude.
#3: Tested objects	Does the Title/Abstract refer the pathogens which are detected from vectors, animals or humans?	If Yes, remain and evaluate #4. If No, exclude.
#4: Not review	Does the Title/Abstract refer the article which is Not a review? (Not reviewing the published articles, with presenting new primary data)	If No, remain for full text review. If Yes, exclude.
**Full text screening**		
#1: Re-screening	Does the article meet the screening criteria before?<br>1-pathogens belong to SFGR<br>2-infection in natural environment<br>3-detected from vectors, animals or humans<br>4-not review<br>5-not drug or vaccine trials<br>6-not transstadial transmission research in vectors<br>7-not molecular research of rickettsia	If Yes, remain and evaluate #2. If No, exclude.
#2: Laboratory method	Does the article refer the specific detection methods?<br>1-detailed specimen used for testing (e.g. vectors or blood from animals or humans)<br>2-pathogen-based testing method (e.g. serological or molecular)<br>3-specific pathogen identified in the detection	If Yes, remain and evaluate #3. If No, exclude.
#3: Geographical information	Does the article refer the geographical information?<br>1-geographic location information at country or subnational administrative divisions levels<br>2-exact locations or only marked the latitude and longitude<br>3-explicit locations of getting infections when imported	If Yes, remain for data extracting. If No, exclude.

Supplementary table 2: The laboratory tests used to detect SFGR infections in the reviewed studies.

	Detection methods
**Infection in vectors**	(1) molecular detection and sequence determination;<br>(2) isolation and cultivation of pathogens from samples;<br>(3) light or electronic micrograph identification.
**Infection in animals**	(1) molecular detection with PCR or sequencing.
**Confirmed human cases**	(1) molecular detection and sequence determination;<br>(2) isolation and cultivation of pathogens from samples;<br>(3) light or electronic micrograph identification;<br>(4) a four-fold increase in titre of specific antibodies in blood sera collected from the acute and convalescent stages of illness, or a seroconversion of specific antibodies.
**Serological tests in humans**	Serological test with single sera sample.

Supplementary table 3: List of variables extracted from reviewed studies

Variables	Explanation
Reference ID	Unique identifier assigned to an article.
Article title	Article title that included in the review.
Authors	Authors of the included article.
Publication year	Publication year of the article eligible for inclusion.
Study period	The start and end time for the period over which the sample collected.
Study site	Sampling sites including three parts to be recorded, country, the detailed address in the article and coordinates. Coordinates of geographic center of the detailed locations was used if they are not provided by author.
Detection method	The technology used to detect specific *rickettsia*.
Pathogen name	Standard name of SFGR tested in the study.
Pathogen detected from vectors/animals	
Species of vectors/animals	The species of detected vectors/animals.
Number of tested all	The total number tested for specific pathogen.
Number of tested positive	The number tested as positive for specific pathogen.
Pathogen detected from human beings	
Study type	Studies are categorized as case report (described clinical features of every patient in detail) and case series (summarized the clinical characteristics of some confirmed patients).
Number of individuals tested all	The number of individuals tested all for specific pathogen.
Number of individuals tested positive	The number of individuals tested as positive for specific pathogen.
Clinical manifestation	Record the clinical manifestation of humans if they were only infected by one species of pathogen and infection type was confirmed infection.

Supplementary table 4: Vectors with evidence of biting human

Vector	Species	Reference
Tick	*Amblyomma americanum*	Madison-Antenucci S, Kramer LD, Gebhardt LL, Kauffman E. Emerging tick-borne diseases. *Clin Microbiol Rev* 2020; **33:** e00083-18.
	Amblyomma astrion	Petney TN, Horak IG, Rechav Y. The ecology of the African vectors of heartwater, with particular reference to *Amblyomma hebraeum* and *Amblyomma variegatum*. *Onderstepoort J Vet Res* 1987; **54:** 381-95.
	Amblyomma cohaerens	Petney TN, Horak IG, Rechav Y. The ecology of the African vectors of heartwater, with particular reference to *Amblyomma hebraeum* and *Amblyomma variegatum*. *Onderstepoort J Vet Res* 1987; **54:** 381-95.
	Amblyomma gemma	Petney TN, Horak IG, Rechav Y. The ecology of the African vectors of heartwater, with particular reference to *Amblyomma hebraeum* and *Amblyomma variegatum*. *Onderstepoort J Vet Res* 1987; **54:** 381-95.
	Amblyomma hebraeum	Petney TN, Horak IG, Rechav Y. The ecology of the African vectors of heartwater, with particular reference to *Amblyomma hebraeum* and *Amblyomma variegatum*. *Onderstepoort J Vet Res* 1987; **54:** 381-95.
	Amblyomma lepidum	Petney TN, Horak IG, Rechav Y. The ecology of the African vectors of heartwater, with particular reference to *Amblyomma hebraeum* and *Amblyomma variegatum*. *Onderstepoort J Vet Res* 1987; **54:** 381-95.
	Amblyomma marmoreum	Petney TN, Horak IG, Rechav Y. The ecology of the African vectors of heartwater, with particular reference to *Amblyomma hebraeum* and *Amblyomma variegatum*. *Onderstepoort J Vet Res* 1987; **54:** 381-95.
	Amblyomma variegatum	Petney TN, Horak IG, Rechav Y. The ecology of the African vectors of heartwater, with particular reference to *Amblyomma hebraeum* and *Amblyomma variegatum*. *Onderstepoort J Vet Res* 1987; **54:** 381-95.
	Amblyomma aureolatum	Szabó MP, Pinter A, Labruna MB. Ecology, biology and distribution of spotted-fever tick vectors in Brazil. *Front Cell Infect Microbiol* 2013; **3:** 27.
	Amblyomma cajennense	Szabó MP, Pinter A, Labruna MB. Ecology, biology and distribution of spotted-fever tick vectors in Brazil. *Front Cell Infect Microbiol* 2013; **3:** 27.
	Amblyomma dubitatum	Szabó MP, Pinter A, Labruna MB. Ecology, biology and distribution of spotted-fever tick vectors in Brazil. *Front Cell Infect Microbiol* 2013; **3:** 27.
	Amblyomma ovale	Szabó MP, Pinter A, Labruna MB. Ecology, biology and distribution of spotted-fever tick vectors in Brazil. *Front Cell Infect Microbiol* 2013; **3:** 27.
	Amblyomma coelebs	Ito K, Taniguchi H, Ohtaki N, Ando S, Kawabata H. A first case of tick bite by *Amblyomma* coelebs in Japan. *J Dermatol* 2018; **45:** 243-4.
	Amblyomma mixtum	Novakova M, Literak I, Chevez L, et al. Rickettsial infections in ticks from reptiles, birds and humans in Honduras. *Ticks Tick Borne Dis* 2015; **6:** 737-42.
	Amblyomma hadanii	Saracho-Bottero MN, Tarragona EL, Sebastian PS, et al. Ticks infesting cattle and humans in the Yungas Biogeographic province of Argentina, with notes on the presence of tick-borne bacteria. *Exp Appl Acarol* 2018; **74:** 107-16.
	Amblyomma sculptum	Saracho-Bottero MN, Tarragona EL, Sebastian PS, et al. Ticks infesting cattle and humans in the Yungas Biogeographic province of Argentina, with notes on the presence of tick-borne bacteria. *Exp Appl Acarol* 2018; **74:** 107-16.
	Amblyomma tonelliae	Saracho-Bottero MN, Tarragona EL, Sebastian PS, et al. Ticks infesting cattle and humans in the Yungas Biogeographic province of Argentina, with notes on the presence of tick-borne bacteria. *Exp Appl Acarol* 2018; **74:** 107-16.
	Amblyomma triguttatum	Graves SR, Stenos J. Tick-borne infectious diseases in Australia. *Med J Aust* 2017; **206:** 320-4.
	Amblyomma maculatum	Zemtsova GE, Watkins NE, JRhipicephalus, Levin ML. Multiplex qPCR assay for identification and differentiation of *Amblyomma americanum*, *Amblyomma cajennense*, and *Amblyomma maculatum* (Ixodida: Ixodidae) tick species in the eastern United States. *J Med Entomol* 2014; **51:** 795-803.
	Amblyomma imitator	Merino O, De la Cruz NI, Martinez J, et al. Molecular detection of *Rickettsia* species in ticks collected in the Mexico-USA transboundary region. *Exp Appl Acarol* 2020; **80:** 559-67.
	Amblyomma loculosum	Eldin C, Mediannikov O, Davoust B, et al. Emergence of *Rickettsia africae*, Oceania. *Emerg Infect Dis* 2011; **17:** 100-2.
	Amblyomma longirostre	Valente JDM, Silva PW, Arzua M, et al. Records of ticks (Acari: Ixodidae) on humans and distribution of spotted-fever cases and its tick vectors in Paraná State, southern Brazil. *Ticks Tick Borne Dis* 2020; **11:** 101510.
	Amblyomma parkeri	Valente JDM, Silva PW, Arzua M, et al. Records of ticks (Acari: Ixodidae) on humans and distribution of spotted-fever cases and its tick vectors in Paraná State, southern Brazil. *Ticks Tick Borne Dis* 2020; **11:** 101510.
	Amblyomma naponense	Bermúdez CS, Castro A, Esser H, et al. Ticks (Ixodida) on humans from central Panama, Panama (2010-2011). *Exp Appl Acarol* 2012; **58:** 81-8.
	Amblyomma oblongoguttatum	Bermúdez CS, Castro A, Esser H, et al. Ticks (Ixodida) on humans from central Panama, Panama (2010-2011). *Exp Appl Acarol* 2012; **58:** 81-8.

Vector	Species	Reference
Tick	*Amblyomma neumanni*	Bermúdez CS, Castro A, Esser H, Liefting Y, García G, Miranda RJ. Infection by *Rickettsia bellii* and *Candidatus* "Rickettsia amblyommii" in *Amblyomma neumanni* ticks from Argentina. *Microb Ecol* 2007; **54:** 126-33.
	Amblyomma tigrinum	Nava S, Lareschi M, Rebollo C, et al. The ticks (Acari: Ixodida: Argasidae, Ixodidae) of Paraguay. *Ann Trop Med Parasitol* 2007; **101:** 255-70.
	Amblyomma parvum	Monje LD, Fernandez C, Percara A. Detection of *Ehrlichia* sp. strain San Luis and *Candidatus* Rickettsia andeanae in *Amblyomma parvum* ticks. *Ticks Tick Borne Dis* 2019; **10:** 111-4.
	Amblyomma pseudoconcolor	Tomassone L, Nuñez P, Ceballos LA, Gürtler RE, Kitron U, Farber M. Detection of "*Candidatus* Rickettsia sp. strain Argentina" and *Rickettsia bellii* in *Amblyomma* ticks (Acari: Ixodidae) from Northern Argentina. *Exp Appl Acarol* 2010; **52:** 93-100.
	Amblyomma testudinarium	Chao LL, Lu CW, Lin YF, Shih CM. Molecular and morphological identification of a human biting tick, *Amblyomma testudinarium* (Acari: Ixodidae), in Taiwan. *Exp Appl Acarol* 2017; **71:** 401-14.
	Amblyomma triste	Romer Y, Borrás P, Govedic F, et al. Clinical and epidemiological comparison of *Rickettsia parkeri* rickettsiosis, related to *Amblyomma* triste and *Amblyomma tigrinum*, in Argentina. *Ticks Tick Borne Dis* 2020; **11:** 101436.
	Dermacentor variabilis	Madison-Antenucci S, Kramer LD, Gebhardt LL, Kauffman E. Emerging tick-borne diseases. *Clin Microbiol Rev* 2020; **33:** e00083-18.
	Dermacentor andersoni	Merino O, De la Cruz NI, Martinez J, et al. Molecular detection of *Rickettsia* species in ticks collected in the Mexico-USA transboundary region. *Exp Appl Acarol* 2020; **80:** 559-67.
	Dermacentor marginatus	Garcia-Vozmediano A, Giglio G, Ramassa E, Nobili F, Rossi L, Tomassone L. *Dermacentor marginatus* and *Dermacentor reticulatus*, and their infection by SFG Rickettsiae and Francisella-Like Endosymbionts, in mountain and periurban habitats of northwestern Italy. *Vet Sci* 2020; **7:** 157.
	Dermacentor nitens	Szabó MPJ, Martins TF, Barbieri ARM, et al. Ticks biting humans in the Brazilian savannah: attachment sites and exposure risk in relation to species, life stage and season. *Ticks Tick Borne Dis* 2020; **11:** 101328.
	Dermacentor nuttalli	Khasnatinov MA, Liapunov AV, Manzarova EL, Kulakova NV, Petrova IV, Danchinova GA. The diversity and prevalence of hard ticks attacking human hosts in Eastern Siberia (Russian Federation) with first description of invasion of non-endemic tick species. *Parasitol Res* 2016; **115:** 501-10.
	Dermacentor reticulatus	Khasnatinov MA, Liapunov AV, Manzarova EL, Kulakova NV, Petrova IV, Danchinova GA. The diversity and prevalence of hard ticks attacking human hosts in Eastern Siberia (Russian Federation) with first description of invasion of non-endemic tick species. *Parasitol Res* 2016; **115:** 501-10.
	Dermacentor occidentalis	Salkeld DJ, Porter WT, Loh SM, Nieto NC. Time of year and outdoor recreation affect human exposure to ticks in California, United States. *Ticks Tick Borne Dis* 2019; **10:** 1113-7.
	Dermacentor silvarum	Guo WB, Shi WQ, Wang Q, et al. Distribution of *Dermacentor silvarum* and associated pathogens: meta-analysis of global published data and a field survey in China. *Int J Environ Res Public Health* 2021; **18:** 4430.
	Haemaphysalis longicornis	Madison-Antenucci S, Kramer LD, Gebhardt LL, Kauffman E. Emerging tick-borne diseases. *Clin Microbiol Rev* 2020; **33:** e00083-18.
	Haemaphysalis punctata	Raad M, Azar D, Perotti MA. First report of the ticks *Haemaphysalis punctata* Canestrini et Fanzago, 1878, *Haemaphysalis parva* (Neumann, 1897) and *Dermacentor marginatus* (Sulzer, 1776) (Acari, Amblyommidae) from humans in Lebanon. *Acta Parasitol* 2020; **65:** 541-5.
	Haemaphysalis juxtakochi	Valente JDM, Silva PW, Arzua M, et al. Records of ticks (Acari: Ixodidae) on humans and distribution of spotted-fever cases and its tick vectors in Paraná State, southern Brazil. *Ticks Tick Borne Dis* 2020; **11:** 101510.
	Haemaphysalis concinna	Khasnatinov MA, Liapunov AV, Manzarova EL, Kulakova NV, Petrova IV, Danchinova GA. The diversity and prevalence of hard ticks attacking human hosts in Eastern Siberia (Russian Federation) with first description of invasion of non-endemic tick species. *Parasitol Res* 2016; **115:** 501-10.
	Haemaphysalis erinacei	Keskin A, Keskin A, Bursali A, Tekin S. Ticks (Acari: Ixodida) parasitizing humans in Corum and Yozgat provinces, Turkey. *Exp Appl Acarol* 2015; **67:** 607-16.
	Haemaphysalis parva	Keskin A, Keskin A, Bursali A, Tekin S. Ticks (Acari: Ixodida) parasitizing humans in Corum and Yozgat provinces, Turkey. *Exp Appl Acarol* 2015; **67:** 607-16.
	Haemaphysalis sulcata	Keskin A, Keskin A, Bursali A, Tekin S. Ticks (Acari: Ixodida) parasitizing humans in Corum and Yozgat provinces, Turkey. *Exp Appl Acarol* 2015; **67:** 607-16.
	Haemaphysalis flava	Ozawa A, Yamaguchi N, Hayakawa K, Matsuo I, Niizuma K, Ohkido M. [A case of tick bite (*Haemaphysalis flava*)--consideration of tularemia infection through tick bite]. *Nihon Hifuka Gakkai Zasshi* 1982; **92:** 1415-21.
	Haemaphysalis japonica	Sasaki K, Honma M, Nakao M, et al. Survey to detect tick-borne encephalitis virus from human-feeding ticks in Hokkaido, Japan. *J Dermatol* 2021; **48:** 1094-7.
	Haemaphysalis leachi	Dick G, Lewis E. A rickettsial disease in east Africa transmitted by ticks (*Rhipicephalus simus* and *Haemaphysalis leachi*). *T ROY SOC TROP MED H* 1947; **41:** 295-326.

Vector	Species	Reference
Tick	*Haemaphysalis leporispalustris*	Hahn MB, Disler G, Durden LA, et al. Establishing a baseline for tick surveillance in Alaska: tick collection records from 1909-2019. *Ticks Tick Borne Dis* 2020; **11:** 101495.
	Haemaphysalis megaspinosa	Seishima M, Izumi T, Oyama Z, et al. Tick bite by *Haemaphysalis megaspinosa* - first case. *Eur J Dermatol* 2000; **10:** 389-91.
	Hyalomma aegyptium	Keskin A, Keskin A, Bursali A, Tekin S. Ticks (Acari: Ixodida) parasitizing humans in Corum and Yozgat provinces, Turkey. *Exp Appl Acarol* 2015; **67:** 607-16.
	Hyalomma excavatum	Keskin A, Keskin A, Bursali A, Tekin S. Ticks (Acari: Ixodida) parasitizing humans in Corum and Yozgat provinces, Turkey. *Exp Appl Acarol* 2015; **67:** 607-16.
	Hyalomma marginatum	Keskin A, Keskin A, Bursali A, Tekin S. Ticks (Acari: Ixodida) parasitizing humans in Corum and Yozgat provinces, Turkey. *Exp Appl Acarol* 2015; **67:** 607-16.
	Hyalomma lusitanicum	Santos-Silva MM, Beati L, Santos AS, et al. The hard-tick fauna of mainland Portugal (Acari: Ixodidae): an update on geographical distribution and known associations with hosts and pathogens. *Exp Appl Acarol 2011*; **55:** 85-121.
	Hyalomma truncatum	Horak IG, Fourie LJ, Heyne H, Walker JB, Needham G. *Rhipicephalus* Ixodid ticks feeding on humans in South Africa: with notes on preferred hosts, geographic distribution, seasonal occurrence and transmission of pathogens. *Exp Appl Acarol* 2002; **27:** 113-36.
	Hyalomma anatolicum	Hosseini A, Dalimi A, Abdigoudarzi M. Morphometric study on male specimens of *Hyalomma anatolicum* (Acari: Ixodidae) in West of Iran. *Iran J Arthropod Borne Dis* 2011; **5:** 23-31.
	Hyalomma Asiaticum	Liu ZQ, Xia J, Wang GL, Kuermanali N. Cloning and expression of the 4D8 gene from *Hyalomma asiaticum* tick. *Genet Mol Res* 2016; **15**.
	Hyalomma detritum	Aktas M. A survey of ixodid tick species and molecular identification of tick-borne pathogens. *Vet Parasitol* 2014; **200:** 276-83.
	Hyalomma dromedarii	Mosabah AA, Morsy TA. Tick paralysis: first zoonosis record in Egypt. *J Egypt Soc Parasitol* 2012; **42:** 71-8.
	Hyalomma rufipes	Medialdea-Carrera R, Melillo T, Micaleff C, Borg ML. Detection of *Hyalomma rufipes* in a recently arrived asylum seeker to the EU. *Ticks Tick Borne Dis* 2021; **12:** 101571.
	Hyalomma scupense	Kar S, Dervis E, Akın A, Ergonul O, Gargili A. Preferences of different tick species for human hosts in Turkey. *Exp Appl Acarol* 2013; **61:** 349-55.
	Hyalomma impeltatum	Bursali A, Keskin A, Tekin S. Ticks (Acari: Ixodida) infesting humans in the provinces of Kelkit Valley, a Crimean-Congo Hemorrhagic Fever endemic region in Turkey. *Exp Appl Acarol* 2013; **59:** 507-15.
	Ixodes persulcatus	Madison-Antenucci S, Kramer LD, Gebhardt LL, Kauffman E. Emerging tick-borne diseases. *Clin Microbiol Rev* 2020; **33:** e00083-18.
	Ixodes ricinus	Madison-Antenucci S, Kramer LD, Gebhardt LL, Kauffman E. Emerging tick-borne diseases. *Clin Microbiol Rev* 2020; **33:** e00083-18.
	Ixodes scapularis	Madison-Antenucci S, Kramer LD, Gebhardt LL, Kauffman E. Emerging tick-borne diseases. *Clin Microbiol Rev* 2020; **33:** e00083-18.
	Ixodes holocyclus	Graves SR, Stenos J. Tick-borne infectious diseases in Australia. *Med J Aust* 2017; **206:** 320-4.
	Ixodes ovatus	Sasaki K, Honma M, Nakao M, et al. Survey to detect tick-borne encephalitis virus from human-feeding ticks in Hokkaido, Japan. *J Dermatol* 2021; **48:** 1094-7.
	Ixodes arboricola	Špitalská E, Boldišová E, Štefanidesová K, et al. Pathogenic microorganisms in ticks removed from Slovakian residents over the years 2008-2018. *Ticks Tick Borne Dis* 2021; **12:** 101626.
	Ixodes frontalis	Gilot B, Beaucournu JC, Chastel C. [Collecting with the flagging method and fixing on man of Ixodes (Trichotoixodes) frontalis (Panzer, 1795)]. *Parasite* 1997; **4:** 197-9.
	Ixodes hexagonus	Faulde MK, Rutenfranz M, Hepke J, Rogge M, Görner A, Keth A. Human tick infestation pattern, tick-bite rate, and associated *Borrelia burgdorferi* s.l. infection risk during occupational tick exposure at the Seedorf military training area, northwestern Germany. *Ticks Tick Borne Dis* 2014; **5:** 594-9.
	Ixodes nipponensis	Lee SH, Shin NR, Kim CM, et al. First identification of *Anaplasma phagocytophilum* in both a biting tick *Ixodes nipponensis* and a patient in Korea: a case report. *BMC Infect Dis* 2020; **20:** 826.
	Ixodes redikorzevi	Kassis I, Ioffe-Uspensky I, Uspensky I, Mumcuoglu KY. Human toxicosis caused by the tick *Ixodes redikorzevi* in Israel. *Isr J Med Sci* 1997; **33:** 760-1.
	Ixodes trianguliceps	Hubbard MJ, Baker AS, Cann KJ. Distribution of *Borrelia burgdorferi* s.l. spirochaete DNA in British ticks (Argasidae and Ixodidae) since the 19th century, assessed by PCR. *Med Vet Entomol* 1998; **12:** 89-97.
	Ixodes ventalloi	Sanogo YO, Parola P, Shpynov S, et al. Genetic diversity of bacterial agents detected in ticks removed from asymptomatic patients in northeastern Italy. *Ann N Y Acad Sci* 2003; **990:** 182-90.
	Rhipicephalus sanguineus	Madison-Antenucci S, Kramer LD, Gebhardt LL, Kauffman E. Emerging tick-borne diseases. *Clin Microbiol Rev* 2020; **33:** e00083-18.

Vector	Species	Reference
Tick	*Rhipicephalus microplus*	Szabó MPJ, Martins TF, Barbieri ARM, et al. Ticks biting humans in the Brazilian savannah: attachment sites and exposure risk in relation to species, life stage and season. *Ticks Tick Borne Dis* 2020; **11:** 101328.
	Rhipicephalus annulatus	Keskin A, Keskin A, Bursali A, Tekin S. Ticks (Acari: Ixodida) parasitizing humans in Corum and Yozgat provinces, Turkey. *Exp Appl Acarol* 2015; **67:** 607-16.
	Rhipicephalus bursa	Keskin A, Keskin A, Bursali A, Tekin S. Ticks (Acari: Ixodida) parasitizing humans in Corum and Yozgat provinces, Turkey. *Exp Appl Acarol* 2015; **67:** 607-16.
	Rhipicephalus turanicus	Keskin A, Keskin A, Bursali A, Tekin S. Ticks (Acari: Ixodida) parasitizing humans in Corum and Yozgat provinces, Turkey. *Exp Appl Acarol* 2015; **67:** 607-16.
	Rhipicephalus pusillus	Santos-Silva MM, Beati L, Santos AS, et al. The hard-tick fauna of mainland Portugal (Acari: Ixodidae): an update on geographical distribution and known associations with hosts and pathogens. *Exp Appl Acarol* 2011; **55:** 85-121.
	Rhipicephalus simus	Dick G, Lewis E. A rickettsial disease in East Africa transmitted by ticks (*Rhipicephalus simus* and *Haemaphysalis leachi*). *T Roy Soc Trop Med H* 1947; **41:** 295-326.
	Rhipicephalus appendiculatus	Horak IG, Fourie LJ, Heyne H, Walker JB, Needham G. *Rhipicephalus* Ixodid ticks feeding on humans in South Africa: with notes on preferred hosts, geographic distribution, seasonal occurrence and transmission of pathogens. *Exp Appl Acarol* 2002; **27:** 113-36.
	Rhipicephalus evertsi	Horak IG, Fourie LJ, Heyne H, Walker JB, Needham G. *Rhipicephalus* Ixodid ticks feeding on humans in South Africa: with notes on preferred hosts, geographic distribution, seasonal occurrence and transmission of pathogens. *Exp Appl Acarol* 2002; **27:** 113-36.
	Rhipicephalus haemaphysaloides	Soundararajan C, Nagarajan K, Arul Prakash M. Tick infestation in human beings in the Nilgiris and Kancheepuram district of Tamil Nadu, India. *J Parasit Dis* 2018; **42:** 50-4.
	Argas persicus	Dehhaghi M, Kazemi Shariat Panahi H, Holmes EC, Hudson BJ, Schloeffel R, Guillemin GJ. Human tick-borne diseases in Australia. *Front Cell Infect Microbiol* 2019; **9:** 3.
	Argas vespertilionis	Socolovschi C, Kernif T, Raoult D, Parola P. *Borrelia*, *Rickettsia*, and *Ehrlichia* species in bat ticks, France, 2010. *Emerg Infect Dis* 2012; **18:** 1966-75.
	Argas japonicus	Yan P, Qiu Z, Zhang T, et al. Microbial diversity in the tick *Argas japonicus* (Acari: Argasidae) with a focus on *Rickettsia* pathogens. *Med Vet Entomol* 2019; **33:** 327-35.
	Ornithodoros capensis	Dehhaghi M, Kazemi Shariat Panahi H, Holmes EC, Hudson BJ, Schloeffel R, Guillemin GJ. Human tick-borne diseases in Australia. *Front Cell Infect Microbiol* 2019; **9:** 3.
Mosquito	*Aedes albopictus*	Abduljalil JM, Abd Al Galil FM. Molecular pathogenesis of dengue virus infection in Aedes mosquitoes. *J Insect Physiol* 2022; **138:** 104367.
	Aedes luteocephalus	Abílio AP, Kampango A, Armando EJ, et al. First confirmed occurrence of the yellow fever virus and dengue virus vector *Aedes* (Stegomyia) *luteocephalus* (Newstead, 1907) in Mozambique. *Parasites Vectors* 2020; **13:** 350.
	Aedes vexans	Outammassine A, Zouhair S, Loqman S. Global potential distribution of three underappreciated arboviruses vectors (*Aedes japonicus*, *Aedes vexans* and *Aedes vittatus*) under current and future climate conditions. *Transbound Emerg Dis* 2021; **69:** e1160-e1171.
	Anopheles arabiensis	Mbewe RB, Keven JB, Mzilahowa T, et al. Blood-feeding patterns of *Anopheles* vectors of human malaria in Malawi: implications for malaria transmission and effectiveness of LLIN interventions. *Malar J* 2022; **21:** 67.
	Anopheles funestus	Mbewe RB, Keven JB, Mzilahowa T, et al. Blood-feeding patterns of *Anopheles* vectors of human malaria in Malawi: implications for malaria transmission and effectiveness of LLIN interventions. *Malar J* 2022; **21:** 67.
	Anopheles gambiae	Mbewe RB, Keven JB, Mzilahowa T, et al. Blood-feeding patterns of *Anopheles* vectors of human malaria in Malawi: implications for malaria transmission and effectiveness of LLIN interventions. *Malar J* 2022; **21:** 67.
	Anopheles pharoensis	Yamany AS, Abdel-Ghaffar F, Al Quraishy S, Al-Amri O, Mehlhorn H, Abdel-Gaber. Histological technique to detect the physiological age of the malaria vector mosquito *Anopheles pharoensis* (Diptera: Culicidae). *Microsc Res Techniq* 2022; **85:** 1580-87.
	Anopheles punctipennis	Dieme C, Ngo KA, Tyler S, et al. Role of *Anopheles* mosquitoes in Cache Valley Virus Lineage Displacement, New York, USA. *Emerg Infect Dis* 2022; **28:** 303-13.
	Anopheles sinensis	Luo DY, Yan ZT, Che LR, Zhu JJ, Chen B. Repellency and insecticidal activity of seven Mugwort (*Artemisia argyi*) essential oils against the malaria vector *Anopheles sinensis*. *Sci Rep* 2022; **12:** 5337.
	Anopheles ziemanni	Amvongo-Adjia N, Wirsiy EL, Riveron JM, et al. Bionomics and vectorial role of anophelines in wetlands along the volcanic chain of Cameroon. *Parasites Vectors* 2018; **11:** 471.

Vector	Species	Reference
Mosquito	*Armigeres subalbatus*	Muslim A, Fong MY, Mahmud R, Sivanandam S. Vector and reservoir host of a case of human *Brugia pahangi* infection in Selangor, peninsular Malaysia. *Trop Biomed* 2013; **30:** 727-30.
	Culex pipiens pallens	Zhang R, Liu W, Zhang Q, Zhang X, Zhang Z. Microbiota and transcriptome changes of *Culex pipiens pallens* larvae exposed to *Bacillus thuringiensis israelensis*. *Sci Rep* 2021; **11:** 20241.
	Culex tritaeniorhynchus	Van den Eynde C, Sohier C, Matthijs S, De Regge N. Japanese encephalitis virus interaction with mosquitoes: a review of vector competence, vector capacity and mosquito immunity. *Pathogens* 2022; **11:** 317.
	Mansonia uniformis	Krishnan J, Mathiarasan L. Prevalence of disease vectors in Lakshadweep Islands during post-monsoon season. *J Vector Borne* 2018; **55:** 189-96.
Flea	*Archaeopsylla erinacei*	Bork K, Honomichl K, Hoede N. [Flea bites caused by *Archaeopsylla erinacei*, the hedgehog flea]. *Hautarzt* 1987; **38:** 690-2.
	Ctenocephalides canis	Beck W, Clark HH. [Differential diagnosis of medically relevant flea species and their significance in dermatology]. *Hautarzt* 1997; **48:** 714-9.
	Ctenocephalides felis	Mumcuoglu Y, Rufli T. [Siphonaptera/fleas (author's transl)]. *Schweiz Rundsch Med Prax* 1979; **68:** 1172-82.
	Echidnophaga gallinacea	Salgado F, Elston DM. What's eating you? sticktight flea revisited. *Cutis* 2017; **100:** 9.
	Pulex irritans	Brouqui P, Raoult D. Arthropod-borne diseases in homeless. *Ann N Y Acad Sci* 2006; **1078:** 223-35.
	Xenopsylla cheopis	Brouqui P, Raoult D. Arthropod-borne diseases in homeless. *Ann N Y Acad Sci* 2006; **1078:** 223-35.
Mite	*Laelaps jettmari*	Andrew Rhipicephalus Epidemic haemorrhagic fever; 40 cases from Korea. *BMJ* 1953; **1:** 1063-8.
	Ornithonyssus bacoti	Beck W, Pfister K. [Mites as a cause of zoonoses in human beings]. *Wien Klin Wochenschr* 2006; **118:** 27-32.
Ked	*Lipoptena cervi*	Rantanen T, Reunala T, Vuojolahti P, Hackman W. Persistent pruritic papules from deer ked bites. *Acta Derm Venereol* 1982; **62:** 307-11.
	Lipoptena fortisetosa	Andreani A, Rosi MC, Guidi R, et al. Colour preference of the deer ked *Lipoptena fortisetosa* (Diptera: Hippoboscidae). *Insects* 2021; **12:** 845.
Louse	*pediculosis capitis*	Coates SJ, Thomas C, Chosidow O, Engelman D, Chang AY. Ectoparasites: Pediculosis and tungiasis. *J Am Acad Dermatol* 2020; **82:** 551-69.
Bug	*Cimex hemipterus*	Zahran Z, Ab Majid AH. Human skin reactions towards bites of tropical bed bug, *Cimex hemipterus* F. (Hemiptera: Cimicidae): a preliminary case study. *Asian Pac J Trop Biomed* 2016; **6:** 366-71.

Supplementary table 5: Original resolutions and extents of source datasets

Variable	Spatial resolution	Temporal extent	Source of data	Website	Reference
Climate data	0°2·5′	1980-2018*	WorldClim	https://www.worldclim.org/	Fick SE, Hijmans RJ. WorldClim 2: new 1-km spatial resolution climate surfaces for global land areas. *Int J Climatol* 2017; **37:** 4302-15.<br>Harris I, Jones PD, Osborn TJ, Lister DH. Updated high-resolution grids of monthly climatic observations – the CRU TS3.10 Dataset. *Int J Climatol* 2014; **34:** 623-42.
Leaf area index	8km	1981-2019	Resource and Environment Science and Data Center	https://www.resdc.cn/	Yang L, Liu R, Chen JM. Retrospective retrieval of long-term consistent global leaf area index (1981-2011) from combined AVHRR and MODIS data. *J Geophys Res Biogeosci*, 2015; 117.
Land cover	0·3km	1992-2019	European Space Agency	https://maps.elie.ucl.ac.be/CCI/	European Space Agency. ESA Land Cover Climate Change Initiative (Land_Cover_cci): Global Land Cover Maps, Version 2.0.7. https://catalogue.ceda.ac.uk/uuid/b382ebe6679d44b8b0e68ea4ef4b701c/ (accessed May 28, 2021)
Elevation	1km	2010	EarthEnv (DEM90)	http://www.earthenv.org/	Robinson N, Regetz J, Guralnick RP. EarthEnv-DEM90: A nearly-global, void-free, multi-scale smoothed, 90m digital elevation model from fused ASTER and SRTM data. *ISPRS* 2014; **87:** 57-67.
Livestock density	1km	2010	Food and Agriculture Organization (FAO)	http://www.fao.org/livestock-systems/en/	Gilbert M, Nicolas G, Cinardi G, et al. Global distribution data for cattle, buffaloes, horses, sheep, goats, pigs, chickens and ducks in 2010. *Sci Data* 2018; **5:** 180227.
Mammalian richness	0°0′30″	2013	International Union for Conservation of Nature (IUCN)	https://sedac.ciesin.columbia.edu/	International Union for Conservation of Nature - IUCN, and Center for International Earth Science Information Network - CIESIN - Columbia University. 2015. Gridded Species Distribution: Global Mammal Richness Grids, 2015 Release. Palisades, NY: NASA Socioeconomic Data and Applications Center (SEDAC).
Population number	1km	2020	WorldPop 2020	https://www.worldpop.org/	WorldPop. Population counts, unconstrained global mosaics 2000-2020 (1 km resolution), 2020. https://www.worldpop.org/geodata/listing?id=64/ (accessed Apr 12, 2021).

*This dataset is recalculated according to historical monthly weather data between 1980 to 2018, which is the main time period when the SFGR were detected, using the 'biovars' function in the R package dismo.

Supplementary table 6: Variables used for ecological modeling of SFGR in this study

Data	Variable	Description
Climate	BIO1	Annual mean temperature (°C)
	BIO2	Mean diurnal range (Mean of monthly (max temp-min temp)) (°C)
	BIO3	Isothermality (BIO2/ BIO7) (*100)
	BIO4	Temperature seasonality (standard deviation*100)
	BIO5	Max temperature of warmest month (°C)
	BIO6	Min temperature of coldest month (°C)
	BIO7	Annual range of temperature (BIO5- BIO6) (°C)
	BIO8	Mean temperature of wettest quarter (°C)
	BIO9	Mean temperature of driest quarter (°C)
	BIO10	Mean temperature of warmest quarter (°C)
	BIO11	Mean temperature of coldest quarter (°C)
	BIO12	Annual precipitation (mm)
	BIO13	Precipitation of wettest month (mm)
	BIO14	Precipitation of driest month (mm)
	BIO15	Precipitation seasonality (Coefficient of variation)
	BIO16	Precipitation of wettest quarter (mm)
	BIO17	Precipitation of driest quarter (mm)
	BIO18	Precipitation of warmest quarter (mm)
	BIO19	Precipitation of coldest quarter (mm)
Leaf area index	Leaf area index	area of leaves (m^2) over a unit of land (m^2)
Land cover	Cropland	Percentage coverage of cropland (%)
	Mixed cropland and nature vegetation	Percentage coverage of mixed cropland and nature vegetation (%)
	Forest	Percentage coverage of forest (%)
	Shrubland	Percentage coverage of shrubland (%)
	Mixed tree, shrub and herbaceous	Percentage coverage of mixed tree, shrub and herbaceous (%)
	Grassland	Percentage coverage of grassland (%)
	Lichens and mosses	Percentage coverage of lichens and mosses (%)
	Sparse vegetation land	Percentage coverage of sparse vegetation land (%)
	Flooded vegetation	Percentage coverage of flooded vegetation (%)
	Urban construction land	Percentage coverage of urban construction land (%)
	Bare areas	Percentage coverage of bare areas (%)
	Water body	Percentage coverage of inland water body (%)
	Ice and snow	Percentage coverage of ice and snow (%)
Elevation	Elevation	Average elevation (m)
Livestock	Buffalo	Density of buffalo (heads per km^2)
	Cattle	Density of cattle (heads per km^2)
	Goat	Density of goat (heads per km^2)
	Sheep	Density of sheep (heads per km^2)
	Horse	Density of horse (heads per km^2)
Mammals*	Mammalian richness	The number of mammal species per km^2

*This dataset was extracted from the NASA Socioeconomic Data and Applications Center (SEDAC) Gridded Species Distribution collection created from vector data files acquired from the International Union for Conservation of Nature (IUCN) Red List collection. The data represent the species of mammals at one kilometer resolution.

Supplementary table 7: The references for all the SFGR species

SFG rickettsiae species	Reference ID
R. aeschlimannii	12, 30, 40, 41, 50, 95, 137, 138, 142, 147, 148, 191, 193, 203, 204, 208, 288, 299, 311, 321, 331, 368, 373, 390, 397, 406, 441, 493, 494, 505, 522, 541, 571, 575, 579, 585, 593, 631, 633, 645, 660, 729, 732-738, 755, 783, 856, 877, 1040, 1042, 1046, 1047, 1049, 1051, 1055, 1077, 1096, 1104, 1106, 1108, 1118, 1120, 1122, 1144, 1273-1287, 1290-1295, 1300, 1304, 1311, 1377, 1413, 1500, 1515, 1518, 1536, 1560
R. africae	7, 9, 13-25, 40, 49, 58, 75, 83, 137, 156-158, 193, 194, 218, 220, 236, 251, 259, 266, 282, 284, 289, 290, 308, 309, 342, 347, 385, 387, 391, 395, 399, 423, 441, 447, 460, 494, 519-522, 534, 540, 541, 548, 549, 551, 568, 576, 593, 606, 625, 627, 629, 631, 633, 664, 697, 713, 714, 739-747, 802, 842, 856, 869, 906, 938, 971, 1040, 1045, 1048, 1055, 1064, 1073, 1087, 1101, 1102, 1104, 1108, 1113, 1130, 1132, 1144, 1151, 1165, 1277, 1281, 1286, 1287, 1294-1308, 1310-1312, 1383, 1444, 1535
R. akari	81, 210, 216, 340, 372, 380, 405, 418, 419, 431, 545, 824, 897-902, 944, 950, 953, 954, 957, 977, 985, 1030, 1061, 1128, 1187, 1191, 1194, 1260, 1261, 1541, 1549
R. amblyommii	3, 27, 51, 61, 74, 127, 136, 150, 151, 159, 161, 171, 180, 181, 200, 219, 229, 238, 261, 264, 315, 318, 319, 327, 349, 365, 403, 413, 507, 523, 535, 536, 539, 558, 596, 610, 616, 652, 691, 694, 696, 703, 705, 748, 751, 765, 824, 833, 859, 865, 872, 886, 912, 914, 947, 987, 991, 995, 1009, 1032, 1043, 1065, 1068, 1072, 1081, 1086, 1092, 1113, 1114, 1121, 1135, 1139, 1313-1356, 1360, 1451, 1458, 1509
R. asembonensis	67, 68, 118, 180, 261, 330, 345, 430, 514, 518, 526, 529, 546, 686, 878, 881, 888, 929, 1035, 1293, 1357-1365, 1428
R. australis	7, 194, 233, 273, 545, 669, 717-719, 1017, 1053, 1074, 1542
R. buchneri	327, 1497, 1506, 1543
R. conorii	5-8, 30, 33, 41, 46, 49, 57, 62, 79, 85, 93, 97-99, 103, 109, 117, 119, 124, 125, 128, 139, 152, 162, 170, 177, 188, 202-204, 209, 211, 216, 222-224, 226, 230-232, 236, 263, 265, 268, 271, 272, 280, 285, 290, 298, 301-304, 321, 322, 324, 334, 338, 346, 351, 355, 373, 376, 382, 385, 387, 394, 396, 398, 399, 401, 407-409, 416, 422, 429, 434-437, 439, 448, 449, 455, 461-474, 476-487, 489, 490, 495, 497, 500, 504, 524, 534, 547, 595, 599, 611, 612, 624, 654, 661, 663-665, 668, 670, 672, 673, 684, 697, 698, 716, 722-725, 728, 729, 752-756, 783, 802, 852, 856, 864, 880, 889, 891, 893, 895, 903, 905, 907, 925, 931, 934, 938-942, 944, 945, 951, 952, 956, 958, 959, 962-964, 968, 975-979, 986, 992, 997, 1000, 1006, 1008, 1012, 1018, 1023-1025, 1028, 1030, 1047, 1052, 1058, 1061, 1067, 1076, 1078, 1097, 1104, 1115, 1119, 1122, 1123, 1131, 1138, 1142, 1146-1148, 1173, 1187, 1191, 1194, 1235, 1260, 1261, 1264, 1291, 1299, 1304, 1366-1372, 1498, 1518, 1527, 1528, 1557-1559
R. cooleyi	3, 61, 264, 1114, 1492
R. endosymbiont of I. scapularis	536, 652, 1068, 1083, 1347, 1493, 1494, 1507
R. felis	2, 4, 27, 30, 52-56, 67, 80, 86, 92, 94, 96, 105, 116, 118, 130, 148, 160, 163-167, 170, 172, 173, 176, 177, 182, 192, 203, 205, 219, 222, 223, 225, 229, 235, 247, 260, 261, 287, 291-293, 295, 310, 314, 316, 317, 319, 322-324, 330, 335, 341, 343, 345, 356, 361, 369, 373, 380, 383, 384, 386, 400, 404, 410, 411, 415, 421, 453, 495, 506, 507, 510-513, 516-518, 525-532, 538, 545, 546, 548, 550, 553, 556, 558, 560, 562, 567, 568, 570, 572, 573, 577, 578, 580, 582-584, 586, 588-590, 595, 600, 618, 622, 630, 643, 644, 647, 648, 655, 658, 659, 675, 676, 683, 685-691, 731, 756-790, 854-856, 864, 866-868, 870, 871, 878, 881, 886-889, 892, 894, 895, 914, 925, 929, 939, 956, 965, 969, 985, 990, 991, 993, 995, 998, 1009, 1011, 1014, 1030, 1038, 1045, 1059, 1065, 1075, 1081, 1092, 1106, 1113, 1121, 1124, 1125, 1133, 1136, 1188, 1363, 1373-1378, 1380-1385, 1479, 1513, 1518, 1540, 1552, 1564
R. gravesii	590, 791, 1544
R. heilongjiangensis	10, 133, 142, 206, 331, 370, 389, 425, 432, 569, 589, 895, 1105, 1170, 1178, 1181, 1190, 1193, 1221, 1223-1225, 1228, 1233, 1237, 1245, 1250, 1258, 1259, 1262, 1267, 1362, 1386-1389, 1406, 1504, 1538, 1553
R. helvetica	1, 4, 28-30, 38, 42, 43, 48, 49, 66, 87, 95, 111-114, 120, 133, 135, 140, 149, 155, 168, 169, 183, 185, 189, 191, 195, 201, 203, 208, 223, 225, 242, 243, 252, 253, 281, 294, 296, 297, 300, 305, 312, 313, 321, 332, 343, 376, 379, 388, 392, 402, 412, 414, 424, 438, 439, 450, 454, 488, 543, 553-555, 561, 564, 581, 585, 589, 592, 594, 603, 619, 620, 632, 634, 636, 637, 649, 658, 681, 682, 692, 693, 695, 701, 704, 706, 707, 710, 720, 721, 756, 792-794, 855, 856, 860-864, 873-875, 889, 896, 908, 909, 924, 927, 932, 933, 946, 960, 961, 988, 996, 1007, 1021, 1022, 1025, 1034, 1038, 1041, 1047, 1050, 1051, 1062, 1070, 1071, 1075, 1082, 1084, 1091, 1093-1099, 1106, 1107, 1109-1112, 1116-1118, 1120, 1126, 1134, 1137, 1145, 1278, 1281, 1367, 1377, 1382, 1387, 1391-1418, 1420, 1472, 1496, 1498, 1505, 1513-1515, 1518, 1532
R. honei	31, 34, 36, 90, 213, 325, 350, 362, 641, 795, 853, 957, 963, 984, 1012, 1019
R. hoogstraalii	50, 321, 390, 574, 575, 1049, 1279, 1294, 1383, 1407, 1489-1491, 1510, 1545
R. japonica	77, 78, 84, 100, 102, 144, 177, 184, 196, 249, 286, 320, 324, 328, 353, 358, 364, 393, 442-446, 451, 545, 589, 604, 613, 650, 651, 798-801, 876, 904, 980, 981, 984, 1015, 1016, 1030, 1036, 1176, 1239, 1241, 1402, 1411, 1421, 1436, 1504, 1561, 1565
R. massiliae	30, 41, 48, 49, 63, 107, 137, 143, 148, 152, 188, 203, 204, 239, 290, 302, 311, 321, 326, 334, 385, 409, 423, 498, 515, 533, 534, 538, 547, 557, 559, 571, 574, 585, 593, 595, 631, 638, 641, 696, 729, 739, 755, 756, 760, 802, 838, 870, 871, 874, 875, 877, 925, 928, 1004, 1026, 1031, 1034, 1041, 1044, 1046, 1047, 1049, 1051, 1083, 1100, 1102, 1104, 1106, 1110, 1118, 1120, 1122, 1176, 1264, 1274, 1278, 1281, 1286, 1287, 1291, 1300, 1304, 1363, 1366, 1367, 1370, 1413, 1416, 1420, 1423-1428, 1486, 1515-1517, 1536, 1551

SFG rickettsiae species	Reference ID
R. monacensis	1, 38, 50, 60, 66, 111, 112, 114, 135, 143, 171, 183, 185, 195, 201, 203, 208, 238, 242, 297, 313, 344, 412, 414, 438, 450, 454, 460, 509, 515, 524, 543, 554, 555, 561, 564, 585, 588, 591, 592, 603, 619, 620, 632, 636, 637, 649, 658, 659, 681, 704, 706, 708, 802-804, 856, 860, 862, 873-875, 908, 924, 932, 933, 1022, 1026, 1034, 1051, 1055, 1062, 1084, 1091, 1093-1095, 1097-1099, 1109, 1112, 1116-1118, 1137, 1145, 1184, 1279, 1281, 1284, 1291, 1315, 1320, 1323, 1328, 1382, 1394, 1397, 1398, 1400, 1402-1405, 1407, 1411-1413, 1415-1418, 1420-1422, 1429-1436, 1496, 1500, 1511, 1515, 1518, 1529
R. montanensis	3, 51, 150, 205, 264, 413, 536, 539, 596, 667, 696, 705, 711, 812, 1043, 1114, 1135, 1352, 1437, 1502, 1550
R. parkeri	47, 61, 74, 121, 132, 146, 151, 172, 173, 198, 205, 212, 219, 227, 240, 257, 258, 264, 327, 330, 337, 349, 357, 374, 403, 433, 501, 536, 587, 596, 615, 617, 691, 696, 709, 765, 807-827, 829, 859, 872, 894, 914, 947, 987, 991, 995, 1004, 1009, 1010, 1013, 1027, 1032, 1033, 1043, 1065, 1068, 1081, 1085, 1088, 1114, 1121, 1139, 1277, 1292, 1309, 1317, 1320, 1323, 1330, 1335, 1338, 1344-1347, 1355, 1363, 1364, 1375, 1439-1451, 1453-1458, 1495, 1548
R. peacockii	61, 217, 327, 749, 1118, 1437, 1438, 1459, 1460, 1503
R. philipii	214, 326, 492, 730, 858, 1461, 1499, 1530
R. raoultii	38, 49, 50, 59, 114, 129, 133, 154, 183, 197, 203, 204, 206, 225, 239, 242, 243, 245, 311, 313, 324, 331, 343, 344, 366, 381, 389, 426, 427, 450, 454, 496, 499, 515, 524, 542, 554, 564, 566, 574, 585, 632, 636, 641, 659, 678, 680, 704, 732, 830, 831, 848, 856, 862, 863, 875, 895, 926, 961, 1034, 1038, 1041, 1042, 1044, 1050, 1051, 1056, 1077, 1091, 1095, 1099, 1102, 1105, 1111, 1118, 1136, 1137, 1144, 1278-1280, 1283, 1284, 1289, 1294, 1376, 1382, 1388, 1390, 1398, 1406, 1409, 1413, 1420, 1432, 1453, 1462-1474, 1484, 1512, 1520, 1523, 1554
R. rhipicephali	61, 74, 178, 205, 214, 217, 219, 264, 302, 326, 327, 334, 384, 385, 492, 535, 541, 596, 691, 730, 749, 765, 858, 914, 949, 991, 995, 1009, 1032, 1065, 1081, 1083, 1088, 1113, 1118, 1121, 1122, 1300, 1329, 1338, 1345, 1355, 1451, 1458, 1461, 1476, 1499, 1502, 1546
R. rickettsii	7, 26, 32, 35, 37, 45, 64, 65, 74, 91, 92, 101, 106, 108, 122, 126, 131, 136, 174, 178-180, 200, 205, 213, 216, 217, 219, 221, 227, 229, 238, 241, 250, 255, 262, 263, 267, 269, 270, 274, 275, 278-280, 283, 324, 326, 336, 339, 346, 354, 367, 375, 377, 403, 404, 413, 419, 428, 440, 452, 491, 502, 596, 598, 601, 602, 607, 614, 626, 631, 642, 656, 662, 667, 671, 691, 696, 704, 726, 730, 741, 765, 785, 789, 797, 824, 828, 833-836, 859, 865, 879, 881, 885, 894, 910-923, 937, 947-949, 951, 953, 954, 957, 966, 967, 970, 983, 985-987, 991, 995, 1001, 1004, 1009, 1010, 1013, 1032, 1033, 1039, 1054, 1057, 1059, 1065, 1066, 1072, 1081, 1086, 1088, 1092, 1103, 1127-1129, 1135, 1149, 1152, 1154-1158, 1160, 1163, 1164, 1212, 1309, 1323, 1335, 1338, 1345, 1360, 1363, 1379, 1439, 1451, 1453, 1461, 1477-1481, 1522, 1524-1526, 1533, 1540, 1556, 1563
R. sibirica	7, 30, 50, 82, 107, 115, 133, 137, 141, 143, 153, 175, 204, 244, 329, 331, 363, 371, 420, 425, 456-458, 493, 494, 496, 522, 534, 537, 569, 575, 608, 611, 731, 739, 805, 806, 837-841, 843-845, 924, 935, 944, 977, 1020, 1026, 1044, 1049, 1061, 1080, 1104-1106, 1167, 1169, 1170, 1172, 1175, 1185, 1187, 1191-1195, 1206, 1208, 1210, 1212, 1215, 1224-1226, 1228, 1235, 1238, 1249-1251, 1256, 1259-1262, 1267, 1270, 1274, 1279, 1282, 1286, 1288, 1406, 1466, 1521, 1523, 1533, 1534, 1558, 1562
R. slovaca	7, 30, 38, 41, 49, 50, 76, 95, 123, 138, 148, 203, 204, 226, 242, 243, 306, 307, 311, 313, 321, 334, 388, 390, 455, 499, 524, 553, 554, 563, 569, 574, 575, 585, 591, 621, 659, 678, 729, 754, 755, 792, 846-852, 863, 875, 877, 930, 961, 986, 994, 1029, 1034, 1038, 1044, 1050, 1051, 1077, 1089, 1090, 1096, 1106, 1115, 1118, 1278-1280, 1282, 1294, 1361, 1382, 1416, 1420, 1432, 1453, 1463, 1467, 1469-1471, 1475, 1482-1487, 1520, 1531, 1555
R. tamurae	641, 895, 1105, 1403, 1431, 1488, 1500, 1508, 1547
R. thailandii	1019
R. vini	924
Candidatus *R. andeanae*	27, 94, 151, 172, 173, 179, 198, 433, 536, 709, 731, 807-811, 814, 819, 1027, 1068, 1092, 1139, 1321, 1330, 1333, 1334, 1341, 1346, 1355, 1451
Candidatus *R. barbariae*	204, 321, 579, 739, 755, 877, 1049, 1278, 1285-1287, 1366, 1370, 1490, 1517, 1536
Candidatus *R. colombianensi*	179, 213
Candidatus *R. goldwasserii*	575, 1046, 1490
Candidatus *R. jingxinensis*	378, 566, 1283, 1388, 1431, 1501, 1519
Candidatus *R. kellyi*	69
Candidatus *R. longicornii*	537, 798
Candidatus *R. mendelii*	95
Candidatus *R. moyalensis*	1311
Candidatus *R. paranaensis*	1452, 1537
Candidatus *R. principis*	331, 1538, 1539
Candidatus *R. rara*	331
Candidatus *R. rioja*	49, 659, 1090, 1560
Candidatus *R. senegalensis*	2, 70, 118, 580, 583, 878, 1540
Candidatus *R. tarasevichiae*	71, 72, 133-135, 197, 206, 331, 332, 359, 674, 677, 710, 720, 1060, 1093, 1105, 1181, 1267, 1272, 1376, 1387, 1388, 1392, 1406, 1419, 1512, 1521, 1538
Candidatus *R. uralica*	332
Candidatus *R. xinyangensis*	73

Supplementary table 8: The co-infection of SFGR species and their infected vectors.

Rickettsiae 1	Rickettsiae 2	Detected vectors
R. africae	*R. aeschlimannii*	*Hyalomma marginatum*
R. africae	*R. massiliae*	*Rhipicephalus senegalensis*
R. amblyommii	*R. rickettsii*	*Amblyomma americanum*
R. conorii	*R. massiliae*	*Rhipicephalus sanguineus*
R. felis	*R. asembonensis*	*Ctenocephalides felis*
R. heilongjiangensis	*R. raoultii*	*Haemaphysalis concinna*
R. helvetica	*R. monacensis*	*Ixodes ventalloi*
R. helvetica	*R. slovaca*	*Dermacentor reticulatus*
R. montanensis	*R. rickettsii*	*Dermacentor variabilis*
Candidatus R. andeanae	*R. parkeri*	*Amblyomma maculatum*
Candidatus R. tarasevichiae	*R. heilongjiangensis*	*Haemaphysalis concinna*
Candidatus R. tarasevichiae	*R. raoultii*	*Dermacentor silvarum*, *Haemaphysalis concinna*

Supplementary table 9: The number of different infection types of pathogenic rickettsiae

Pathogenic rickettsiae	All infections	Confirmed infections by molecular assays	Serological positives[#]		Cases with reported clinical manifestations
			Paired serum samples	A single serum sample	
Overall	66 133 (100·0) *	19 734 (100·0)	3314 (100·0)	43 085 (100·0)	7477 (100) [§]
R. rickettsii	27 935 (42·2)	14 771 (74·9)	285 (8·6)	12 879 (29·9)	773 (10·3)
R. conorii	21 810 (33·0)	2397 (12·1)	2382 (71·9)	17 031 (39·5)	5347 (71·5)
R. sibirica	5549 (8·4)	279 (1·4)	1 (~0·0)	5269 (12·2)	97 (1·3)
R. felis	2084 (3·2)	796 (4·0)	353 (10·7)	935 (2·2)	39 (0·5)
R. japonica	2075 (3·1)	216 (1·1)	87 (2·6)	1772 (4·1)	276 (3·7)
R. heilongjiangensis	1463 (2·2)	122 (0·6)	98 (3·0)	1243 (2·9)	17 (0·2)
R. africae	1295 (2·0)	520 (2·6)	6 (0·2)	769 (1·8)	341 (4·6)
R. honei	1192 (1·8)	11 (0·1)		1181 (2·7)	9 (/)
R. akari	1095 (1·7)	24 (0·1)	65 (2·0)	1006 (2·3)	269 (3·6)
R. slovaca	458 (0·7)	247 (1·3)		211 (0·5)	77 (1·0)
R. helvetica	350 (0·5)	7 (~0·0)		343 (0·8)	13 (0·2)
Candidatus R. tarasevichiae	221 (0·3)	211 (1·1)		10 (~0·0)	61 (0·8)
R. parkeri	163 (0·2)	47 (0·2)		116 (0·3)	71 (0·9)
R. amblyommii	152 (0·2)		22 (0·7)	130 (0·3)	
R. australis	84 (0·1)	6 (~0·0)	12 (0·4)	66 (0·2)	48 (0·6)
R. rhipicephali	67 (0·1)			67 (0·2)	
R. raoultii	41 (0·1)	40 (0·2)		1 (~0·0)	30 (0·4)
R. massiliae	34 (0·1)	3 (~0·0)		31 (0·1)	3 (/)
R. aeschlimannii	28 (~0·0)	8 (~0·0)	2 (0·1)	18 (~0·0)	7 (/)
R. philipii	22 (~0·0)	18 (0·1)		4 (~0·0)	18 (/)
R. monacensis	7 (~0·0)	7 (~0·0)			5 (/)
Candidatus R. xinyangensis	6 (~0·0)	3 (~0·0)		3 (~0·0)	3 (/)
Candidatus R. kellyi	1 (~0·0))	1 (~0·0)			1 (/)
Candidatus R. rioja	1 (~0·0)		1 (~0·0)		

*Data are presented as numbers of positive infections and proportions (%). [§]Cases less than 10 are not included in the overall cases for they are not used to analyze the clinical manifestation. [#]Serological positives refer to those patients are detected positive by serological assays. Paired serum samples refer to cases with either a four-fold rise or a seroconversion when comparing acute and convalescent samples. A single serum sample refer to cases confirmed by a single positive serum sample.

Supplementary table 10: Clinical characteristics of human infections with different SFGR species in the world. Data are presented as numbers of positive cases and proportions (%). Only rickettsioses with ⩾10 cases are shown. Results are presented as intervals, where the lower bound is obtained assuming the frequency of each unreported symptom in each case series study was zero, and the upper bound is obtained assuming the frequency of each unreported symptom was the same as the minimum frequency among all reported symptoms in each case series study. Cells with a lower bound of the proportion ⩾30% are bolded.

**Symptoms (# of pathogens with lower bounds > 30%)**	**Disease (Pathogen, n=# of Cases)**							
	**Spotted fever group rickettsioses (SFGR, n=7477)**	***R. conorii* Infection* (*R. conorii*§, n=5347)**	**Rocky Mountain spotted fever (*R. rickettsii*, n=773)**	**African tick bite fever (*R. africae*, n=341)**	**Rickettsialpox (*R. akari*, n=269)**	**Japanese spotted fever (*R. japonica*, n=276)**	**Tick-borne lymphadenopathy (*R. slovaca*, n=77)**	***R. parkeri* rickettsiosis (*R. parkeri*, n=71)**
Skin symptoms								
Cutaneous Rash (8)	**6234–6361 (83·4–85·1)**	**4973–4975 (93·0–93·1)**	**403–514 (52·1–66·5)**	**150–155 (44·0–45·5)**	**267 (99·3)**	**214–217 (77·5–78·6)**	15 (19·5)	**54–55 (76·1–77·5)**
Eschar (10)	**3447–4120 (46·1–55·1)**	**2558–2922 (47·8–54·6)**	5–259 (0·6–33·5)	**291–292 (85·3–85·6)**	**238 (88·5)**	**138–181 (50·0–65·6)**	**50 (64·9)**	**59–60 (83·1–84·5)**
Influenza-like illness								
Fever (15)	**5591–5785 (74·8–77·4)**	**3778–3964 (70·7–74·1)**	**659–665 (85·3–86·0)**	**287 (84·2)**	**263 (97·8)**	**252–254 (91·3–92·0)**	**32 (41·6)**	**66 (93·0)**
Headache (11)	**3504–4293 (46·9–57·4)**	**2523–2989 (47·2–55·9)**	**378–517 (48·9–66·9)**	98–170 (28·7–49·9)	**211–238 (78·4–88·5)**	**84–150 (30·4–54·3)**	18–22 (23·4–28·6)	**48 (67·6)**
Fatigue (1)	1448–2790 (19·4–37·3)	1239–2049 (23·2–38·3)	80–336 (10·3–43·5)	47–129 (13·8–37·8)	4–48 (1·5–17·8)	20–120 (7·2–43·5)	**27–31 (35·1–40·3)**	8–11 (11·3–15·5)
Malaise (5)	261–1808 (3·5–24·2)	21–1060 (0·4–19·8)	8–262 (1·0–33·9)	1–90 (0·3–26·4)	2–46 (0·7–17·1)	**102–170 (37·0–61·6)**	1–12 (1·3–15·6)	7–11 (9·9–15·5)
Chills (0)	233–1774 (3·1–23·7)	45–1078 (0·8–20·2)	6–264 (0·8–34·2)	47–129 (13·8–37·8)	3–47 (1·1–17·5)	76–146 (27·5–52·9)	0–11 (0·0–14·3)	12–15 (16·9–21·1)
Cough (1)	210–1762 (2·8–23·6)	103–1118 (1·9–20·9)	53–309 (6·9–40·0)	5–90 (1·5–26·4)	0–44 (0·0–16·4)	11–108 (4·0–39·1)	1–12 (1·3–15·6)	0–5 (0·0–7·0)
Dizziness (0)	45–1635 (0·6–21·9)	15–1056 (0·3–19·7)	1–259 (0·1–33·5)	1–90 (0·3–26·4)	0–44 (0·0–16·4)	3–104 (1·1–37·7)	0–11 (0·0–14·3)	2–6 (2·8–8·5)
Motor system symptoms								
Myalgia (10)	**3660–4203 (49·0–56·2)**	**2800–3038 (52·4–56·8)**	**399–526 (51·6–68·0)**	**184–199 (54·0–58·4)**	**88–115 (32·7–42·8)**	16–114 (5·8–41·3)	2–13 (2·6–16·9)	**50 (70·4)**
Arthralgia (2)	1682–2785 (22·5–37·2)	1345–2016 (25·2–37·7)	212–366 (27·4–47·3)	41–125 (12·0–36·7)	8–49 (3·0–18·2)	7–106 (2·5–38·4)	1–12 (1·3–15·6)	13–16 (18·3–22·5)
Gastrointestinal symptoms								
Nausea (1)	357–1876 (4·8–25·1)	108–1129 (2·0–21·1)	159–388 (20·6–50·2)	19–104 (5·6–30·5)	18–48 (6·7–17·8)	8–109 (2·9–39·5)	0–11 (0·0–14·3)	5–7 (7·0–9·9)
Vomit (1)	308–1823 (4·1–24·4)	186–1174 (3·5–22·0)	64–309 (8·3–40·0)	14–99 (4·1–29·0)	3–47 (1·1–17·5)	8–108 (2·9–39·1)	0–11 (0·0–14·3)	1–4 (1·4–5·6)
Diarrhea (0)	168–1711 (2·2–22·9)	64–1084 (1·2–20·3)	79–313 (10·2–40·5)	9–94 (2·6–27·6)	0–44 (0·0–16·4)	1–102 (0·4–37·0)	0–11 (0·0–14·3)	2–5 (2·8–7·0)
Anorexia (0)	127–1705 (1·7–22·8)	67–1101 (1·3–20·6)	1–259 (0·1–33·5)	16–99 (4·7–29)	1–45 (0·4–16·7)	11–112 (4·0–40·6)	0–11 (0·0–14·3)	1–6 (1·4–8·5)
Other symptoms								
Lymphadenopathy (5)	1158–2230 (15·5–29·8)	735–1428 (13·7–26·7)	36–277 (4·7–35·8)	**162–168 (47·5–49·3)**	39–66 (14·5–24·5)	8–106 (2·9–38·4)	**53 (68·8)**	14–15 (19·7–21·1)
Conjunctivitis (0)	396–1841 (5·3–24·6)	362–1259 (6·8–23·5)	21–277 (2·7–35·8)	4–91 (1·2–26·7)	2–46 (0·7–17·1)	0–102 (0·0–37·0)	1–12 (1·3–15·6)	0–5 (0·0–7·0)
Edema (0)	228–1706 (3·0–22·8)	91–1113 (1·7–20·8)	130–288 (16·8–37·3)	2–91 (0·6–26·7)	0–44 (0·0–16·4)	2–104 (0·7–37·7)	0–11 (0·0–14·3)	0–5 (0·0–7·0)

§*R. conorii* refers to "*R. conorii* complex", including *R. conorii* subsp. *conorii*, *R. conorii* subsp. *caspia*, *R. conorii* subsp. *indica*, and *R. conorii* subsp. *israelensis*. *Rickettsial diseases caused by "*R. conorii* complex" include Mediterranean spotted fever, Astrakhan fever, Indian tick typhus and Israeli spotted fever. Rickettsioses are not shown in the table when total number of cases is no more than 10 due to the deficient representativeness.

When calculating the frequency of each clinical feature, we divided publications into two groups, case reports and case series. A case report describes clinical features of a single patient in detail, for which it is reasonable to assume unmentioned symptoms as absent. In contrast, a case series study summarizes clinical characteristics of a group of confirmed patients, for which it is unclear if unmentioned symptoms are truly absent from the whole group or just rare, especially when the group size is large. For case series studies, we therefore made a conservative assumption that the frequency of an unreported symptom could vary from 0 to the minimum frequency of all reported symptoms. Consequently, we report a range for each symptom if relevant data involve case series. For the recording of clinical symptoms/signs, only patients with confirmed species of SFGR were used, while studies on serology in individuals were excluded due to potential cross-reactivity between SFGR.

Supplementary Table 10 (Continued)

**Symptoms (# of pathogens with lower bounds > 30%)**	**Disease (Pathogen, n=# of Cases)**							
	***Candidatus* R. tarasevichiae Infection (*Candidatus* R. tarasevichiae, n=61)**	***R. sibirica* Infection (*R. sibirica*, n=97)**	**Queensland tick typhus (*R. australis*, n=48)**	**Flea borne spotted fever (*R. felis*, n=39)**	**Scalp Eschar and Neck Lymphadenopathy After Tick Bite (*R. raoultii*, n=30)**	**Pacific Coast Tick Fever (*R. philipii*, n=18)**	**Far Eastern spotted fever (*R. heilongjiangensis* , n=17)**	***R. helvetica* Infection (*R. helvetica*, n=13)**
Skin symptoms								
Cutaneous Rash	2 (3·3)	**86–87 (88·7–89·7)**	**48 (100·0)**	7–9 (17·9–23·1)	6 (20·0)	4 (22·2)	4 (23·5)	1–3 (7·7–23·1)
Eschar	12 (19·7)	**36–39 (37·1–40·2)**	7–9 (14·6–18·8)	9–12 (23·1–30·8)	**9 (30·0)**	**18 (100·0)**	**17 (100·0)**	0–2 (0·0–15·4)
Influenza-like illness								
Fever	**20 (32·8)**	**90 (92·8)**	**45 (93·8)**	**38 (97·4)**	**26 (86·7)**	**14 (77·8)**	**10 (58·8)**	**11 (84·6)**
Headache	8 (13·1)	**58–71 (59·8–73·2)**	12–13 (25·0–27·1)	**23–24 (59·0–61·5)**	**9 (30·0)**	**14 (77·8)**	**9 (52·9)**	**11 (84·6)**
Fatigue	3–5 (4·9–8·2)	6–33 (6·2–34·0)	0–2 (0·0–4·2)	7–11 (17·9–28·2)	6–7 (20·0–23·3)	0–2 (0·0–11·1)	0–3 (0·0–17·6)	1–3 (7·7–23·1)
Malaise	**49–51 (80·3–83·6)**	**35–59 (36·1–60·8)**	1–3 (2·1–6·3)	2–7 (5·1–17·9)	**19–20 (63·3–66·7)**	3–5 (16·7–27·8)	**10 (58·8)**	0–2 (0·0–15·4)
Chills	6–8 (9·8–13·1)	**32–56 (33·0–57·7)**	2–3 (4·2–6·3)	1–6 (2·6–15·4)	2–3 (6·7–10)	0–2 (0·0–11·1)	1–4 (5·9–23·5)	0–2 (0·0–15·4)
Cough	14–16 (23·0–26·2)	5–29 (5·2–29·9)	2–4 (4·2–8·3)	**12–15 (30·8–38·5)**	4–5 (13·3–16·7)	0–2 (0·0–11·1)	0–3 (0·0–17·6)	0–2 (0·0–15·4)
Dizziness	8–10 (13·1–16·4)	14–38 (14·4–39·2)	0–2 (0·0–4·2)	1–6 (2·6–15·4)	0–2 (0·0–6·7)	0–2 (0·0–11·1)	0–3 (0·0–17·6)	0–2 (0·0–15·4)
Motor system symptoms								
Myalgia	**32–34 (52·5–55·7)**	**43–58 (44·3–59·8)**	9–10 (18·8–20·8)	**15–18 (38·5–46·2)**	**11–12 (36·7–40)**	3–5 (16·7–27·8)	0–3 (0·0–17·6)	**8 (61·5)**
Arthralgia	0–4 (0·0–6·6)	**30–53 (30·9–54·6)**	6–8 (12·5–16·7)	11–15 (28·2–38·5)	0–2 (0·0–6·7)	3–5 (16·7–27·8)	0–3 (0·0–17·6)	**5 (38·5)**
Gastrointestinal symptoms								
Nausea	3–5 (4·9–8·2)	15–39 (15·5–40·2)	3–5 (6·3–10·4)	9–13 (23·1–33·3)	**10–11 (33·3–36·7)**	0–2 (0·0–11·1)	0–3 (0·0–17·6)	0–2 (0·0–15·4)
Vomit	2–4 (3·3–6·6)	4–28 (4·1–28·9)	7–9 (14·6–18·8)	**15–18 (38·5–46·2)**	4–5 (13·3–16·7)	0–2 (0·0–11·1)	0–3 (0·0–17·6)	0–2 (0·0–15·4)
Diarrhea	0–4 (0·0–6·6)	1–28 (1·0–28·9)	4–6 (8·3–12·5)	5–9 (12·8–23·1)	3–4 (10·0–13·3)	0–2 (0·0–11·1)	0–3 (0·0–17·6)	0–2 (0·0–15·4)
Anorexia	3–5 (4·9–8·2)	25–49 (25·8–50·5)	0–2 (0·0–4·2)	0–5 (0·0–12·8)	2–4 (6·7–13·3)	0–2 (0·0–11·1)	0–3 (0·0–17·6)	0–2 (0·0–15·4)
Other symptoms								
Lymphadenopathy	18 (29·5)	**52 (53·6)**	6–7 (12·5–14·6)	1–4 (2·6–10·3)	**17 (56·7)**	**12 (66·7)**	5 (29·4)	0–2 (0·0–15·4)
Conjunctivitis	0–4 (0·0–6·6)	0–27 (0·0–27·8)	1–3 (2·1–6·3)	3–6 (7·7–15·4)	0–2 (0·0–6·7)	0–2 (0·0–11·1)	0–3 (0·0–17·6)	2 (15·4)
Edema	0–4 (0·0–6·6)	2–29 (2·1–29·9)	1–3 (2·1–6·3)	0–5 (0·0–12·8)	0–2 (0·0–6·7)	0–2 (0·0–11·1)	0–3 (0·0–17·6)	0–2 (0·0–15·4)

Supplementary table 11: BRT-model-estimated mean (95% percentiles) relative contributions of top factors (RC>3%) to the spatial distribution of 17 SFGR.

Variable	*R. aeschlimannii*	*R. africae*	*R. amblyommii*	*R. conorii*	*R. felis*	*R. heilongjiangensis*	*R. helvetica*	*R. japonica*	*R. massiliae*
Annual mean temperature	11·6 (8·7–14·6)	13·7 (11·5–15·5)	15·1 (12·8–18·1)	28·3 (26·0–30·4)	18·4 (16·5–20·4)	15·3 (10·3–21·2)	19·4 (15·7–24·3)	9·8 (4·7–14·8)	16·7 (11·8–21·3)
Mean diurnal range	7·5 (5·4–9·7)	8·7 (7·0–10·6)	8·1 (6·6–9·9)	10·5 (8·5–12·5)	9·8 (8·3–11·0)		14·0 (10·5–18·8)		6·4 (4·6–8·4)
Isothermality								7·2 (2·8–13·5)	
Annual precipitation	16·9 (11·8–21·5)	15·6 (14·0–17·8)	11·5 (8·8–15·0)	4·4 (3·5–5·3)	7·2 (5·9–9·0)	23·5 (15·8–31·8)	8·1 (5·8–10·6)	60·2 (52·1–70·1)	4·8 (3·1–6·4)
Precipitation seasonality		7·3 (5·8–8·8)	4·5 (3·5–5·6)	5·4 (4·1–6·8)	5·4 (4·4–6·4)		19·5 (15·3–23·5)		
Precipitation of warmest quarter	25·7 (22·0–29·1)								
Precipitation of coldest quarter	7·5 (4·3–12·2)								15·5 (11·3–20·7)
Cropland	18·7 (13·5–25·0)	7·3 (5·4–9·1)		24·8 (22·1–27·1)	17·2 (14·0–20·1)	4·5 (2·1–8·5)	12·4 (9·2–16·2)	8·3 (4·3–14·3)	20·7 (16·4–26·4)
Urban construction land								6·7 (2·9–11·7)	
Mixed cropland and nature vegetation			4·8 (3·7–6·0)		9·4 (7·3–12·4)	3·6 (2·4–5·2)	4·4 (2·8–7·5)		
Mixed tree, shrub and herbaceous				3·4 (2·5–4·6)			7·4 (5·7–9·4)		
Shrubland		3·4 (2·4–4·5)							
Grassland			4·2 (2·9–5·8)			3·6 (2·0–6·0)			
Leaf area index		9·4 (6·8–11·8)	12·3 (9·5–15·4)	7·0 (5·7–8·6)	4·6 (3·8–5·5)	8·6 (5·0–13·8)			9·8 (6·8–13·6)
Elevation	5·5 (4·0–7·9)		4·2 (3·2–5·3)	5·2 (3·9–6·7)	7·3 (5·8–8·7)	9·6 (6·4–12·8)			4·5 (2·8–6·1)
Horse		7·3 (5·2–9·3)	21·3 (16·7–25·4)		10·1 (8·3–12·3)		9·7 (5·4–14·2)		3·9 (2·6–5·4)
Sheep	6·5 (4·6–8·3)	15·7 (13·0–18·3)	4·3 (3·4–5·8)	4·2 (3·2–5·4)	4·8 (3·8–5·8)	13·5 (8·7–18·3)	5·0 (3·6–7·0)	7·3 (4·2–11·2)	12·0 (7·3–17·7)
Mammalian richness		11·7 (9·3–13·8)	9·7 (7·7–11·8)	6·8 (5·2–8·5)	5·8 (4·8–6·8)	17·8 (10·8–24·8)			5·5 (4·0–7·3)

Supplementary table 11 (Continued)

Variable	*R. monacensis*	*R. parkeri*	*R. raoultii*	*R. rhipicephali*	*R. rickettsii*	*R. sibirica*	*R. slovaca*	*Candidatus* R. tarasevichiae
Annual mean temperature	18·1 (14·5–21·5)	27·6 (24·2–31·0)	17·9 (15·6–20)	10·9 (6·7–15·0)	10·5 (8·8–12·4)	10·5 (8·7–12·3)	10·0 (7·1–13·6)	24·7 (19·1–31·6)
Mean diurnal range	14·5 (10·6–18·7)		5·0 (3·6–6·6)		6·3 (5·0–8·0)	5·1 (3·7–7·2)		
Annual precipitation	4·4 (2·9–6·4)	4·7 (3·3–6·1)			13·5 (9·5–19·7)	4·8 (2·9–6·6)	3·9 (2·5–5·4)	
Precipitation seasonality	9·5 (5·4–14·1)	5·8 (4·0–7·5)	5·8 (4·6–7·2)	24·9 (19·6–29·3)	4·8 (3·6–6·1)	15·5 (14·1–17·2)	22·0 (18·9–25·1)	
Precipitation of warmest quarter		3·9 (2·8–5·6)						
Precipitation of coldest quarter				28·7 (23·0–33·2)				10·4 (5·5–16·1)
Cropland	17·4 (11·9–23·1)		14·8 (10·8–18·8)		5·9 (4·1–8·0)	14·7 (10·6–18·5)	17·9 (10·9–25·8)	12·2 (7·2–18·0)
Mixed cropland and nature vegetation	12·4 (8·7–17·1)		6·9 (5·2–9·4)			4·9 (2·6–8·0)	19·2 (13·7–25·0)	7·6 (4·5–12)
Mixed tree, shrub and herbaceous								4·3 (2·0–6·9)
Shrubland								
Grassland		5·2 (3·6–7·0)		6·5 (2·5–10·9)				5·0 (2·6–9·5)
Leaf area index		5·7 (4·5–7·1)			7·7 (5·7–10·1)	6·1 (4·2–8·8)		21·6 (15·7–27·6)
Elevation	7·7 (5·8–9·3)	8·6 (6·4–10·3)	7·3 (5·5–8·9)		4·0 (3·1–5·1)	11·6 (9·3–13·7)	5·1 (3·1–7·8)	
Horse	5·6 (3·7–8·2)	26·8 (22·6–30·5)	17·7 (13·8–23·1)	11·3 (6·1–18·9)	14·8 (9·2–21·0)	14·4 (10·5–19·4)	5·9 (4·1–7·7)	
Sheep		4·4 (2·9–6·2)	5·4 (3·7–6·9)	5·1 (3·0–7·1)			12·1 (7·2–15·8)	4·9 (2·4–8·4)
Mammalian richness	10·5 (8·0–13·5)	7·3 (6·1–9·0)	19·1 (15·3–24·2)	12·7 (9·6–15·6)	32·6 (24·6–39·8)	12·4 (8·6–16·3)	4·0 (2·6–5·6)	9·2 (4·6–16·6)

Supplementary table 12: BRT and RF model-estimated mean (95% percentiles) relative contributions of top five factors to the spatial distribution of 17 SFGR.

Variable	Model	*R. aeschlimannii*	*R. africae*	*R. amblyommii*	*R. conorii*	*R. felis*	*R. heilongjiangensis*	*R. helvetica*	*R. japonica*	*R. massiliae*
Annual mean temperature	BRT	11·6 (8·7–14·6)	13·7 (11·5–15·5)	15·1 (12·8–18·1)	28·3 (26·0–30·4)	18·4 (16·5–20·4)	15·3 (10·3–21·2)	19·4 (15·7–24·3)	9·8 (4·7–14·8)	16·7 (11·8–21·3)
	RF		8·8 (7·9–10·0)	8·2 (7·4–9·7)	13·3 (9·3–18·2)	8·9 (8·0–11·4)	8·3 (6·6–9·7)	10·2 (8·4–13·4)	12·5 (9·9–15·2)	7·9 (6·8–10·0)
Mean diurnal range	BRT				10·5 (8·5–12·5)	9·8 (8·3–11·0)		14 (10·5–18·8)		
	RF	6·1 (4·9–8·3)			9·5 (7·3–12·1)	7·5 (6·7–8·4)		7·8 (6·4–9·9)	6·7 (5·0–8·6)	
Isothermality	BRT								7·7 (2·8–13·5)	
	RF									
Annual precipitation	BRT	16·9 (11·8–21·5)	15·6 (14·0–17·8)	11·5 (8·8–15·0)			23·5 (15·8–31·8)		60·2 (52·1–70·1)	
	RF	6·9 (6·1–7·7)	10·3 (8·4–13·1)	8·4 (7·7–9·4)			14·5 (9·7–18·7)	7·0 (6·0–7·7)	19·7 (12–30·2)	
Precipitation seasonality	BRT							19·5 (15·3–23·5)		
	RF							9·2 (7·6–12·4)		
Precipitation of warmest quarter	BRT	25·7 (22·0–29·1)								
	RF	11·2 (7·5–17·6)								
Precipitation of coldest quarter	BRT	7·5 (4·3–12·2)								15·5 (11·3–20·7)
	RF	7·0 (4·9–8·6)								9·7 (7·3–13·0)
Cropland	BRT	18·7 (13·5–25·0)			24·8 (22·1–27·1)	17·2 (14·0–20·1)		12·4 (9·2–16·2)	8·3 (4·3–14·3)	20·7 (16·4–26·4)
	RF	8·5 (6·7–11·7)			10·5 (7·5–17·7)	8·0 (6·9–12·3)		6·7 (5·9–8·5)		8·8 (6·5–12·0)
Mixed cropland and nature vegetation	BRT					9·4 (7·3–12·4)				
	RF									
Leaf area index	BRT		9·4 (6·8–11·8)	12·3 (9·5–15·4)	7·0 (5·7–8·6)					9·8 (6·8–13·6)
	RF		7·7 (6·7–9·1)	7·4 (6·8–8·6)	6·6 (5·8–7·4)					7·3 (6·3–8·8)
Elevation	BRT						9·6 (6·4–12·8)			
	RF					7·5 (6·7–8·4)	7·6 (6·3–9·3)			
Horse	BRT			21·3 (16·7–25·4)		10·1 (8·3–12·3)		9·7 (5·4–14·2)		
	RF			8·7 (7·6–11·8)		7·7 (7·0–9·1)				
Sheep	BRT		15·7 (13·0–18·3)				13·5 (8·7–18·3)		7·3 (4·2–11·2)	12·0 (7·3–17·7)
	RF		9·5 (7·7–13)				9·2 (7–12·4)		8·5 (6·5–10·4)	7·3 (6·0–9·2)
Mammalian richness	BRT		11·7 (9·3–13·8)	9·7 (7·7–11·8)	6·8 (5·2–8·5)		17·8 (10·8–24·8)			
	RF		9·1 (8·4–10·2)	8·0 (7·5–8·7)	6·7 (5·9–7·4)		11·0 (8·2–15·4)		7·4 (5·5–10·2)	

Supplementary table 12 (Continued)

Variable	Model	*R. monacensis*	*R. parkeri*	*R. raoultii*	*R. rhipicephali*	*R. rickettsii*	*R. sibirica*	*R. slovaca*	*Candidatus* R. tarasevichiae
Annual mean temperature	BRT	18·1 (14·5–21·5)	27·6 (24·2–31·0)	17·9 (15·6–20·0)	10·9 (6·7–15·0)	10·5 (8·8–12·4)		10·0 (7·1–13·6)	24·7 (19·1–31·6)
	RF	8·8 (7·0–10·2)	11 (7·9–14·7)	9·6 (8·2–12·4)	8·2 (5·7–9·5)	7·4 (6·5–7·9)	8·8 (7·7–10·9)	8·1 (6·1–10)	11·0 (8·6–13·8)
Mean diurnal range	BRT	14·5 (10·6–18·7)							
	RF	9·7 (7·4–12·8)				7·1 (6·2–8·0)			
Isothermality	BRT								
	RF								
Annual precipitation	BRT					13·5 (9·5–19·7)			
	RF					8·9 (7·8–10·6)			
Precipitation seasonality	BRT		5·8 (4·0–7·5)		24·9 (19·6–29·3)		15·5 (14·1–17·2)	22·0 (18·9–25·1)	
	RF	7·7 (5·7–10·1)		6·0 (4·8–6·7)	13·1 (8·8–19·4)		12·2 (9·2–15·1)	15·6 (10·1–21·3)	
Precipitation of warmest quarter	BRT								
	RF								
Precipitation of coldest quarter	BRT				28·7 (23–33·2)				10·4 (5·5–16·1)
	RF				16 (9·7–25·0)				7·0 (5·9–9·1)
Cropland	BRT	17·4 (11·9–23·1)		14·8 (10·8–18·8)			14·7 (10·6–18·5)	17·9 (10·9–25·8)	12·2 (7·2–18·0)
	RF			8·6 (6·9–10·7)			9·1 (6·6–12·6)	10·0 (7·5–15·1)	7·8 (6–9·8)
Mixed cropland and nature vegetation	BRT	12·4 (8·7–17·1)						19·2 (13·7–25·0)	
	RF							9·5 (6·6–13·9)	
Leaf area index	BRT					7·7 (5·7–10·1)			21·6 (15·7–27·6)
	RF		6·5 (5·4–7·4)						10·0 (7·5–13·6)
Elevation	BRT		8·6 (6·4–10·3)	7·3 (5·5–8·9)			11·6 (9·3–13·7)		
	RF	7·8 (6·1–10·4)	6·3 (5·3–7·4)				8·5 (6·7–10·9)		
Horse	BRT		26·8 (22·6–30·5)	17·7 (13·8–23·1)	11·3 (6·1–18·9)	14·8 (9·2–21·0)	14·4 (10·5–19·4)		
	RF		11·6 (8·1–14·8)	9·7 (7·9–11·9)	6·2 (4·4–8·0)	9·1 (7·7–11·4)	9·6 (7·0–12·5)		
Sheep	BRT							12·1 (7·2–15·8)	
	RF							9·9 (7·6–13·9)	
Mammalian richness	BRT	10·5 (8·0–13·5)	7·3 (6·1–9·0)	19·1 (15·3–24·2)	12·7 (9·6–15·6)	32·6 (24·6–39·8)	12·4 (8·6–16·3)		9·2 (4·6–16·6)
	RF	7·6 (5·8–8·8)	6·9 (5·8–7·7)	11·7 (9·0–16·7)	8·4 (6·7–10·4)	13·7 (9·9–20·0)			8·3 (6·7–11·6)

Supplementary table 13: BRT-model-estimated mean (95% percentiles) relative contributions of top factors (RC>3%) to five clusters. The greatest relative contribution for each cluster is bolded.

Variable	Cluster I	Cluster II	Cluster III	Cluster IV	Cluster V
Annual mean temperature	**22·1 (16·0–30·3)**	18·9 (9·7–29·7)	13·0 (6·1–20·1)	15·0 (8·1–20·6)	16·0 (8·3–30·0)
Mean diurnal range	14·0 (10·5–18·8)	8·1 (4·8–11·9)	8·7 (7·0–10·6)	8·6 (3·7–17·1)	7·2 (5·1–9·4)
Isothermality			7·7 (2·8–13·5)		
Annual precipitation	8·1 (5·8–10·6)	8·7 (3·3–20·7)	**33·1 (14·2–65·6)**	5·1 (2·7–8·2)	9·9 (3·5–17·2)
Precipitation seasonality	19·5 (15·3–23·5)	5·4 (4·1–6·8)	7·3 (5·8–8·8)	11·6 (4·7–23·5)	10·0 (3·7–28·3)
Precipitation of warmest quarter		**25·7 (22·0–29·1)**			3·9 (2·8–5·6)
Precipitation of coldest quarter	10·4 (5·5–16·1)	11·5 (4·7–20·1)			**28·7 (23·0–33·2)**
Cropland	12·3 (8·0–17·5)	21·4 (15·2–26·7)	6·7 (2·3–11·8)	**16·4 (11·1–23·1)**	5·9 (4·1–8·0)
Cropland and natural vegetation	6·0 (3·0–11·7)		3·6 (2·4–5·2)	10·5 (3·4–22·8)	4·8 (3·7–6·0)
Tree and shrub and herbaceous	5·9 (2·4–8·8)	3·4 (2·5–4·6)			
Shrub			3·4 (2·4–4·5)		
Grass	5·0 (2·6–9·5)		3·6 (2·0–6·0)		5·3 (2·9–9·9)
Urban construction land			6·7 (2·9–11·7)		
Leaf area index	21·6 (15·7–27·6)	8·4 (5·9–12·8)	9·0 (5·7–13·0)	5·4 (3·9–8·5)	8·6 (4·7–14·7)
Elevation		5·1 (3·2–7·0)	9·6 (6·4–12·8)	7·8 (3·9–12·9)	5·6 (3·2–10·0)
Horse	9·7 (5·4–14·2)	3·9 (2·6–5·4)	7·3 (5·2–9·3)	10·7 (4·4–21·3)	18·5 (7·1–29·3)
Sheep	5·0 (2·5–8·0)	7·6 (3·3–16·5)	12·2 (5·2–17·9)	7·4 (3·8–15·0)	4·6 (3·1–6·6)
Mammals	9·2 (4·6–16·6)	6·2 (4·1–8·4)	14·7 (9·6–24·3)	10·4 (3·0–21·8)	15·6 (6·4–37·3)

Supplementary figure 1: Correlation matrices of ecological variables used for screening multicollinearity before modeling the predominant 17 SFGR species. Heatmaps are based on Spearman correlation. Red and blue dots represent the positive and negative Spearman correlations respectively. The absolute value of the correlation coefficient is represented by the size of each dot. The exact values of correlations are shown in the lower triangle. The variable names marked in red represent those excluded from modeling to reduce multicolinearity. Roman numerals 1-17 correspond to the 17 predominant SFGR species respectively: (I) *R. aeschlimannii*; (II) *R. africae*; (III) *R. amblyommii*; (IV) *R. conorii*; (V) *R. felis*; (VI) *R. heilongjiangensis*; (VII) *R. helvetica*; (VIII) *R. japonica*; (IX) *R. massiliae*; (X) *R. monacensis*; (XI) *R. parkeri*; (XII) *R. raoultii*; (XIII) *R. rhipicephali*; (XIV) *R. rickettsii*; (XV) *R. sibirica*; (XVI) *R. slovaca*; (XVII) *Candidatus* R. tarasevichiae.

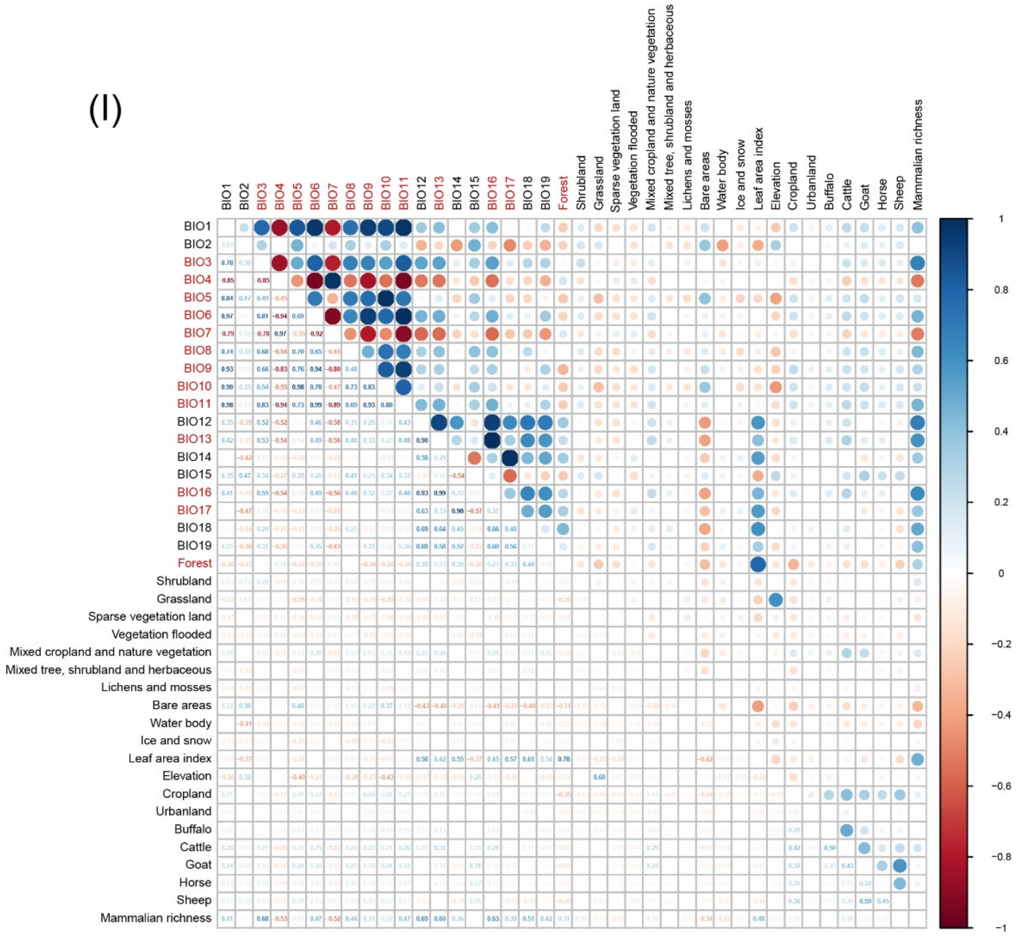

(II)

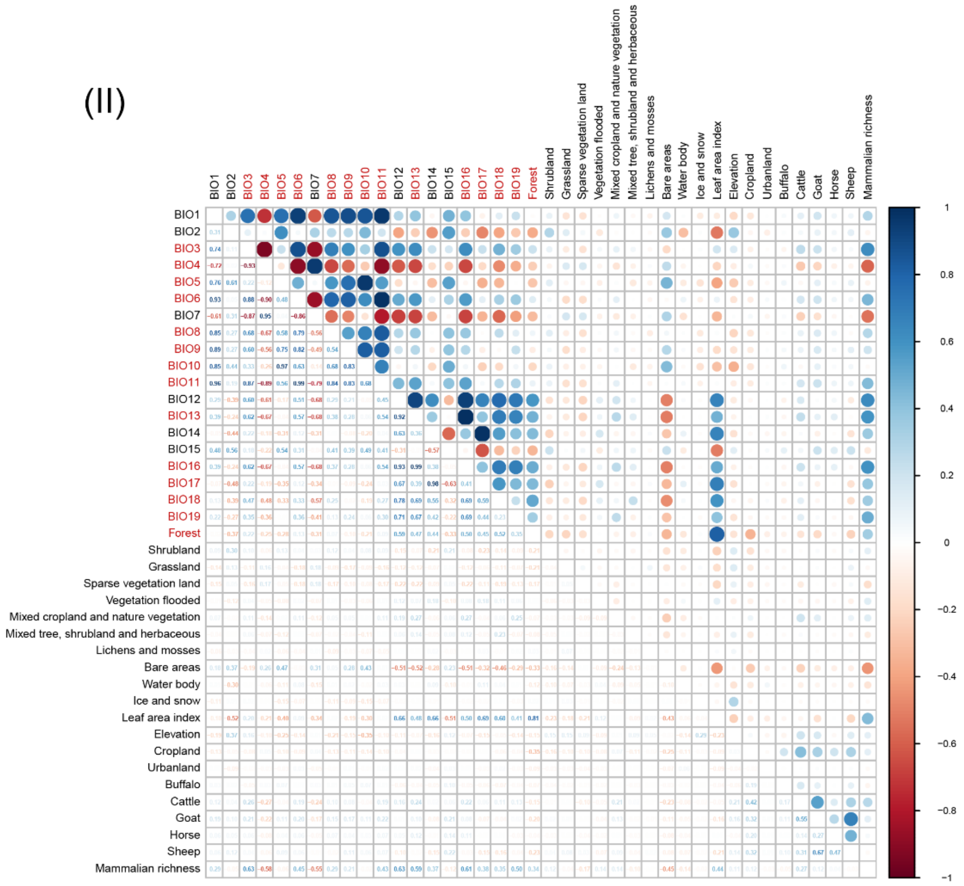
BIO1
BIO2
BIO3
BIO4
BIO5
BIO6
BIO7
BIO8
BIO9
BIO10
BIO11
BIO12
BIO13
BIO14
BIO15
BIO16
BIO17
BIO18
BIO19
Forest
Shrubland
Grassland
Sparse vegetation land
Vegetation flooded
Mixed cropland and nature vegetation
Mixed tree, shrubland and herbaceous
Lichens and mosses
Bare areas
Water body
Ice and snow
Leaf area index
Elevation
Cropland
Urbanland
Buffalo
Cattle
Goat
Horse
Sheep
Mammalian richness
1
0.8
0.6
0.4
0.2
0
−0.2
−0.4
−0.6
−0.8
−1

(III)

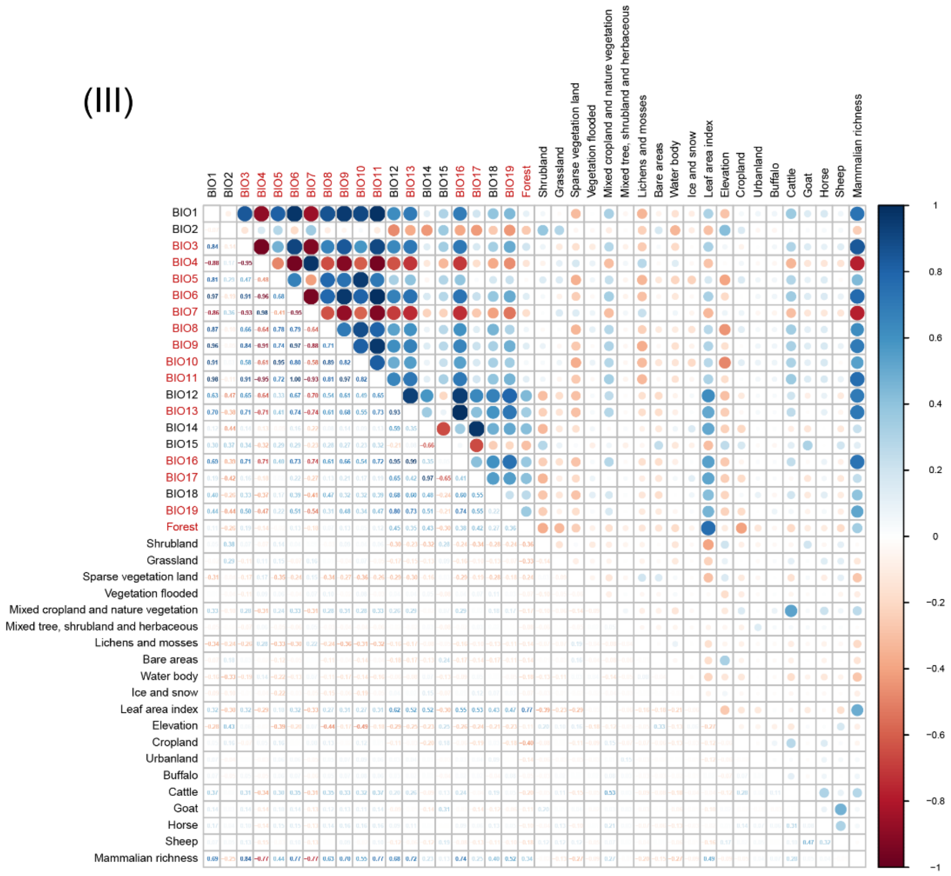
BIO1
BIO2
BIO3
BIO4
BIO5
BIO6
BIO7
BIO8
BIO9
BIO10
BIO11
BIO12
BIO13
BIO14
BIO15
BIO16
BIO17
BIO18
BIO19
Forest
Shrubland
Grassland
Sparse vegetation land
Vegetation flooded
Mixed cropland and nature vegetation
Mixed tree, shrubland and herbaceous
Lichens and mosses
Bare areas
Water body
Ice and snow
Leaf area index
Elevation
Cropland
Urbanland
Buffalo
Cattle
Goat
Horse
Sheep
Mammalian richness
1
0.8
0.6
0.4
0.2
0
−0.2
−0.4
−0.6
−0.8
−1

(IV)

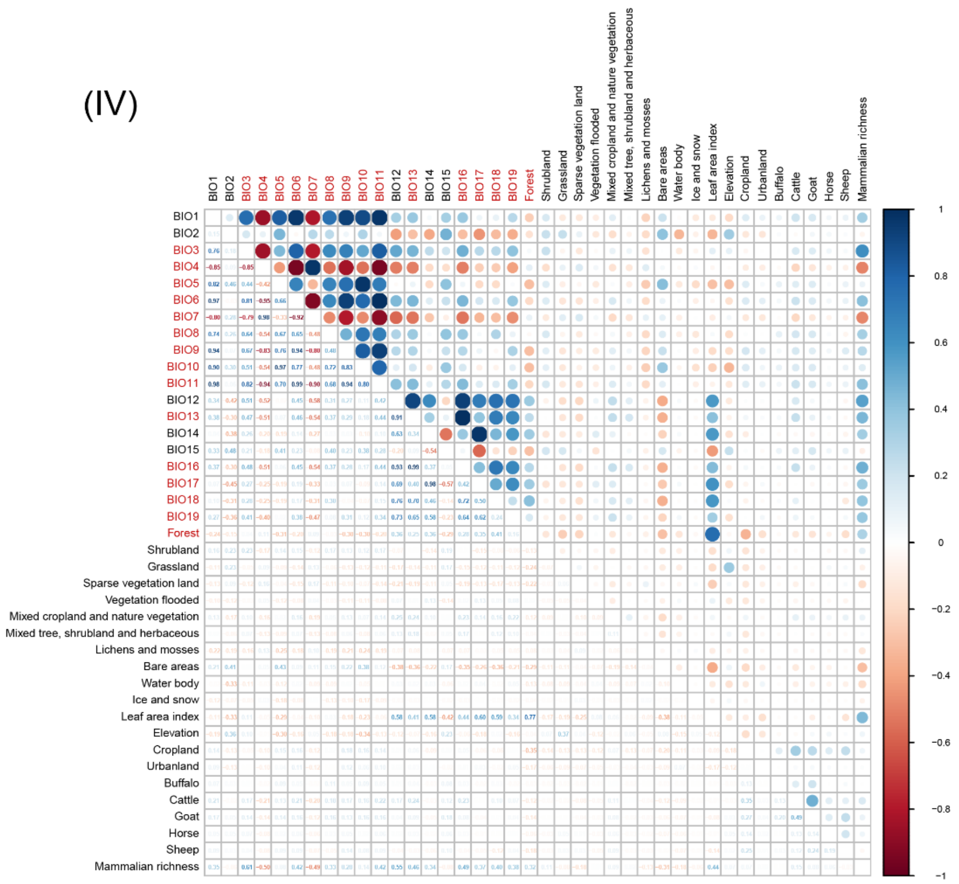
BIO1
BIO2
BIO3
BIO4
BIO5
BIO6
BIO7
BIO8
BIO9
BIO10
BIO11
BIO12
BIO13
BIO14
BIO15
BIO16
BIO17
BIO18
BIO19
Forest
Shrubland
Grassland
Sparse vegetation land
Vegetation flooded
Mixed cropland and nature vegetation
Mixed tree, shrubland and herbaceous
Lichens and mosses
Bare areas
Water body
Ice and snow
Leaf area index
Elevation
Cropland
Urbanland
Buffalo
Cattle
Goat
Horse
Sheep
Mammalian richness
1
0.8
0.6
0.4
0.2
0
−0.2
−0.4
−0.6
−0.8
−1

(V)

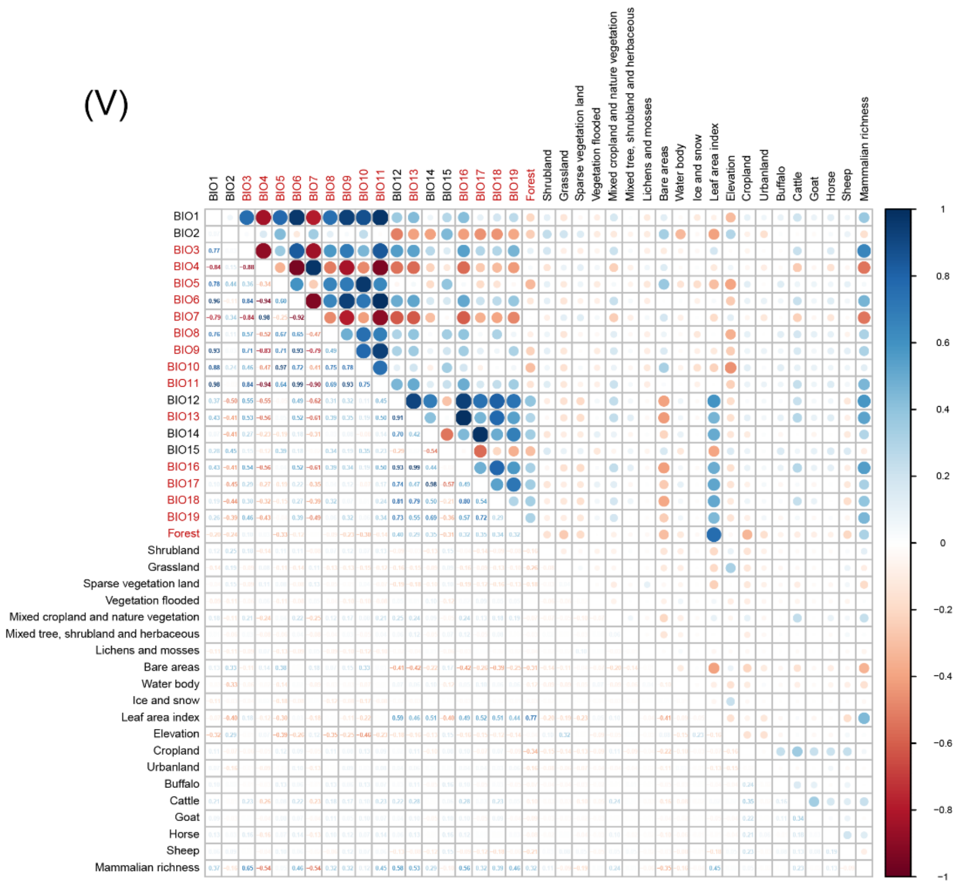
BIO1
BIO2
BIO3
BIO4
BIO5
BIO6
BIO7
BIO8
BIO9
BIO10
BIO11
BIO12
BIO13
BIO14
BIO15
BIO16
BIO17
BIO18
BIO19
Forest
Shrubland
Grassland
Sparse vegetation land
Vegetation flooded
Mixed cropland and nature vegetation
Mixed tree, shrubland and herbaceous
Lichens and mosses
Bare areas
Water body
Ice and snow
Leaf area index
Elevation
Cropland
Urbanland
Buffalo
Cattle
Goat
Horse
Sheep
Mammalian richness
1
0.8
0.6
0.4
0.2
0
−0.2
−0.4
−0.6
−0.8
−1

(VI)

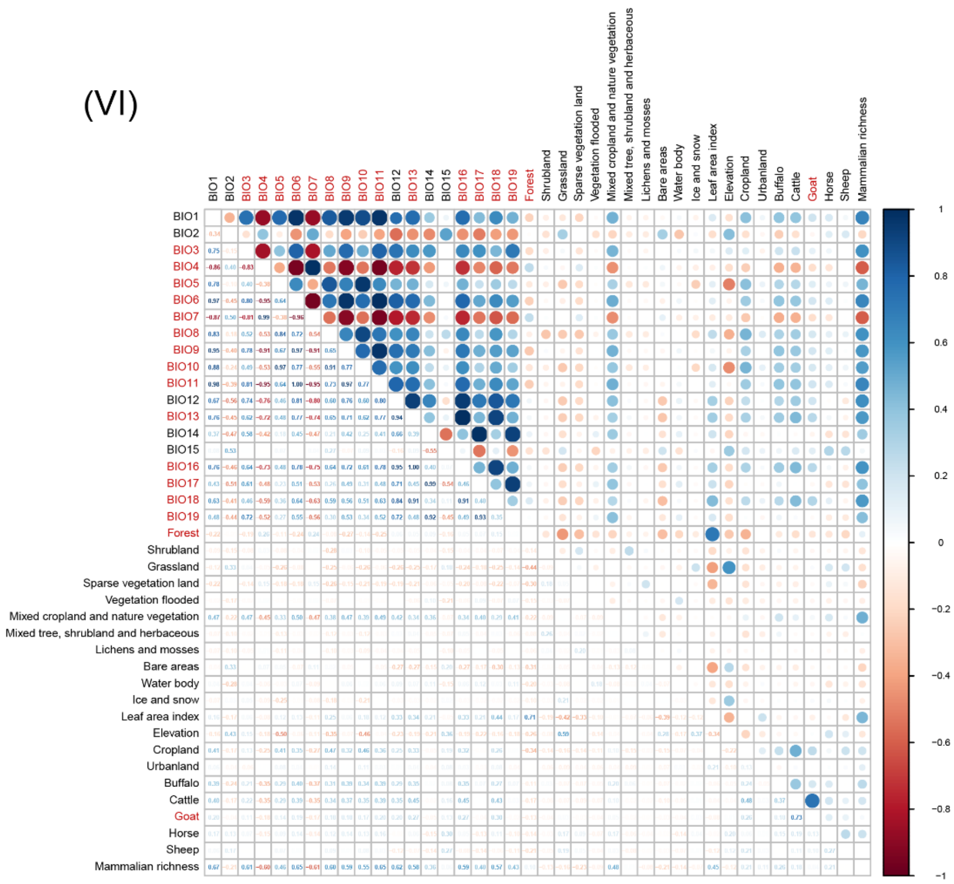
BIO1
BIO2
BIO3
BIO4
BIO5
BIO6
BIO7
BIO8
BIO9
BIO10
BIO11
BIO12
BIO13
BIO14
BIO15
BIO16
BIO17
BIO18
BIO19
Forest
Shrubland
Grassland
Sparse vegetation land
Vegetation flooded
Mixed cropland and nature vegetation
Mixed tree, shrubland and herbaceous
Lichens and mosses
Bare areas
Water body
Ice and snow
Leaf area index
Elevation
Cropland
Urbanland
Buffalo
Cattle
Goat
Horse
Sheep
Mammalian richness
1
0.8
0.6
0.4
0.2
0
−0.2
−0.4
−0.6
−0.8
−1

(VII)

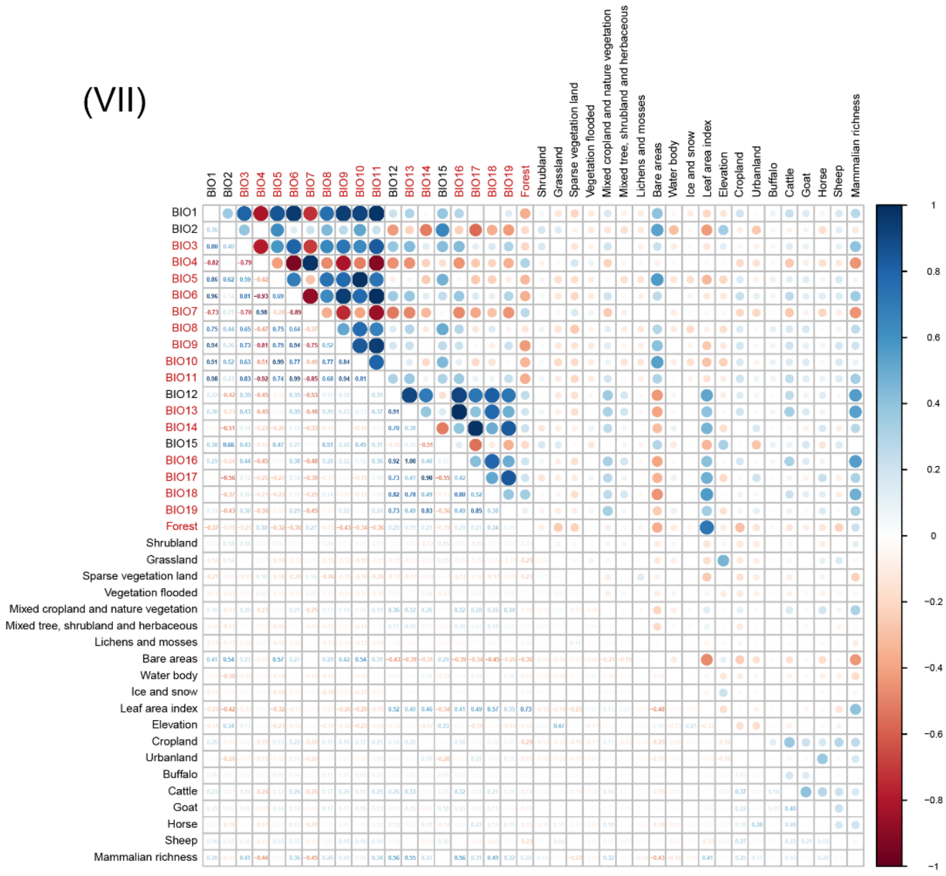
BIO1
BIO2
BIO3
BIO4
BIO5
BIO6
BIO7
BIO8
BIO9
BIO10
BIO11
BIO12
BIO13
BIO14
BIO15
BIO16
BIO17
BIO18
BIO19
Forest
Shrubland
Grassland
Sparse vegetation land
Vegetation flooded
Mixed cropland and nature vegetation
Mixed tree, shrubland and herbaceous
Lichens and mosses
Bare areas
Water body
Ice and snow
Leaf area index
Elevation
Cropland
Urbanland
Buffalo
Cattle
Goat
Horse
Sheep
Mammalian richness
1
0.8
0.6
0.4
0.2
0
−0.2
−0.4
−0.6
−0.8
−1

(VIII)

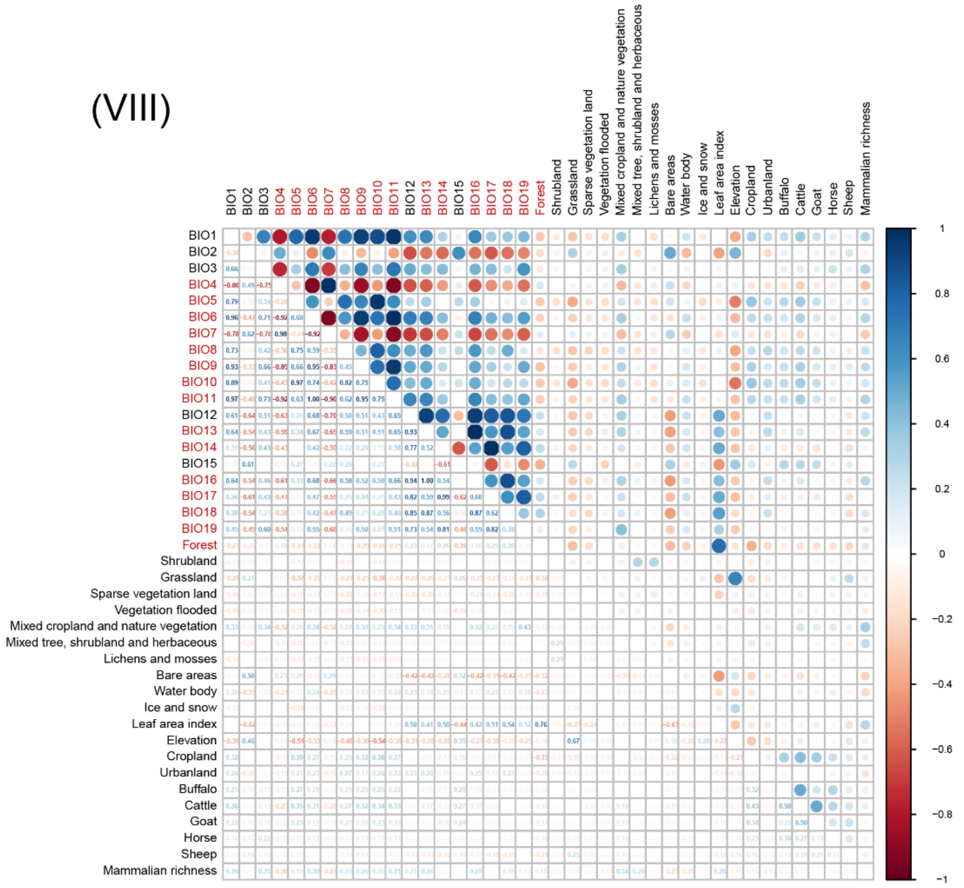

BIO1
BIO2
BIO3
BIO4
BIO5
BIO6
BIO7
BIO8
BIO9
BIO10
BIO11
BIO12
BIO13
BIO14
BIO15
BIO16
BIO17
BIO18
BIO19
Forest
Shrubland
Grassland
Sparse vegetation land
Vegetation flooded
Mixed cropland and nature vegetation
Mixed tree, shrubland and herbaceous
Lichens and mosses
Bare areas
Water body
Ice and snow
Leaf area index
Elevation
Cropland
Urbanland
Buffalo
Cattle
Goat
Horse
Sheep
Mammalian richness
1
0.8
0.6
0.4
0.2
0
−0.2
−0.4
−0.6
−0.8
−1

(IX)

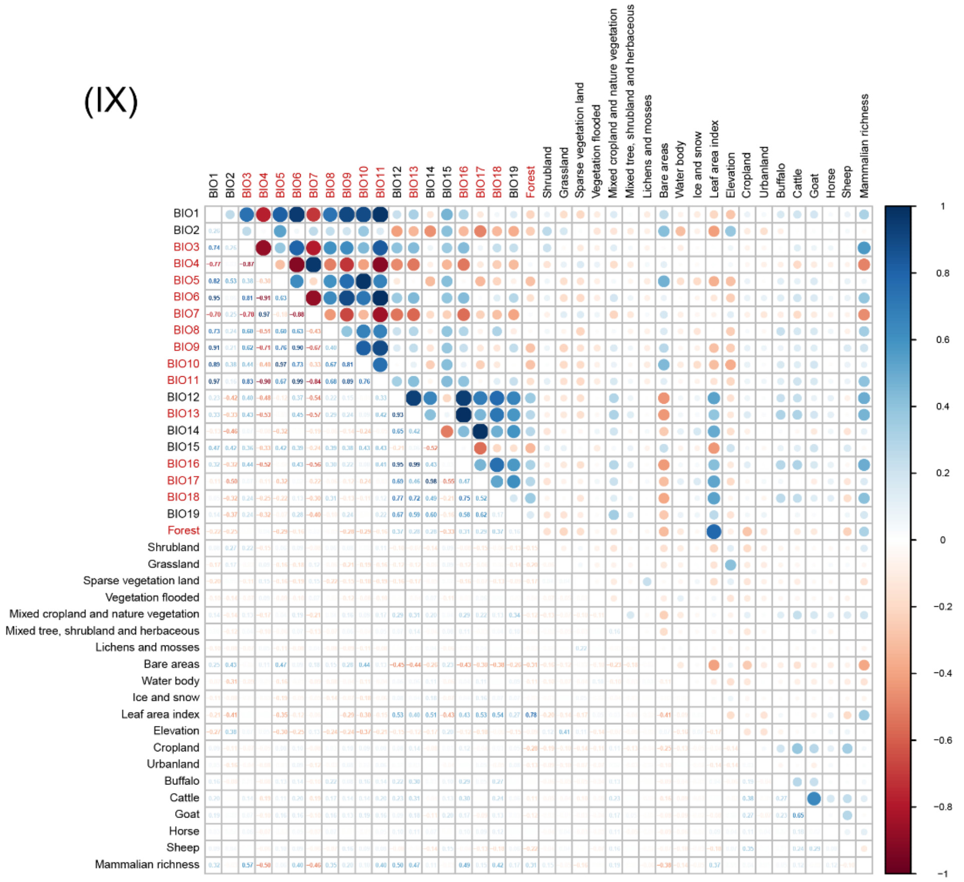

BIO1
BIO2
BIO3
BIO4
BIO5
BIO6
BIO7
BIO8
BIO9
BIO10
BIO11
BIO12
BIO13
BIO14
BIO15
BIO16
BIO17
BIO18
BIO19
Forest
Shrubland
Grassland
Sparse vegetation land
Vegetation flooded
Mixed cropland and nature vegetation
Mixed tree, shrubland and herbaceous
Lichens and mosses
Bare areas
Water body
Ice and snow
Leaf area index
Elevation
Cropland
Urbanland
Buffalo
Cattle
Goat
Horse
Sheep
Mammalian richness
1
0.8
0.6
0.4
0.2
0
−0.2
−0.4
−0.6
−0.8
−1

(X)

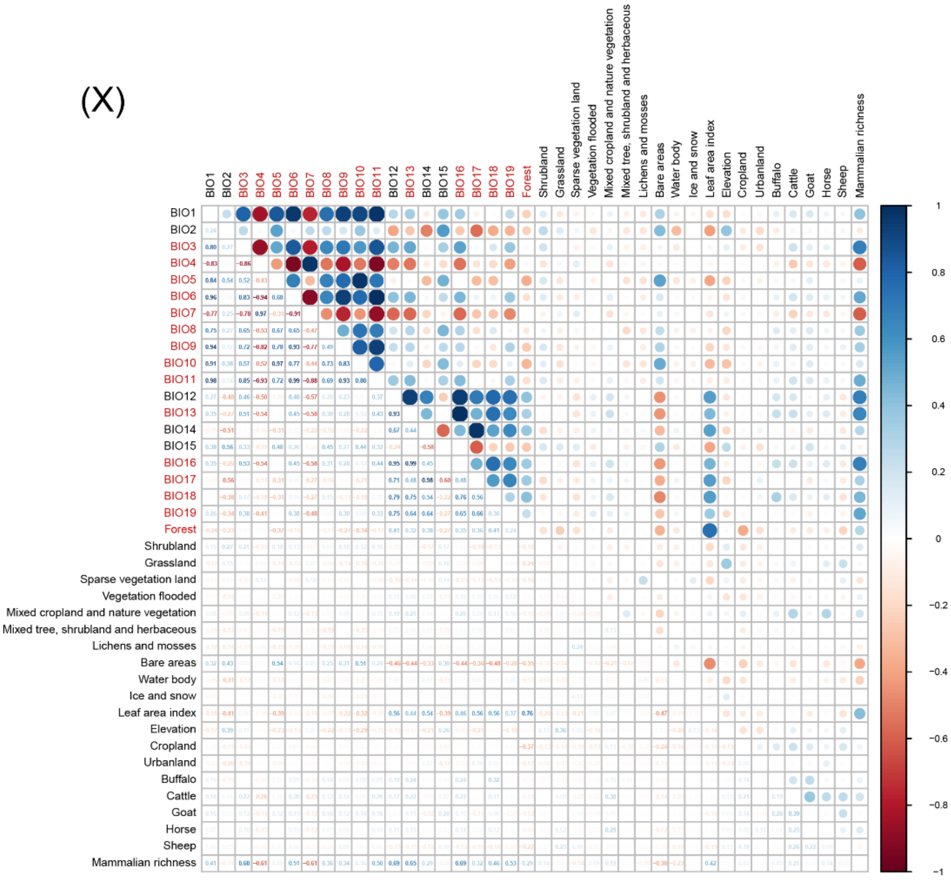
BIO1
BIO2
BIO3
BIO4
BIO5
BIO6
BIO7
BIO8
BIO9
BIO10
BIO11
BIO12
BIO13
BIO14
BIO15
BIO16
BIO17
BIO18
BIO19
Forest
Shrubland
Grassland
Sparse vegetation land
Vegetation flooded
Mixed cropland and nature vegetation
Mixed tree, shrubland and herbaceous
Lichens and mosses
Bare areas
Water body
Ice and snow
Leaf area index
Elevation
Cropland
Urbanland
Buffalo
Cattle
Goat
Horse
Sheep
Mammalian richness
1
0.8
0.6
0.4
0.2
0
−0.2
−0.4
−0.6
−0.8
−1

(XI)

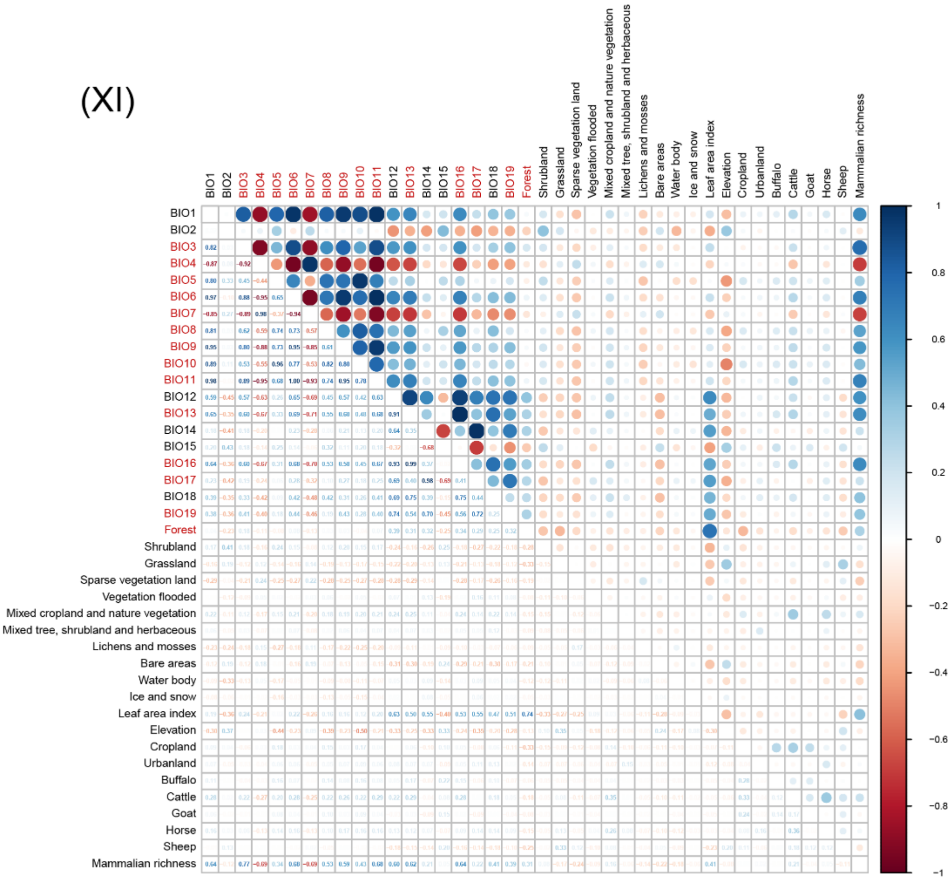
BIO1
BIO2
BIO3
BIO4
BIO5
BIO6
BIO7
BIO8
BIO9
BIO10
BIO11
BIO12
BIO13
BIO14
BIO15
BIO16
BIO17
BIO18
BIO19
Forest
Shrubland
Grassland
Sparse vegetation land
Vegetation flooded
Mixed cropland and nature vegetation
Mixed tree, shrubland and herbaceous
Lichens and mosses
Bare areas
Water body
Ice and snow
Leaf area index
Elevation
Cropland
Urbanland
Buffalo
Cattle
Goat
Horse
Sheep
Mammalian richness
1
0.8
0.6
0.4
0.2
0
−0.2
−0.4
−0.6
−0.8
−1

(XII)

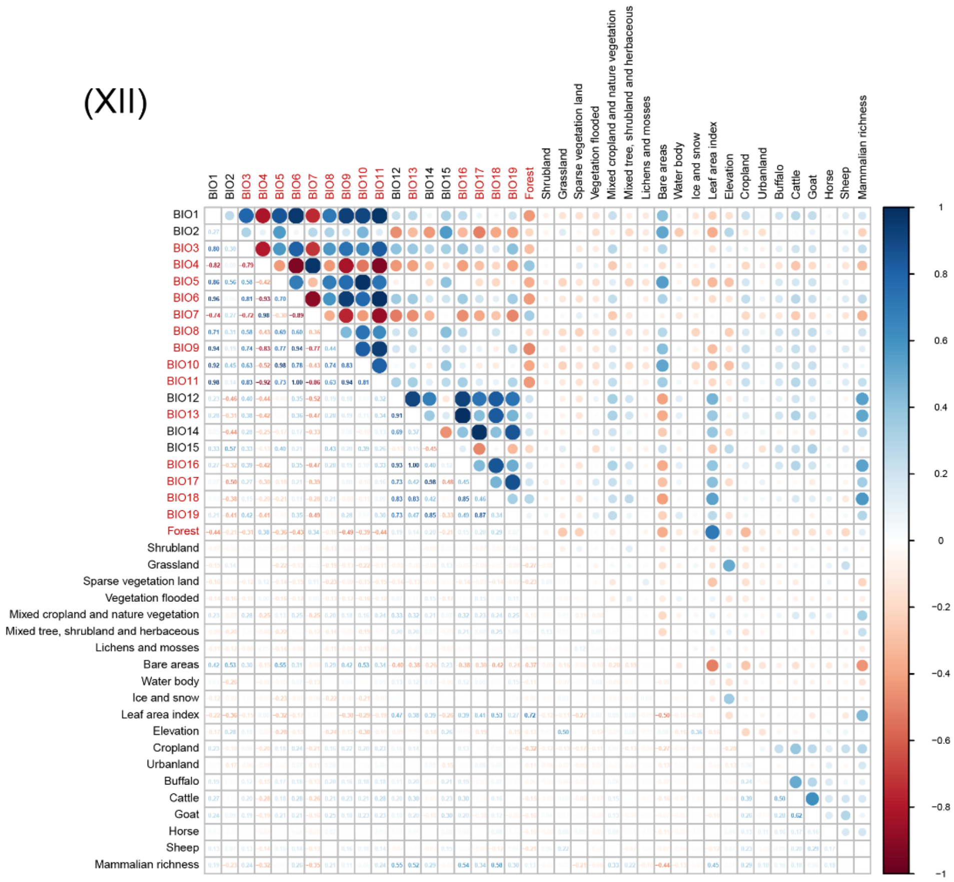

BIO1
BIO2
BIO3
BIO4
BIO5
BIO6
BIO7
BIO8
BIO9
BIO10
BIO11
BIO12
BIO13
BIO14
BIO15
BIO16
BIO17
BIO18
BIO19
Forest
Shrubland
Grassland
Sparse vegetation land
Vegetation flooded
Mixed cropland and nature vegetation
Mixed tree, shrubland and herbaceous
Lichens and mosses
Bare areas
Water body
Ice and snow
Leaf area index
Elevation
Cropland
Urbanland
Buffalo
Cattle
Goat
Horse
Sheep
Mammalian richness
1
0.8
0.6
0.4
0.2
0
−0.2
−0.4
−0.6
−0.8
−1

(XIII)

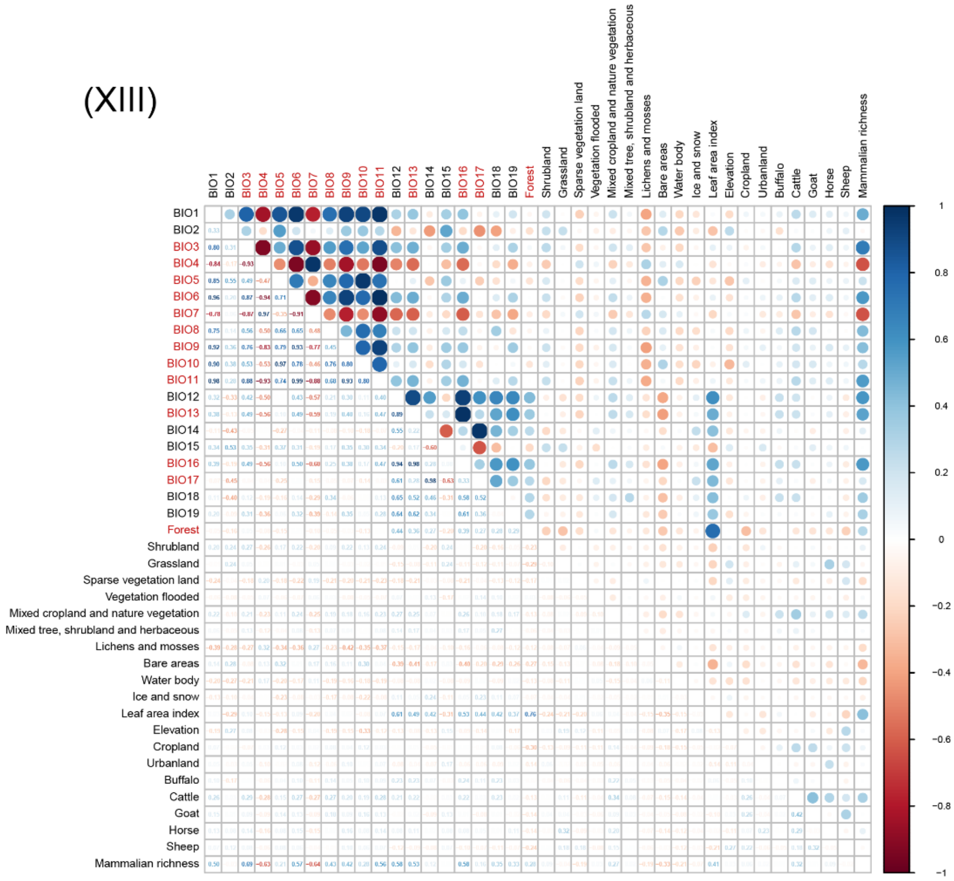

BIO1
BIO2
BIO3
BIO4
BIO5
BIO6
BIO7
BIO8
BIO9
BIO10
BIO11
BIO12
BIO13
BIO14
BIO15
BIO16
BIO17
BIO18
BIO19
Forest
Shrubland
Grassland
Sparse vegetation land
Vegetation flooded
Mixed cropland and nature vegetation
Mixed tree, shrubland and herbaceous
Lichens and mosses
Bare areas
Water body
Ice and snow
Leaf area index
Elevation
Cropland
Urbanland
Buffalo
Cattle
Goat
Horse
Sheep
Mammalian richness
1
0.8
0.6
0.4
0.2
0
−0.2
−0.4
−0.6
−0.8
−1

(XIV)

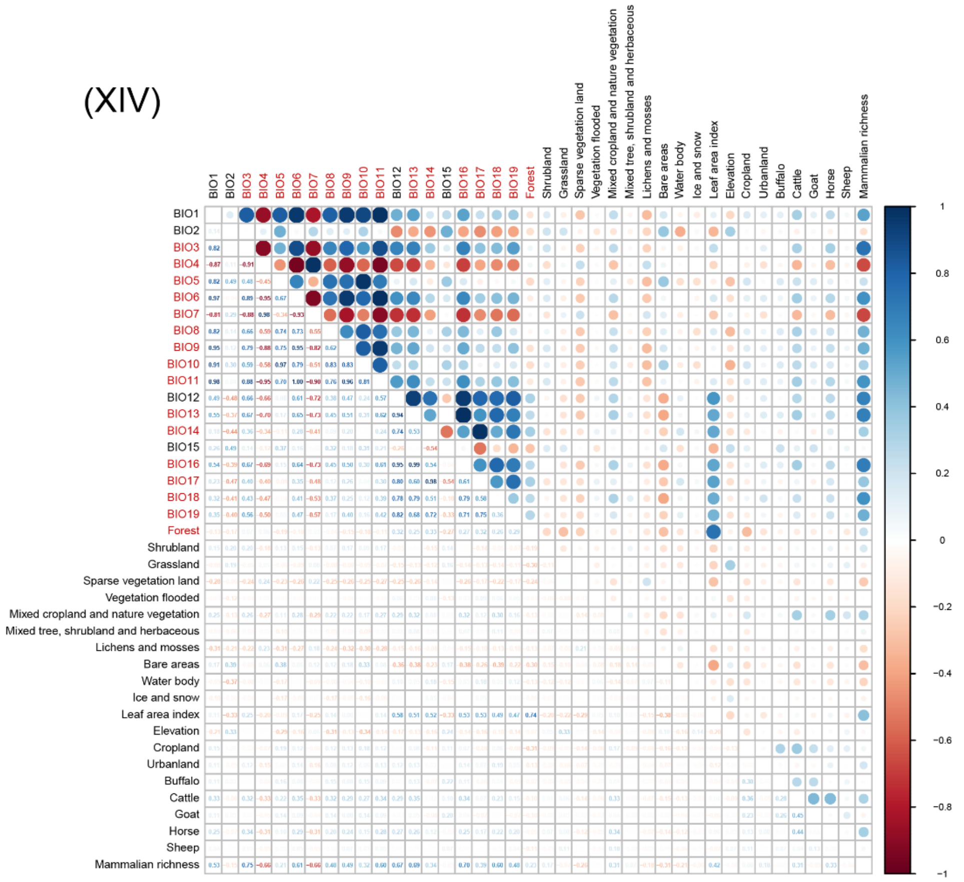
BIO1
BIO2
BIO3
BIO4
BIO5
BIO6
BIO7
BIO8
BIO9
BIO10
BIO11
BIO12
BIO13
BIO14
BIO15
BIO16
BIO17
BIO18
BIO19
Forest
Shrubland
Grassland
Sparse vegetation land
Vegetation flooded
Mixed cropland and nature vegetation
Mixed tree, shrubland and herbaceous
Lichens and mosses
Bare areas
Water body
Ice and snow
Leaf area index
Elevation
Cropland
Urbanland
Buffalo
Cattle
Goat
Horse
Sheep
Mammalian richness
1
0.8
0.6
0.4
0.2
0
−0.2
−0.4
−0.6
−0.8
−1

(XV)

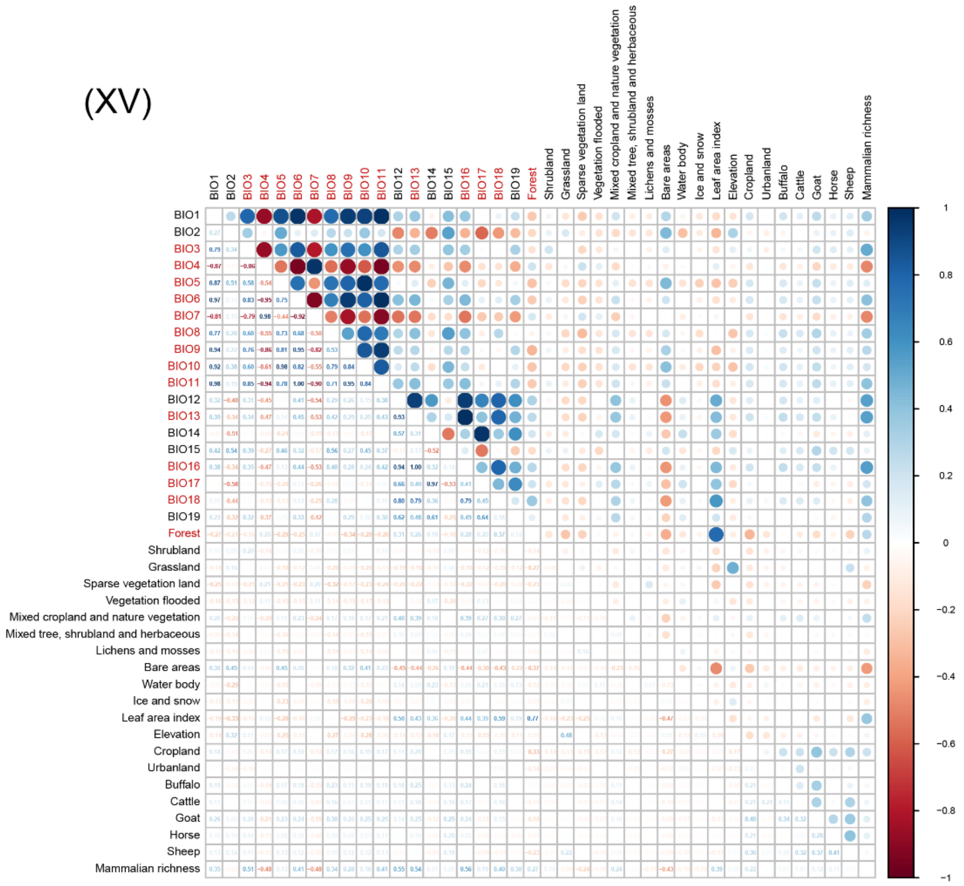
BIO1
BIO2
BIO3
BIO4
BIO5
BIO6
BIO7
BIO8
BIO9
BIO10
BIO11
BIO12
BIO13
BIO14
BIO15
BIO16
BIO17
BIO18
BIO19
Forest
Shrubland
Grassland
Sparse vegetation land
Vegetation flooded
Mixed cropland and nature vegetation
Mixed tree, shrubland and herbaceous
Lichens and mosses
Bare areas
Water body
Ice and snow
Leaf area index
Elevation
Cropland
Urbanland
Buffalo
Cattle
Goat
Horse
Sheep
Mammalian richness
1
0.8
0.6
0.4
0.2
0
−0.2
−0.4
−0.6
−0.8
−1

(XVI)

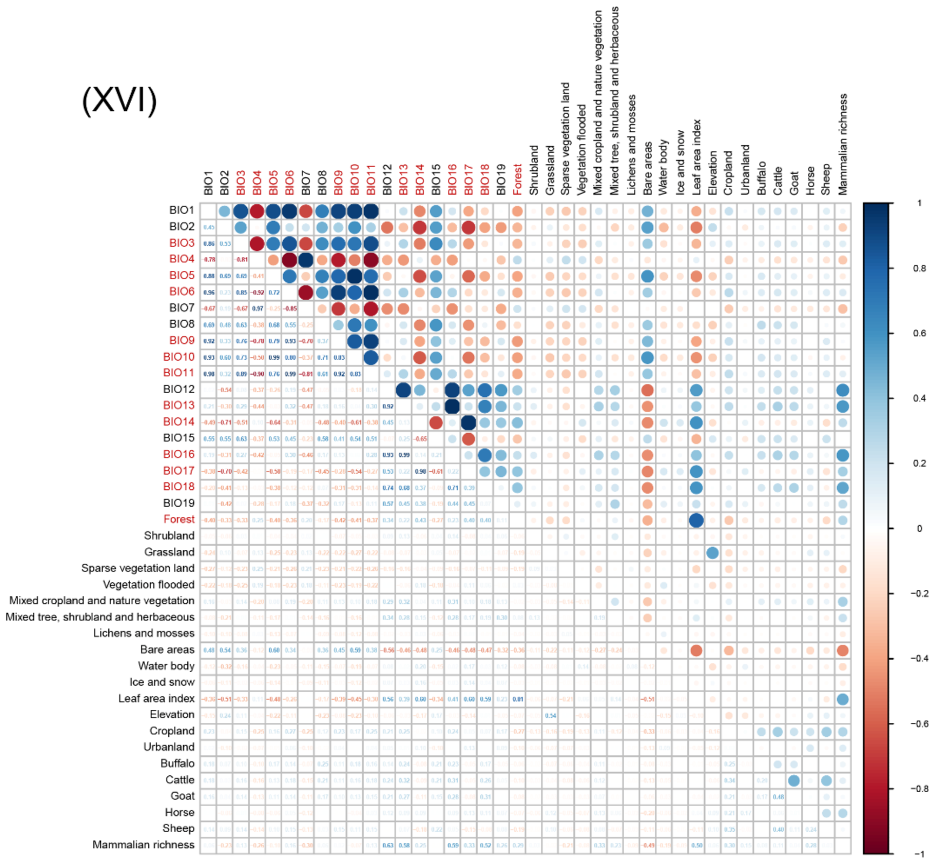

BIO1
BIO2
BIO3
BIO4
BIO5
BIO6
BIO7
BIO8
BIO9
BIO10
BIO11
BIO12
BIO13
BIO14
BIO15
BIO16
BIO17
BIO18
BIO19
Forest
Shrubland
Grassland
Sparse vegetation land
Vegetation flooded
Mixed cropland and nature vegetation
Mixed tree, shrubland and herbaceous
Lichens and mosses
Bare areas
Water body
Ice and snow
Leaf area index
Elevation
Cropland
Urbanland
Buffalo
Cattle
Goat
Horse
Sheep
Mammalian richness
1
0.8
0.6
0.4
0.2
0
−0.2
−0.4
−0.6
−0.8
−1

(XVII)

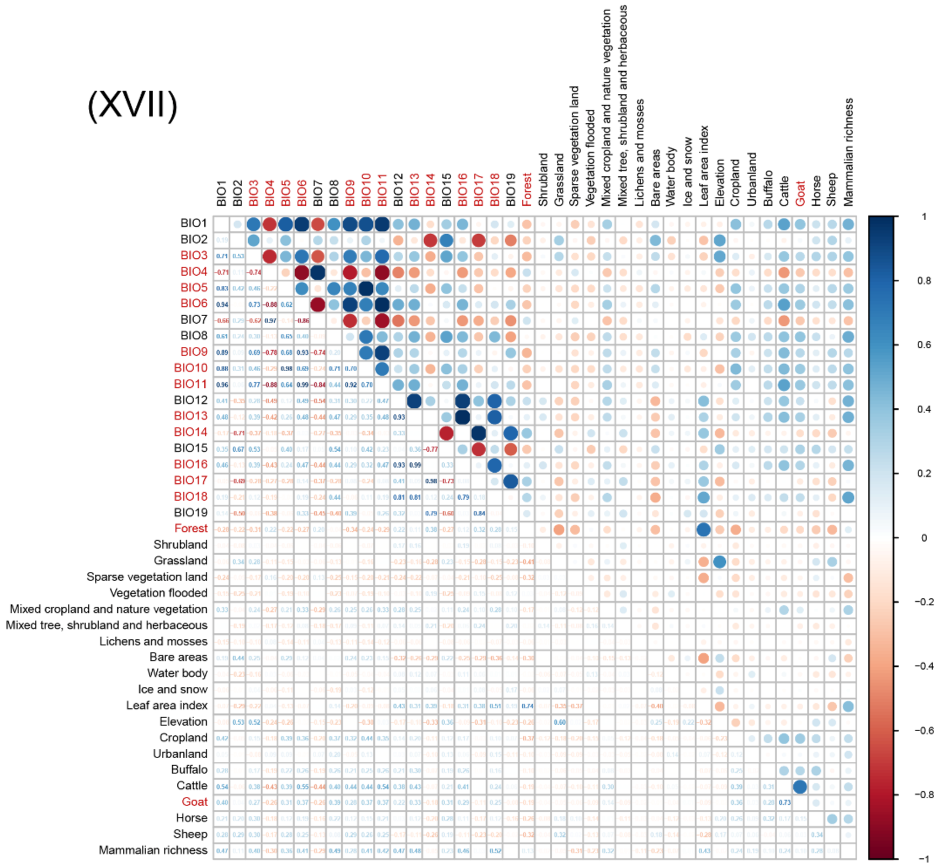

BIO1
BIO2
BIO3
BIO4
BIO5
BIO6
BIO7
BIO8
BIO9
BIO10
BIO11
BIO12
BIO13
BIO14
BIO15
BIO16
BIO17
BIO18
BIO19
Forest
Shrubland
Grassland
Sparse vegetation land
Vegetation flooded
Mixed cropland and nature vegetation
Mixed tree, shrubland and herbaceous
Lichens and mosses
Bare areas
Water body
Ice and snow
Leaf area index
Elevation
Cropland
Urbanland
Buffalo
Cattle
Goat
Horse
Sheep
Mammalian richness
1
0.8
0.6
0.4
0.2
0
−0.2
−0.4
−0.6
−0.8
−1

Supplementary figure 2: The relationship matrix of SFGR species and involved ticks. The SFGR species determined to infect humans are marked by red fonts. Names of ticks are marked in blue if they bite humans. The purple square indicates pathogenic SFGR to humans carried by human-biting vectors, and it turns into green if the vector was not found to bite humans. Orange squares indicate rickettsiae were not pathogenic to humans carried by human-biting vectors, and it turns into grey if carried by vectors of non-biting human.

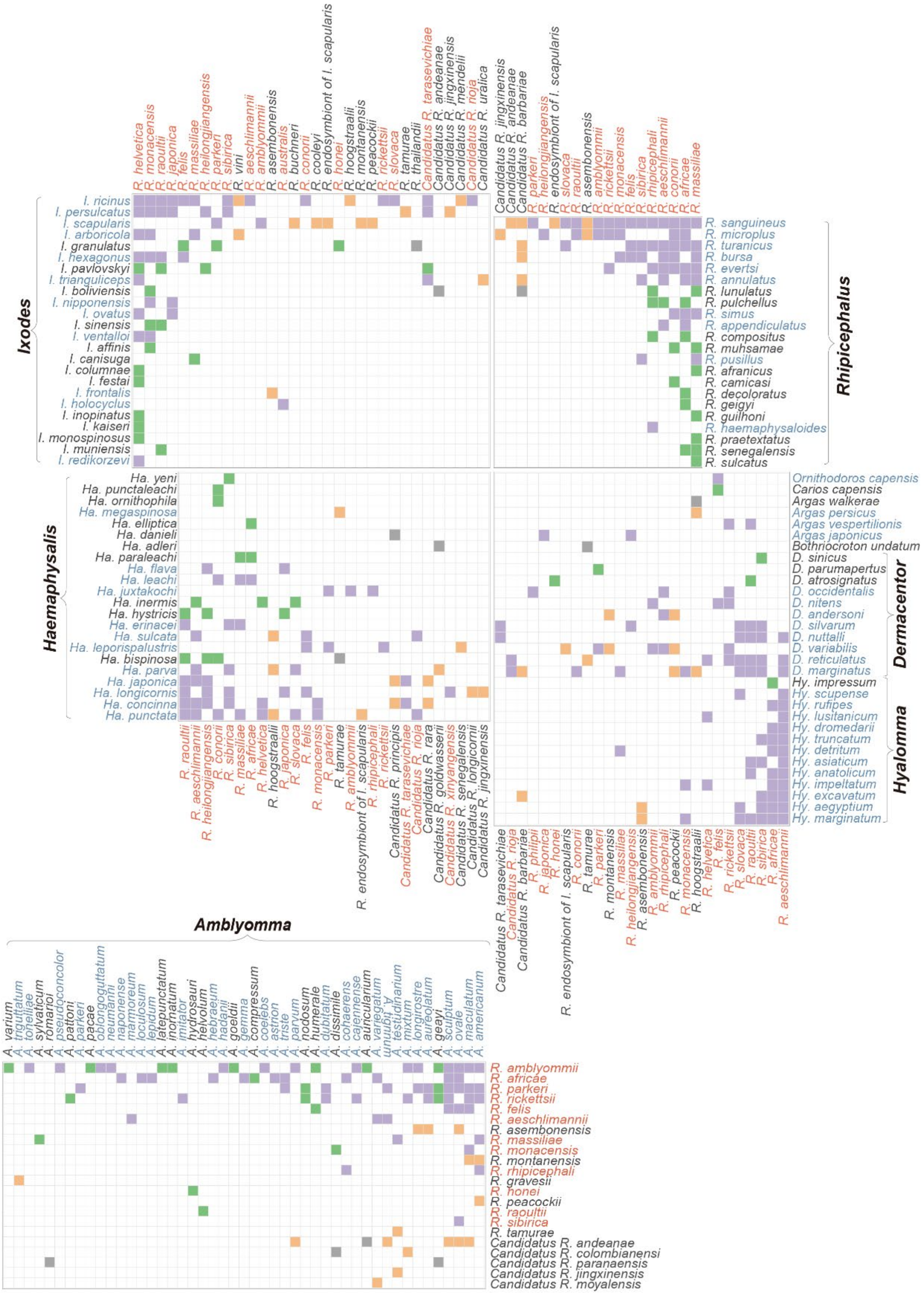

Supplementary figure 3: The relationship matrix of SFGR species and other vectors. The SFGR species determined to infect humans are marked by red fonts. Names of vectors are marked in blue if they bite humans. The purple square indicates pathogenic SFGR to humans carried by human-biting vectors, and it turns into green if the vector was not found to bite humans. Orange squares indicate rickettsiae were not pathogenic to humans carried by human-biting vectors, and it turns into grey if carried by vectors of non-biting human.

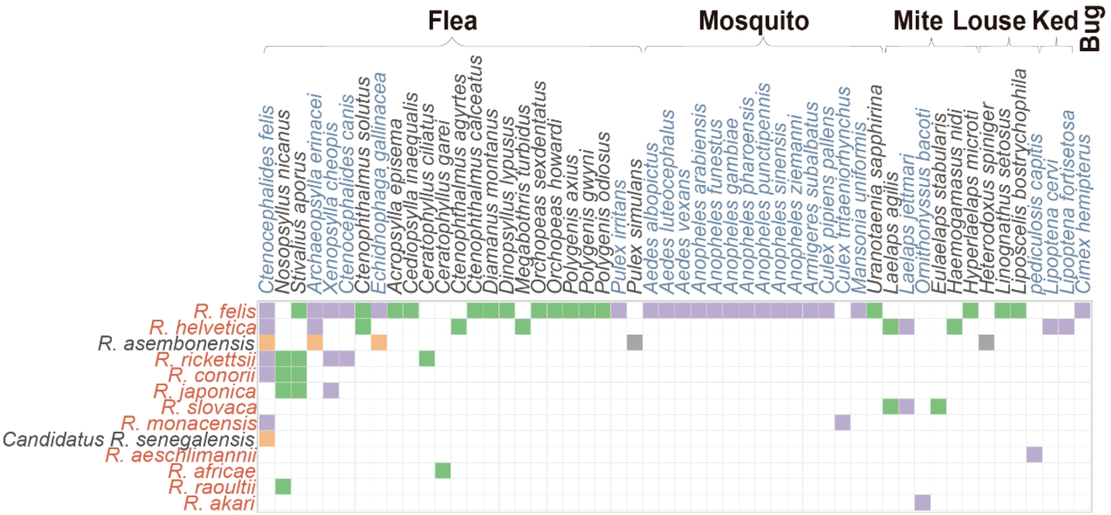

Supplementary figure 4: The relationship matrix of SFGR species and animals.

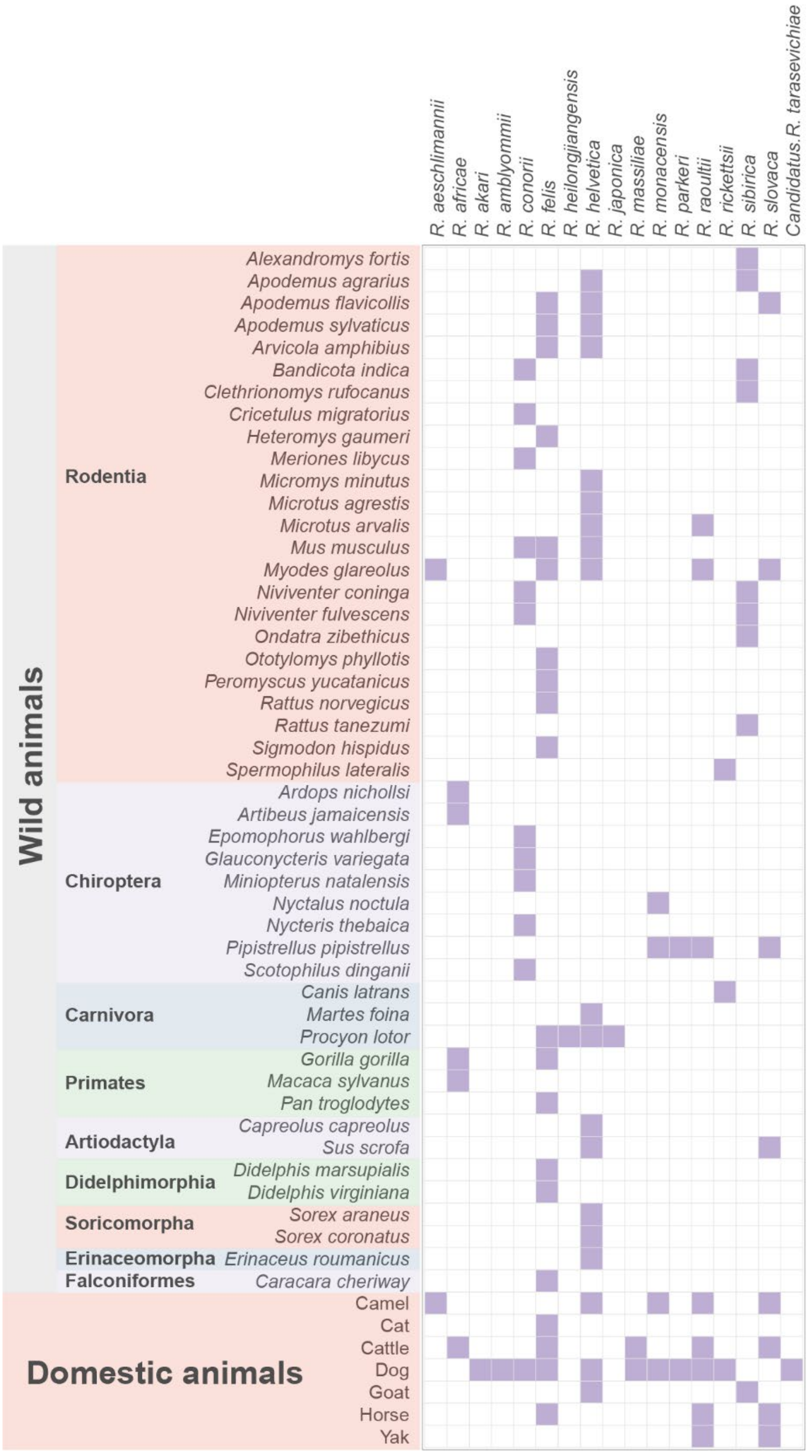

Supplementary figure 5: SFGR species richness (red circles) at the country level in four continents. The geographical regions (country or area) are divided based on the United Nations "Standard Country or Area Codes for Statistical Use" (https://unstats.un.org/unsd/methodology/m49/).

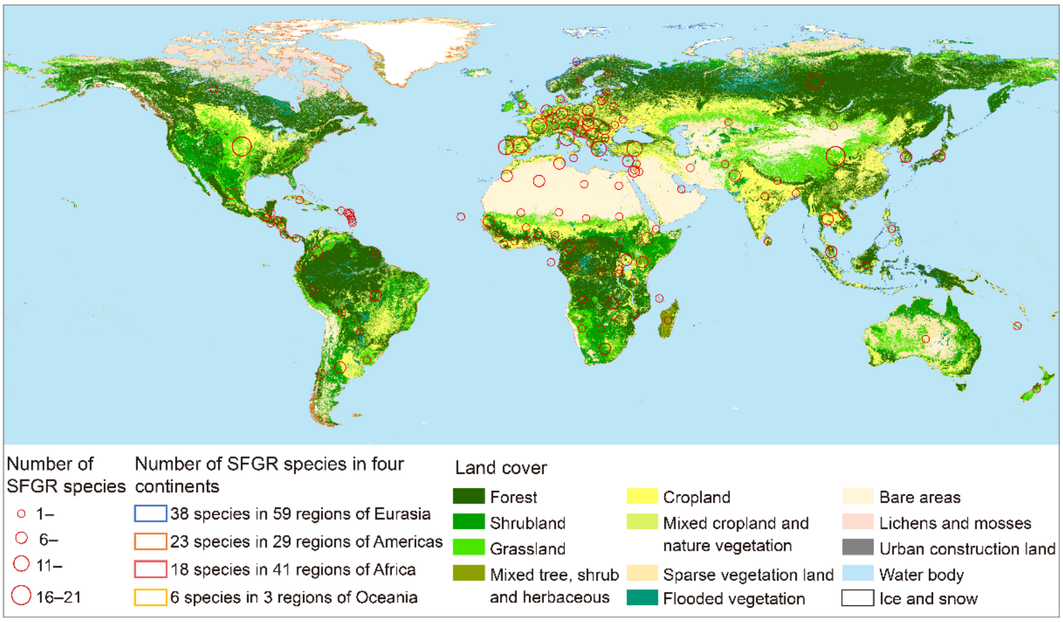

Supplementary figure 6: The globally geographical distributions of the non-predominant 31 SFGR species.

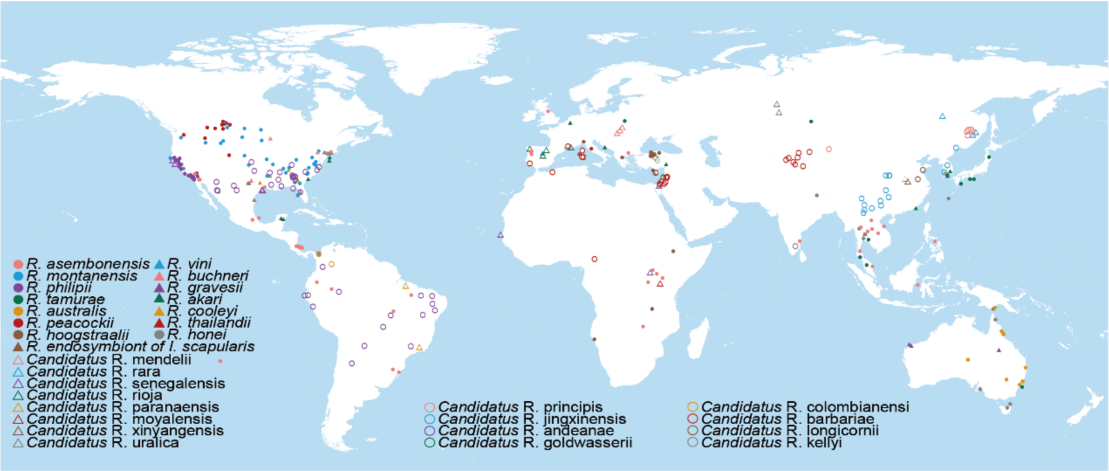

Supplementary Figure 7: Comparing predictive performance of the three machine-learning algorithms. ROC curves and AUC values of the BRT (left, red), RF (middle, blue) and LASSO regression (right, orange) over 100 models are shown. Roman numerals 1-17 correspond to the 17 predominant SFGR species: (I) *R. aeschlimannii*; (II) *R. africae*; (III) *R. amblyommii*; (IV) *R. conorii*; (V) *R. felis*; (VI) *R. heilongjiangensis*; (VII) *R. helvetica*; (VIII) *R. japonica*; (IX) *R. massiliae*; (X) *R. monacensis*; (XI) *R. parkeri*; (XII) *R. raoultii*; (XIII) *R. rhipicephali*; (XIV) *R. rickettsii*; (XV) *R. sibirica*; (XVI) *R. slovaca*; (XVII) *Candidatus* R. tarasevichiae.

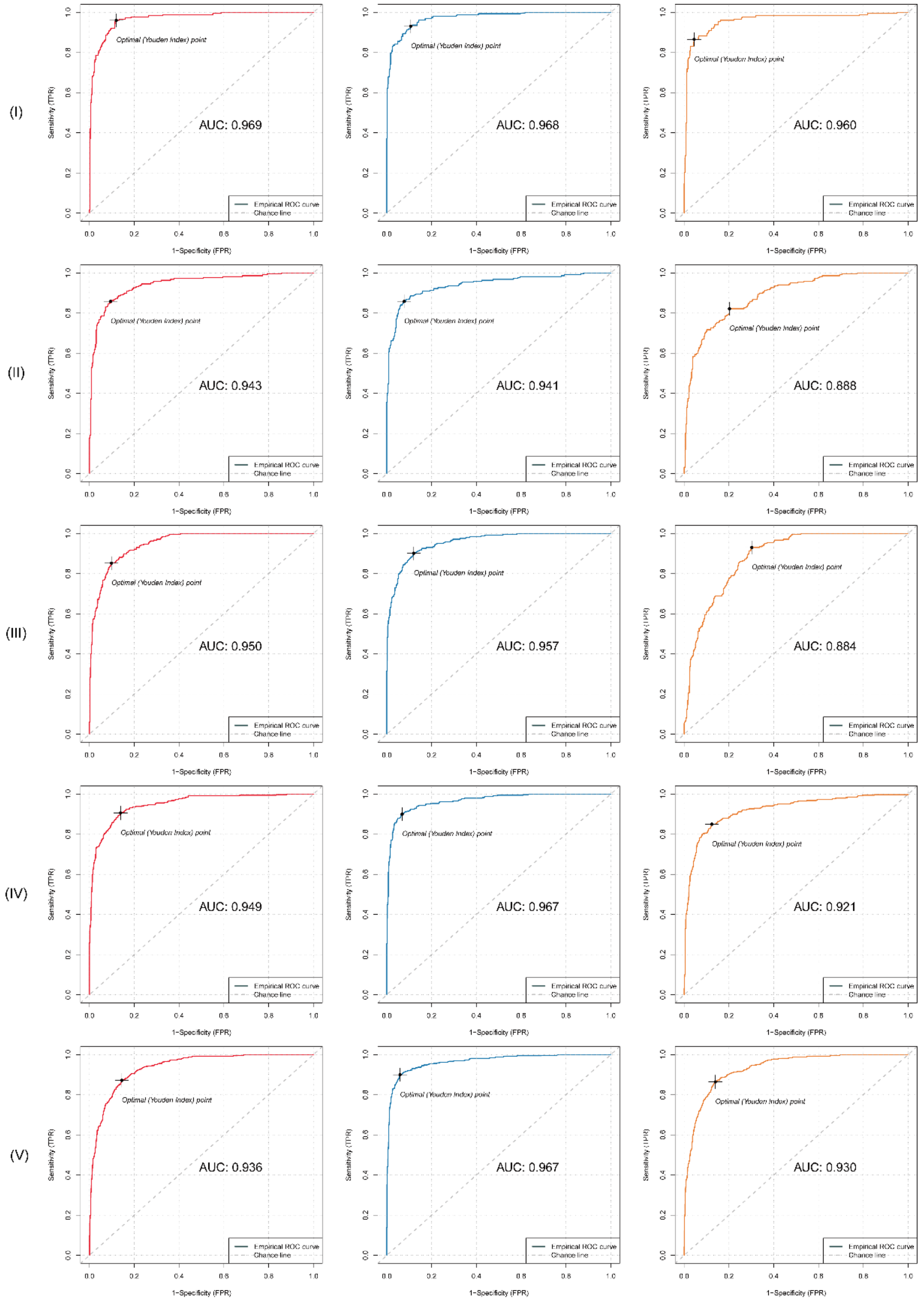

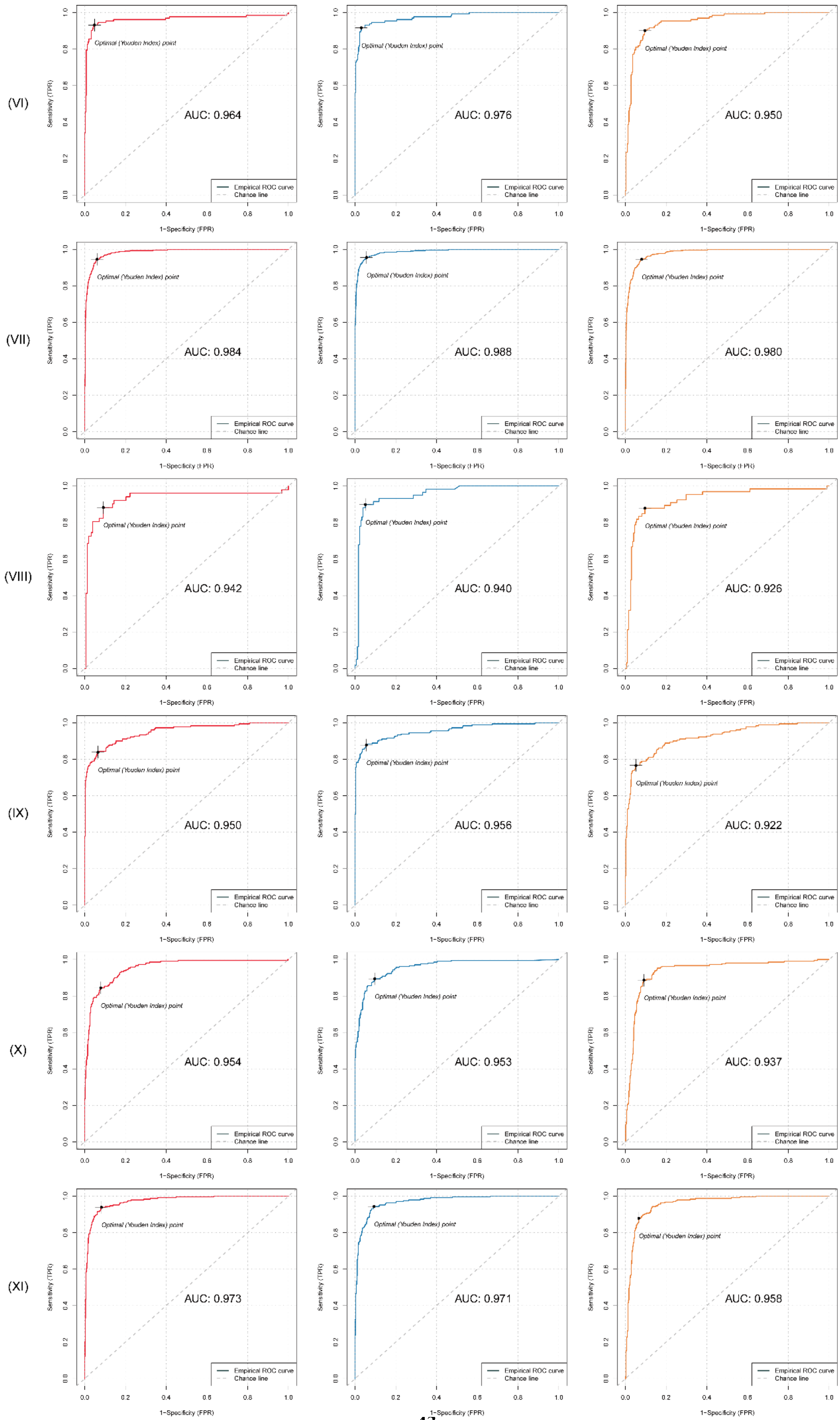
(VI)
AUC: 0.964
AUC: 0.976
AUC: 0.950
(VII)
AUC: 0.984
AUC: 0.988
AUC: 0.980
(VIII)
AUC: 0.942
AUC: 0.940
AUC: 0.926
(IX)
AUC: 0.950
AUC: 0.956
AUC: 0.922
(X)
AUC: 0.954
AUC: 0.953
AUC: 0.937
(XI)
AUC: 0.973
AUC: 0.971
AUC: 0.958
Optimal (Youden Index) point
Empirical ROC curve
Chance line
Sensitivity (TPR)
1-Specificity (FPR)

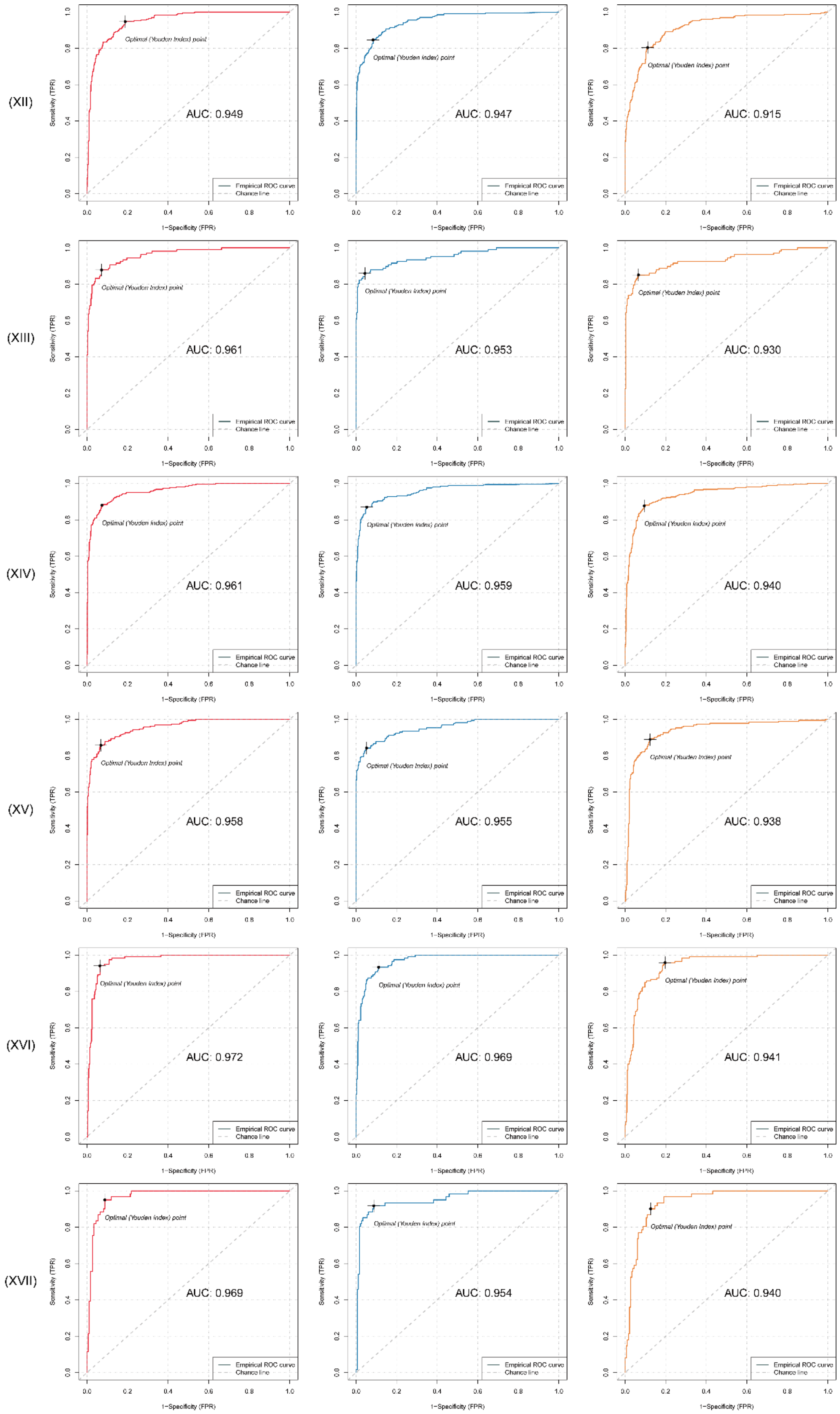
(XII)
AUC: 0.949
AUC: 0.947
AUC: 0.915
(XIII)
AUC: 0.961
AUC: 0.953
AUC: 0.930
(XIV)
AUC: 0.961
AUC: 0.959
AUC: 0.940
(XV)
AUC: 0.958
AUC: 0.955
AUC: 0.938
(XVI)
AUC: 0.972
AUC: 0.969
AUC: 0.941
(XVII)
AUC: 0.969
AUC: 0.954
AUC: 0.940
Optimal (Youden index) point
Empirical ROC curve
Chance line
Sensitivity (TPR)
1−Specificity (FPR)

Supplementary Figure 8: Effects of major predictors (relative contributions >3%) for presence of *R. aeschlimannii* based on BRT models. The mean curves (red) and 95% percentiles (gray) show the predicted probability of occurrence at the logit scale. The histograms (blue) show the frequency distributions of the predictors.

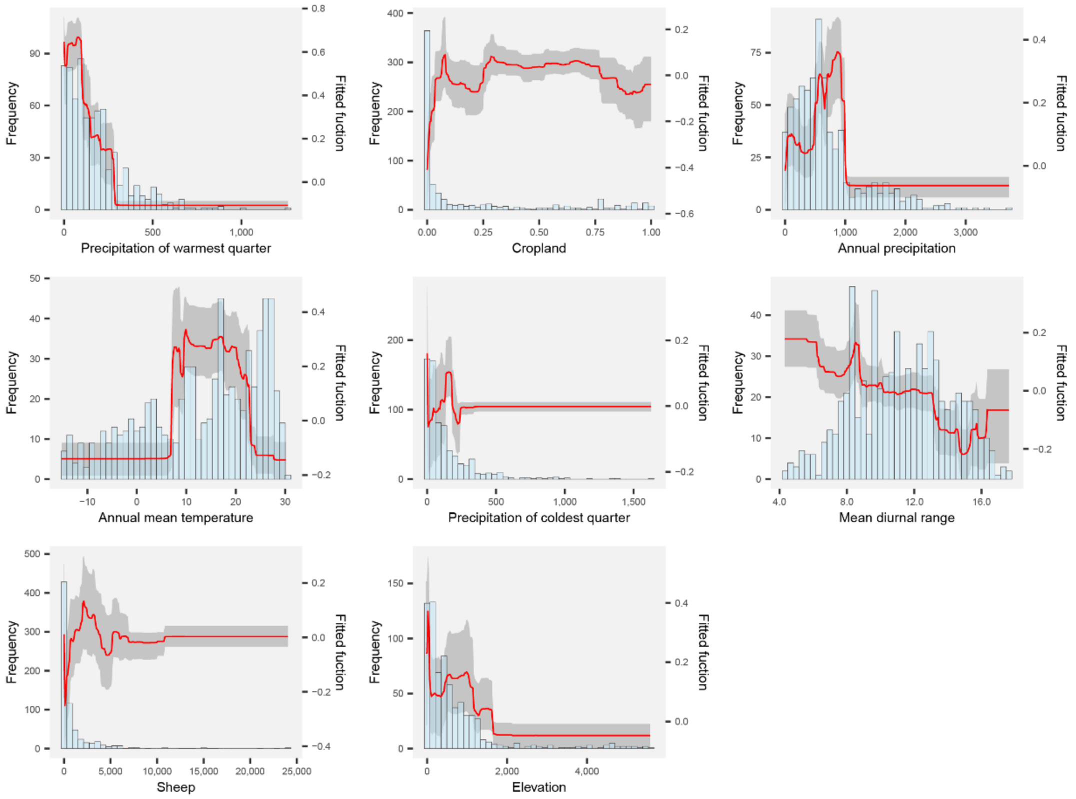

Supplementary Figure 9: Effects of major predictors (relative contributions >3%) for presence of *R. africae* based on BRT models. The mean curves (red) and 95% percentiles (gray) show the predicted probability of occurrence at the logit scale. The histograms (blue) show the frequency distributions of the predictors.

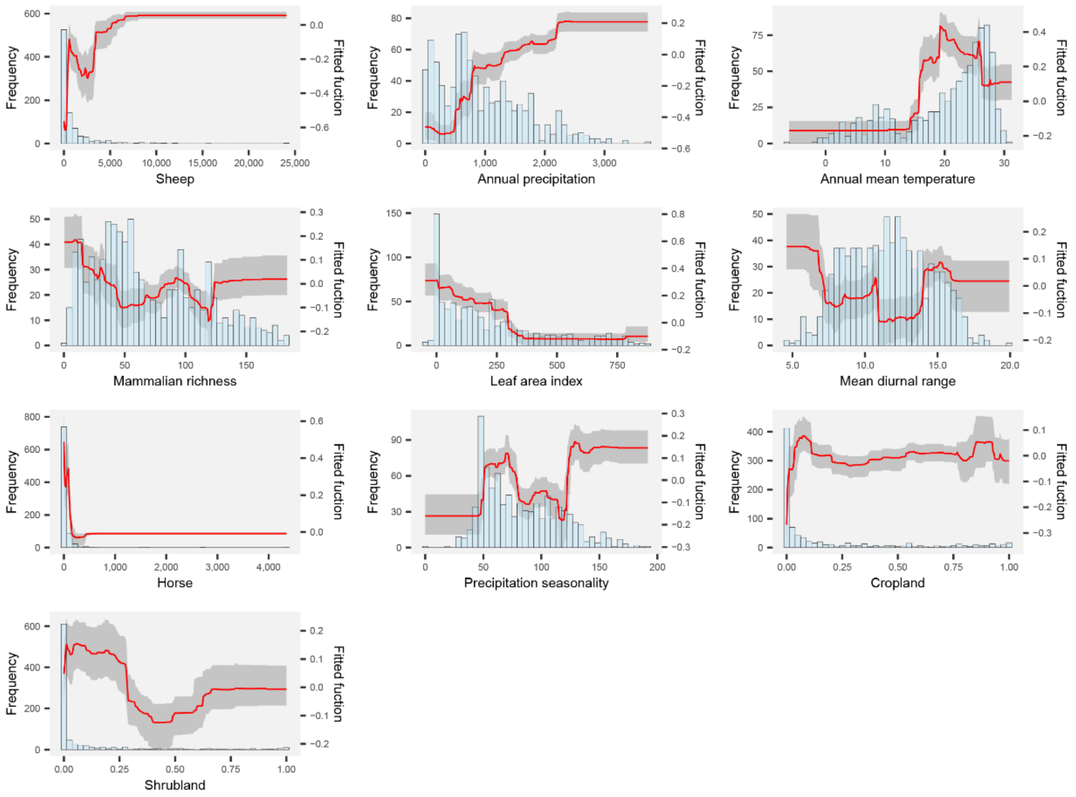

Supplementary Figure 10: Effects of major predictors (relative contributions >3%) for presence of *R. amblyommii* based on BRT models. The mean curves (red) and 95% percentiles (gray) show the predicted probability of occurrence at the logit scale. The histograms (blue) show the frequency distributions of the predictors.

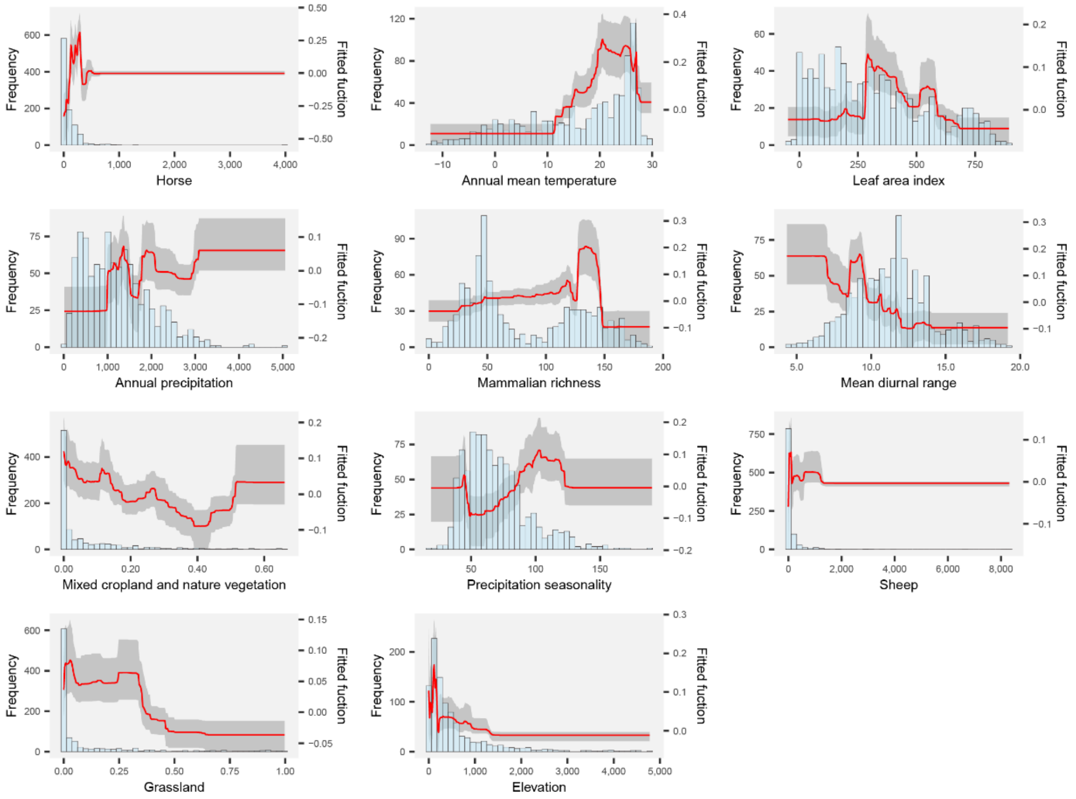

Supplementary Figure 11: Effects of major predictors (relative contributions >3%) for presence of *R. conorii* based on BRT models. The mean curves (red) and 95% percentiles (gray) show the predicted probability of occurrence at the logit scale. The histograms (blue) show the frequency distributions of the predictors.

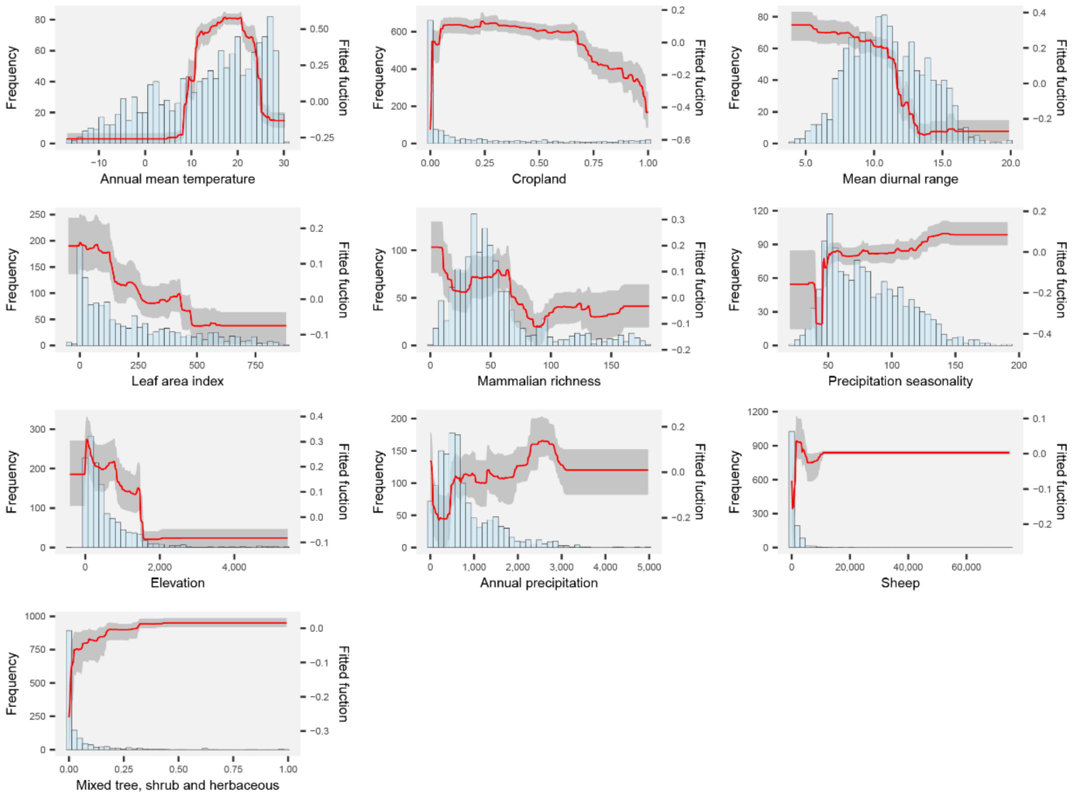

Supplementary Figure 12: Effects of major predictors (relative contributions >3%) for presence of *R. felis* based on BRT models. The mean curves (red) and 95% percentiles (gray) show the predicted probability of occurrence at the logit scale. The histograms (blue) show the frequency distributions of the predictors.

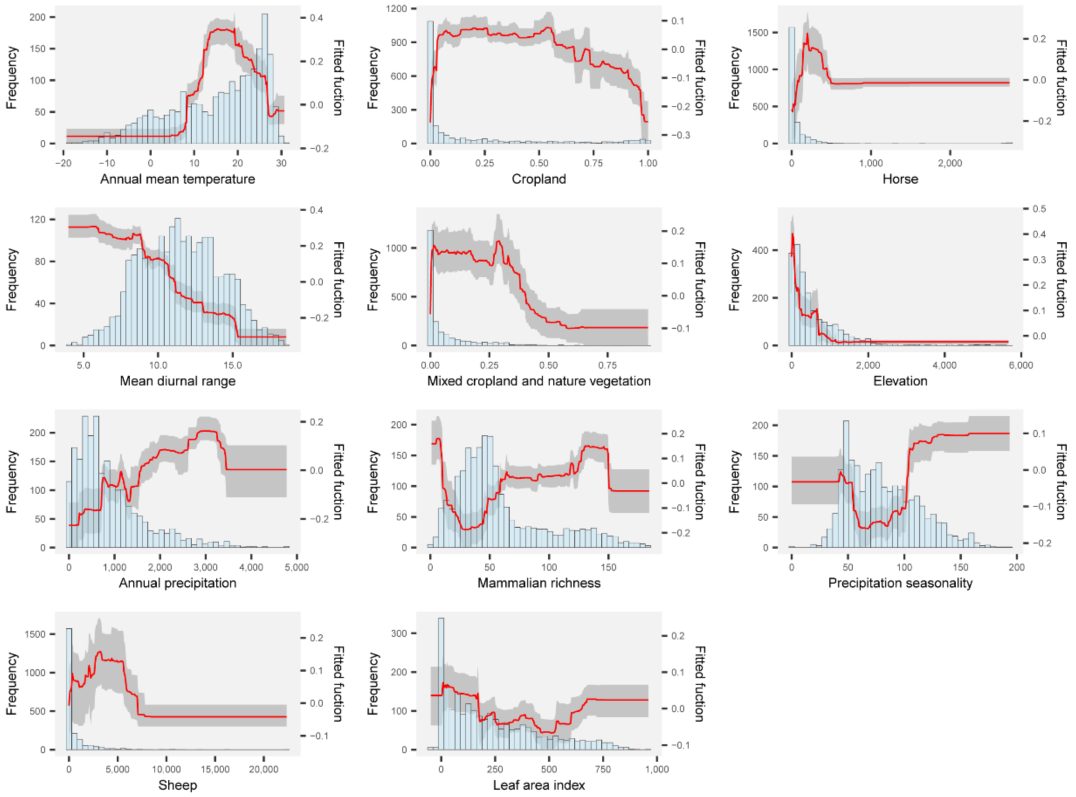

Supplementary Figure 13: Effects of major predictors (relative contributions >3%) for presence of *R. heilongjiangensis* based on BRT models. The mean curves (red) and 95% percentiles (gray) show the predicted probability of occurrence at the logit scale. The histograms (blue) show the frequency distributions of the predictors.

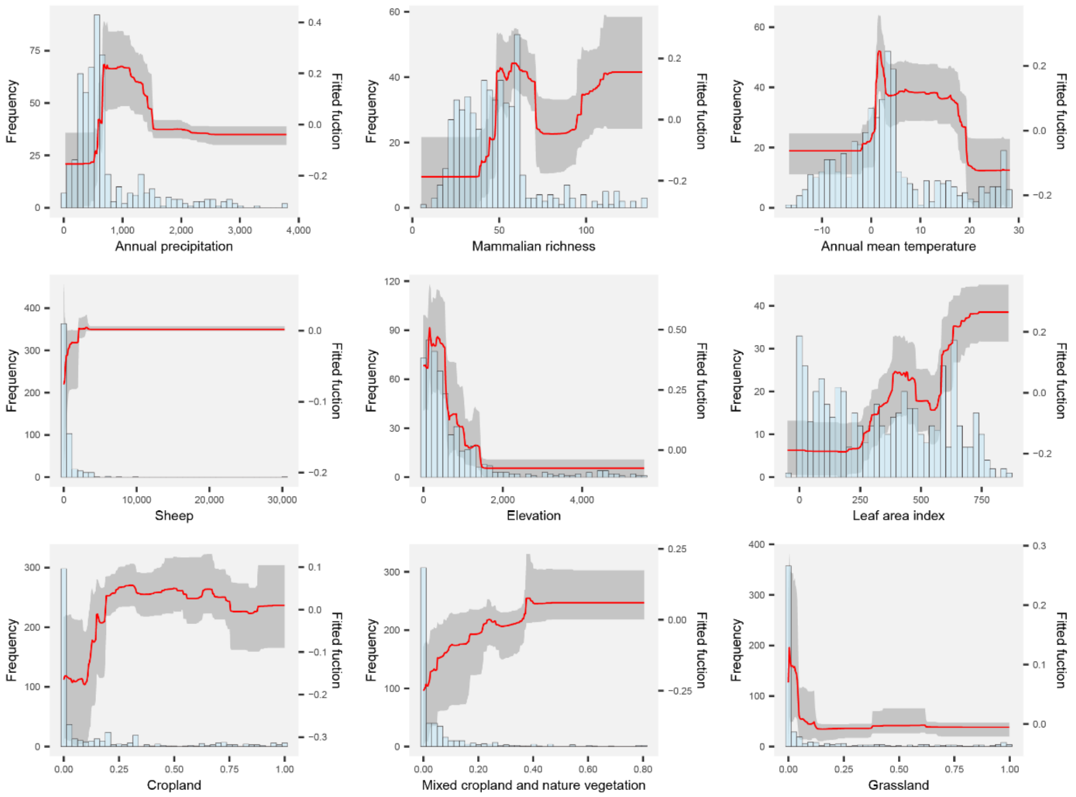

Supplementary Figure 14: Effects of major predictors (relative contributions >3%) for presence of *R. helvetica* based on BRT models. The mean curves (red) and 95% percentiles (gray) show the predicted probability of occurrence at the logit scale. The histograms (blue) show the frequency distributions of the predictors.

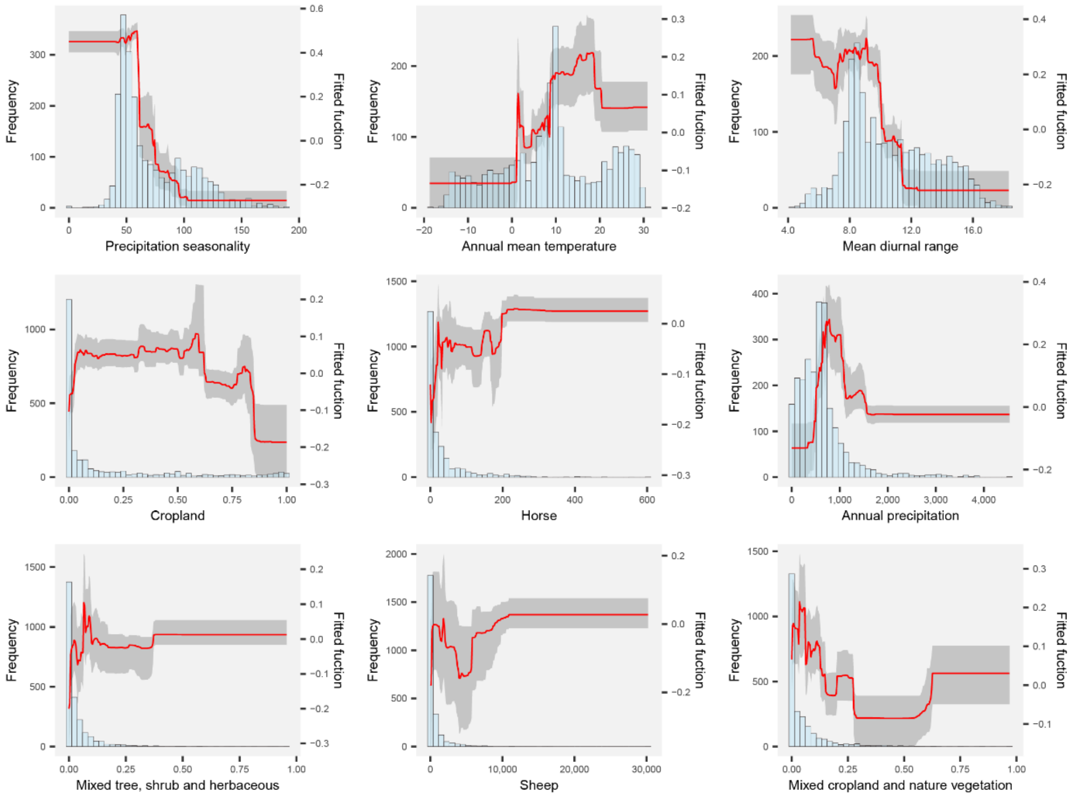

Supplementary Figure 15: Effects of major predictors (relative contributions >3%) for presence of *R. japonica* based on BRT models. The mean curves (red) and 95% percentiles (gray) show the predicted probability of occurrence at the logit scale. The histograms (blue) show the frequency distributions of the predictors.

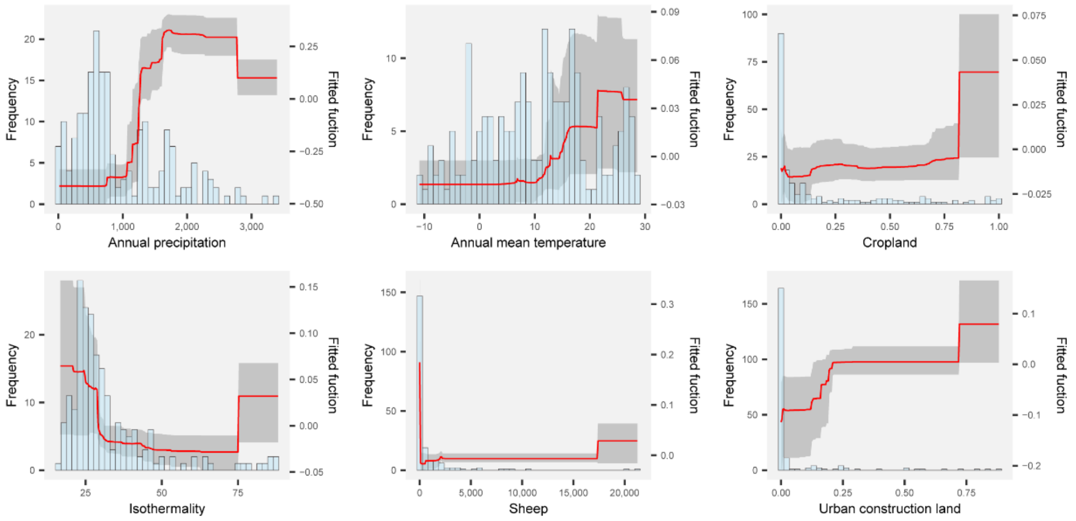

Supplementary Figure 16: Effects of major predictors (relative contributions >3%) for presence of *R. massiliae* based on BRT models. The mean curves (red) and 95% percentiles (gray) show the predicted probability of occurrence at the logit scale. The histograms (blue) show the frequency distributions of the predictors.

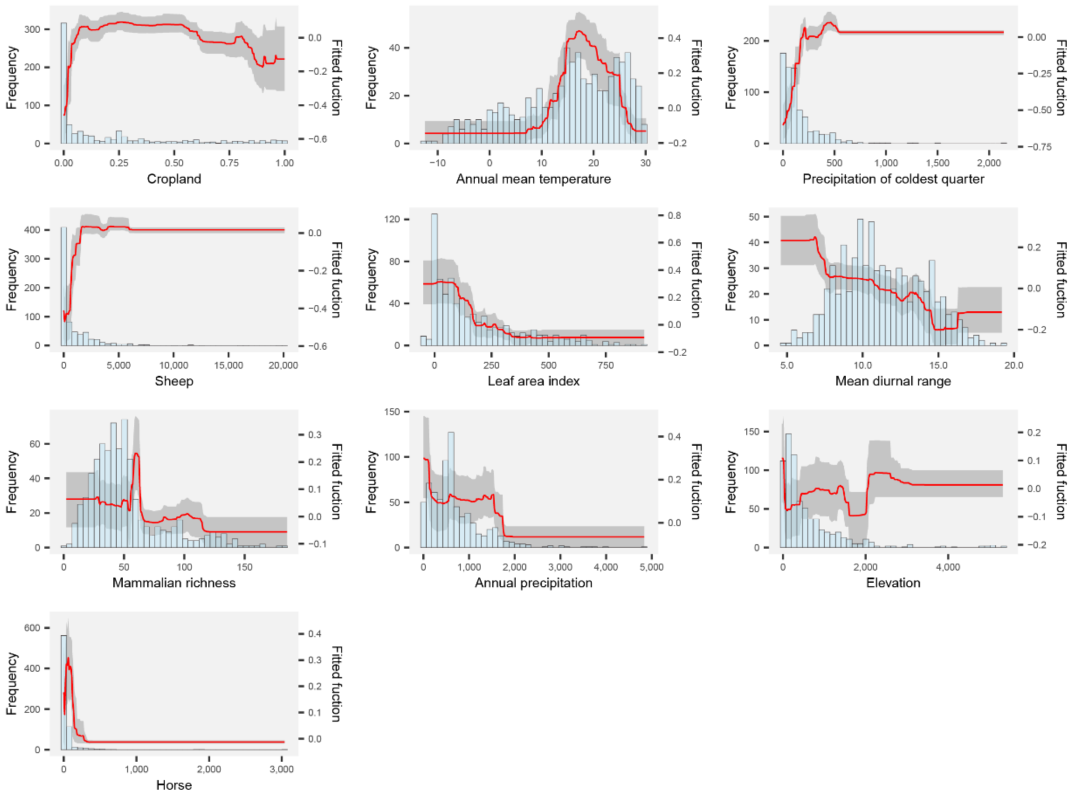

Supplementary Figure 17: Effects of major predictors (relative contributions >3%) for presence of *R. monacensis* based on BRT models. The mean curves (red) and 95% percentiles (gray) show the predicted probability of occurrence at the logit scale. The histograms (blue) show the frequency distributions of the predictors.

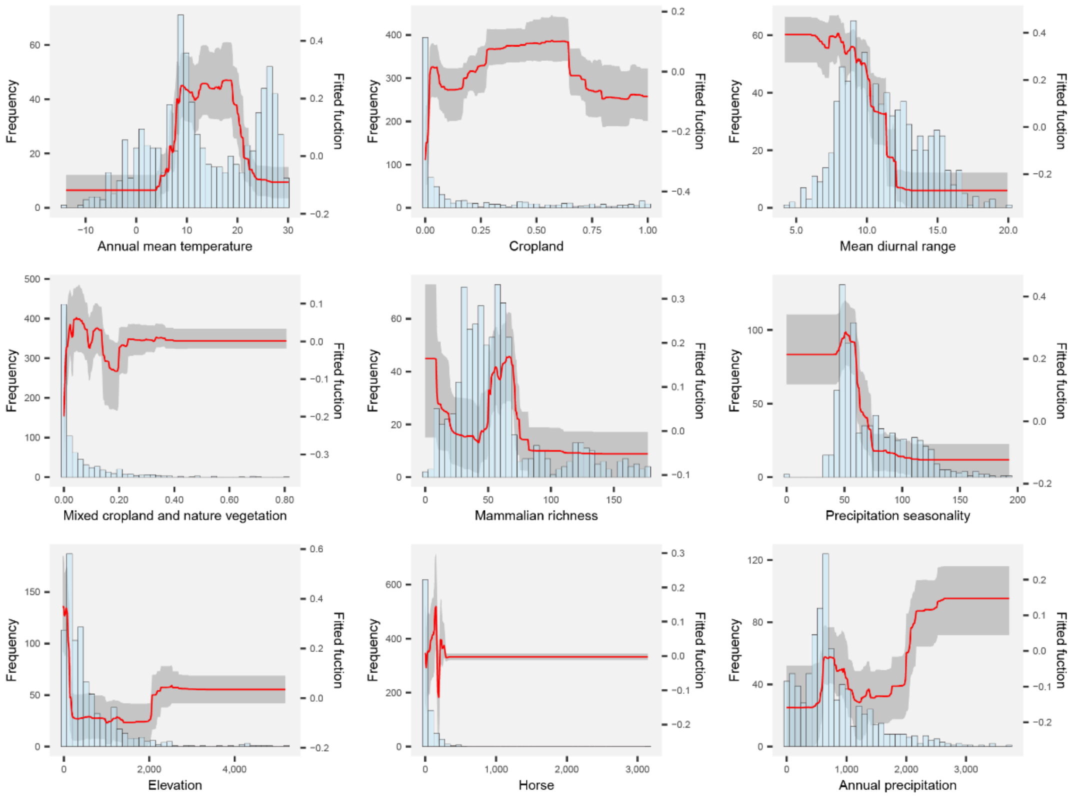

Supplementary Figure 18: Effects of major predictors (relative contributions >3%) for presence of *R. parkeri* based on BRT models. The mean curves (red) and 95% percentiles (gray) show the predicted probability of occurrence at the logit scale. The histograms (blue) show the frequency distributions of the predictors.

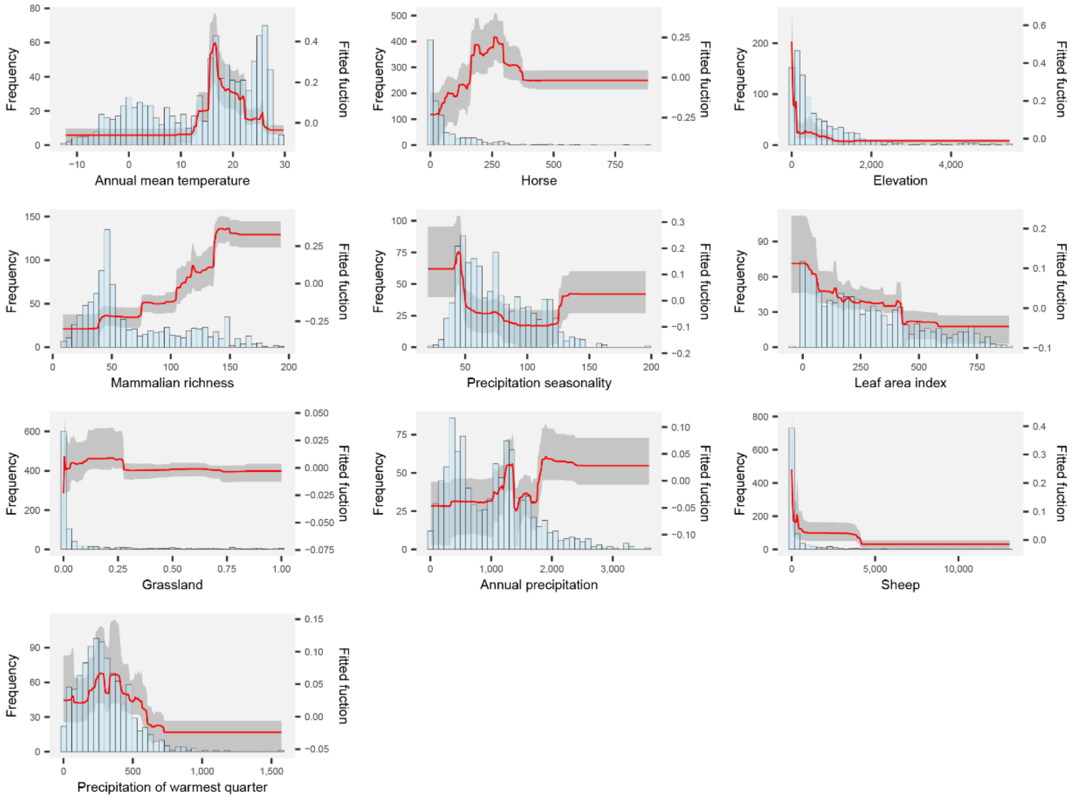

Supplementary Figure 19: Effects of major predictors (relative contributions >3%) for presence of *R. raoultii* based on BRT models. The mean curves (red) and 95% percentiles (gray) show the predicted probability of occurrence at the logit scale. The histograms (blue) show the frequency distributions of the predictors.

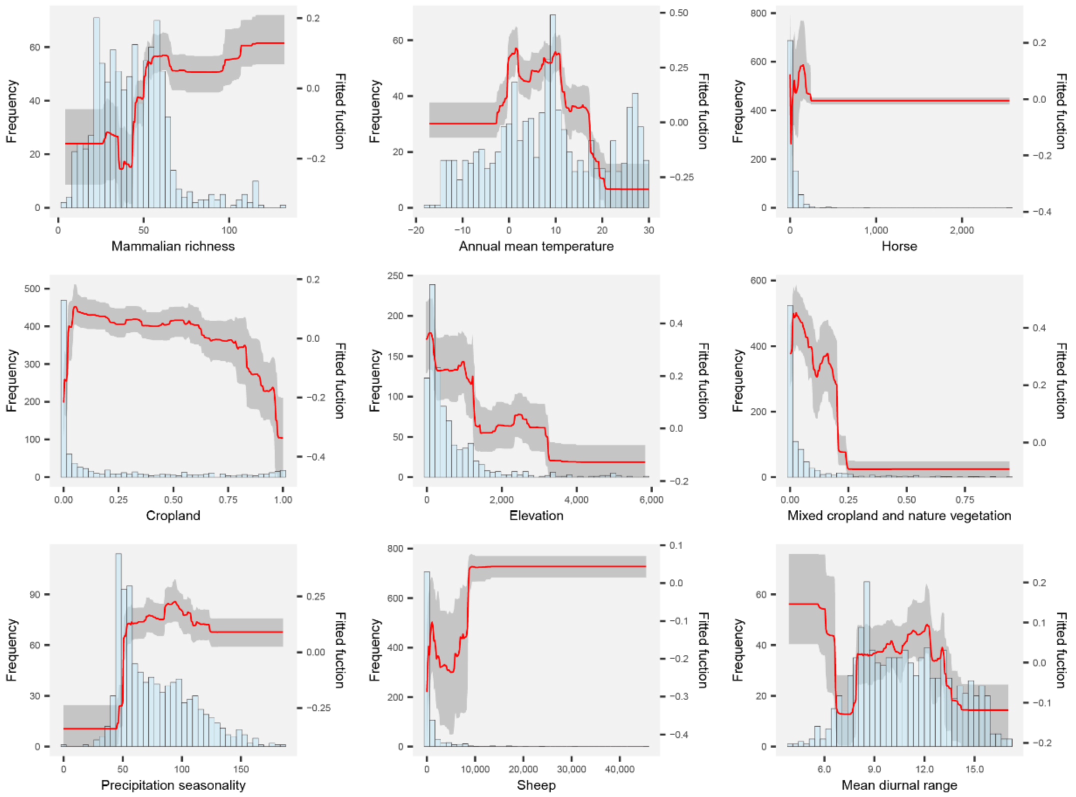

Supplementary Figure 20: Effects of major predictors (relative contributions >3%) for presence of *R. rhipicephali* based on BRT models. The mean curves (red) and 95% percentiles (gray) show the predicted probability of occurrence at the logit scale. The histograms (blue) show the frequency distributions of the predictors.

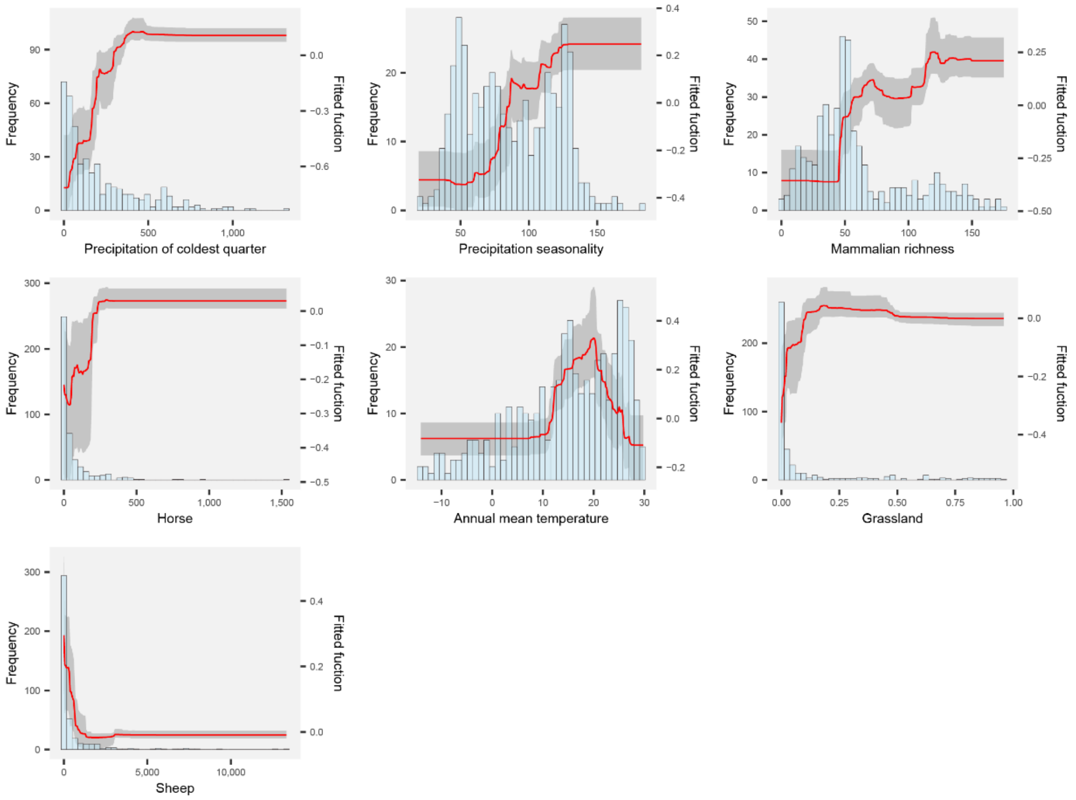

Supplementary Figure 21: Effects of major predictors (relative contributions >3%) for presence of *R. rickettsii* based on BRT models. The mean curves (red) and 95% percentiles (gray) show the predicted probability of occurrence at the logit scale. The histograms (blue) show the frequency distributions of the predictors.

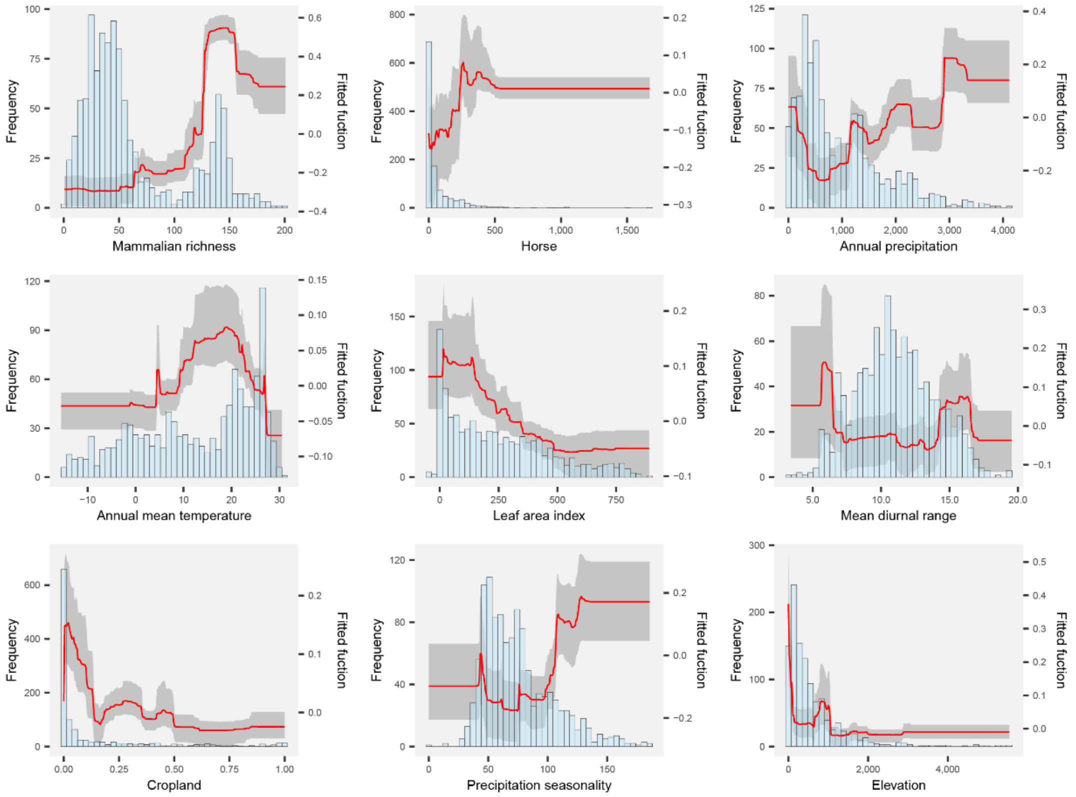

Supplementary Figure 22: Effects of major predictors (relative contributions >3%) for presence of *R. sibirica* based on BRT models. The mean curves (red) and 95% percentiles (gray) show the predicted probability of occurrence at the logit scale. The histograms (blue) show the frequency distributions of the predictors.

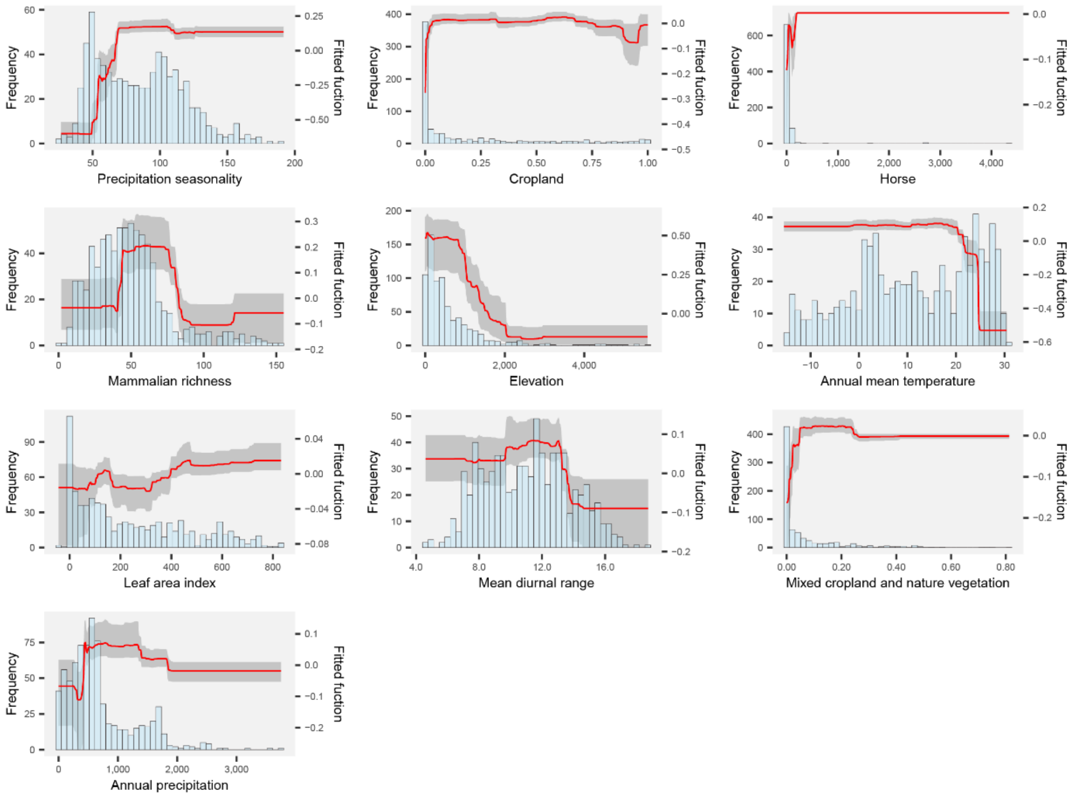

Supplementary Figure 23: Effects of major predictors (relative contributions >3%) for presence of *R. slovaca* based on BRT models. The mean curves (red) and 95% percentiles (gray) show the predicted probability of occurrence at the logit scale. The histograms (blue) show the frequency distributions of the predictors.

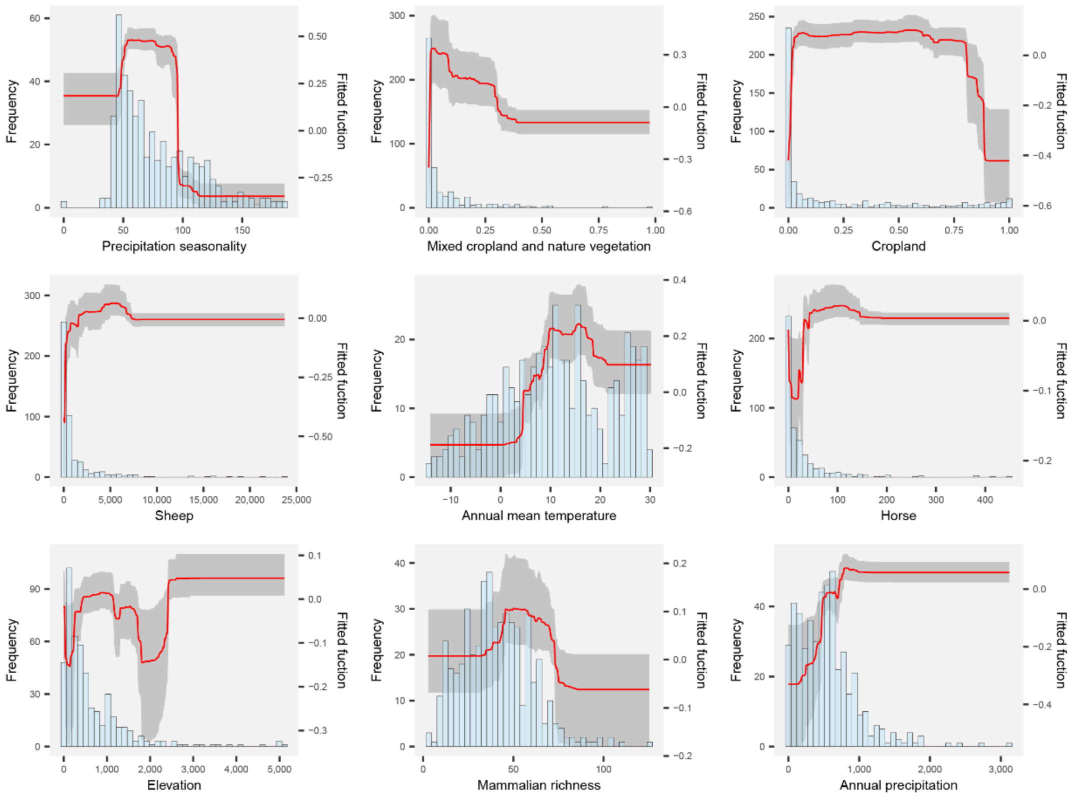

Supplementary Figure 24: Effects of major predictors (relative contributions >3%) for presence of *Candidatus* R. tarasevichiae based on BRT models. The mean curves (red) and 95% percentiles (gray) show the predicted probability of occurrence at the logit scale. The histograms (blue) show the frequency distributions of the predictors.

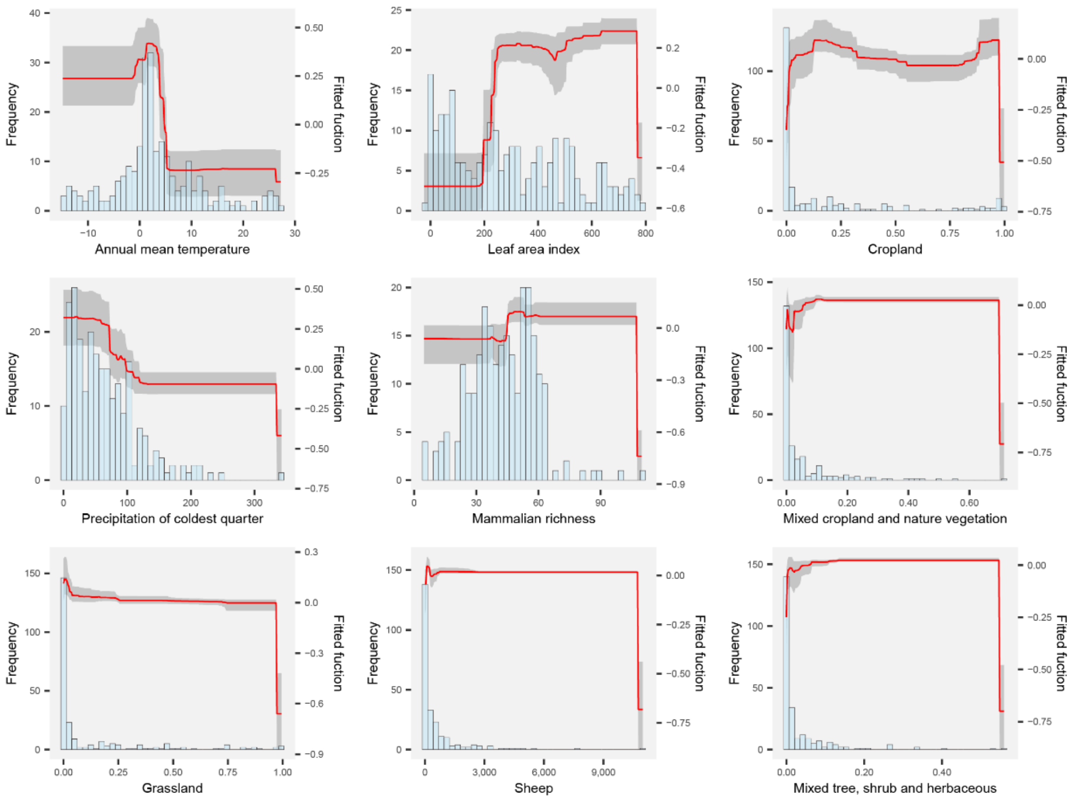

Supplementary figure 25: The global recorded and predicted distributions of *R. aeschlimannii*. (A-C) recorded locations of *R. aeschlimannii* which was detected from arthropod vectors, animal hosts and human beings. (D) Heat map of predicted relative risk distribution based on BRT models about *R. aeschlimannii*. India Peninsula and south Australia were at high risk by predicting.

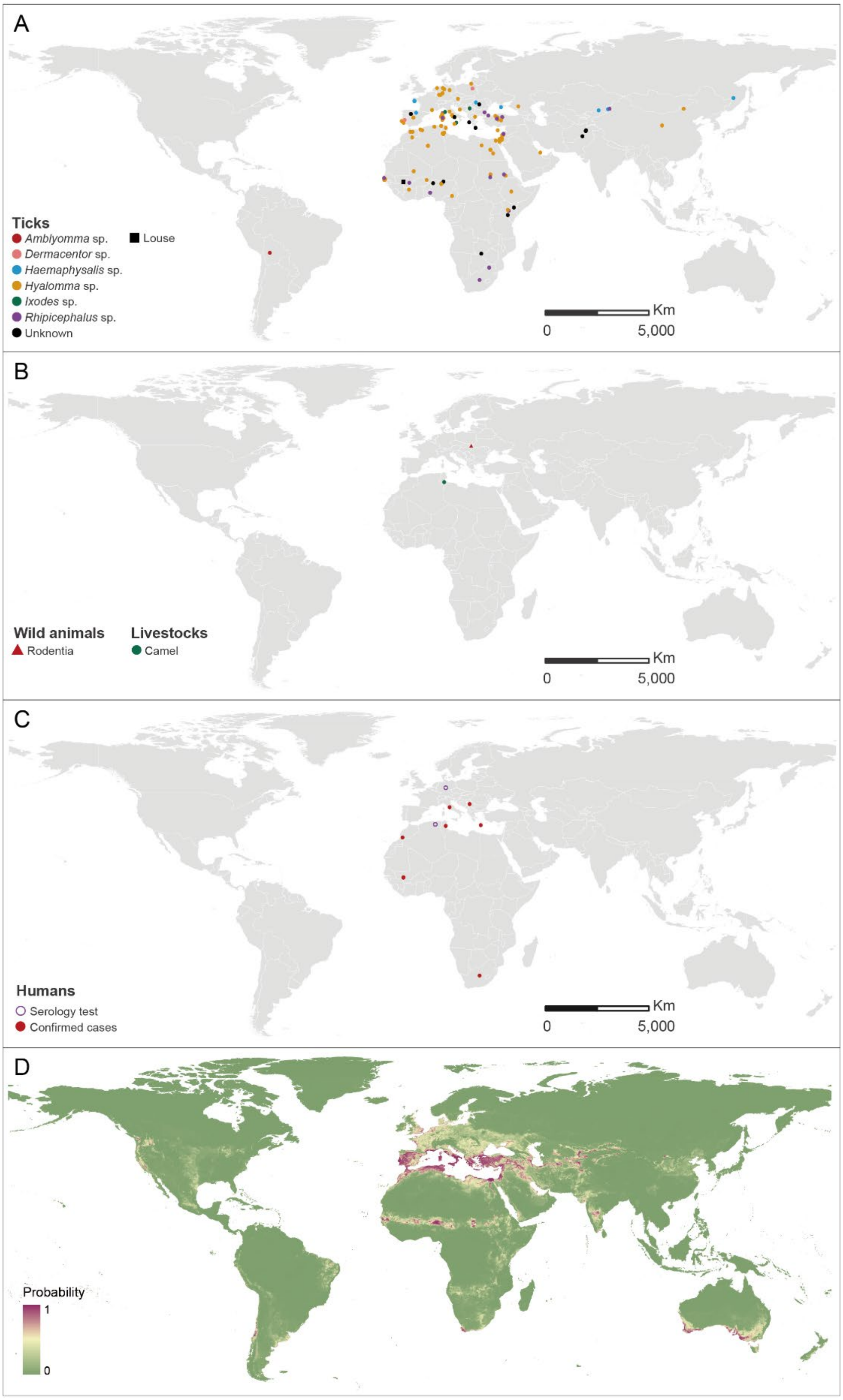

Supplementary figure 26: The global recorded and predicted distributions of *R. africae*. (A-C) recorded locations of *R. africae* which was detected from arthropod vectors, animal hosts and human beings. (D) Heat map of predicted relative risk distribution based on BRT models about *R. africae*. Global tropical regions were at high risk.

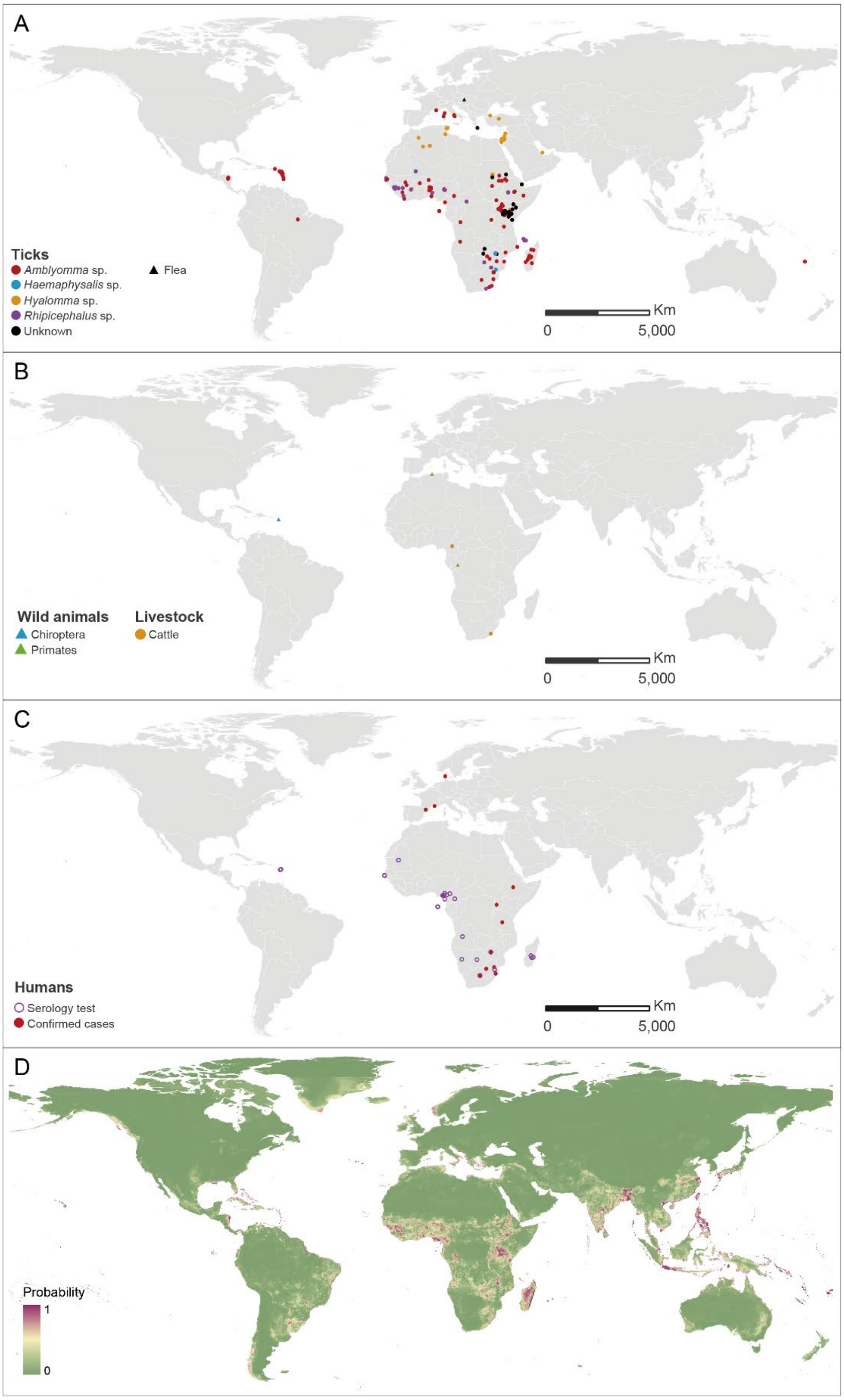

Supplementary figure 27: The global recorded and predicted distributions of *R. amblyommii*. (A-C) recorded locations of *R. amblyommii* which was detected from arthropod vectors, animal hosts and human beings. (D) Heat map of predicted relative risk distribution based on BRT models about *R. amblyommii*. New regions the prediction map found included central Africa and Southeast Asia.

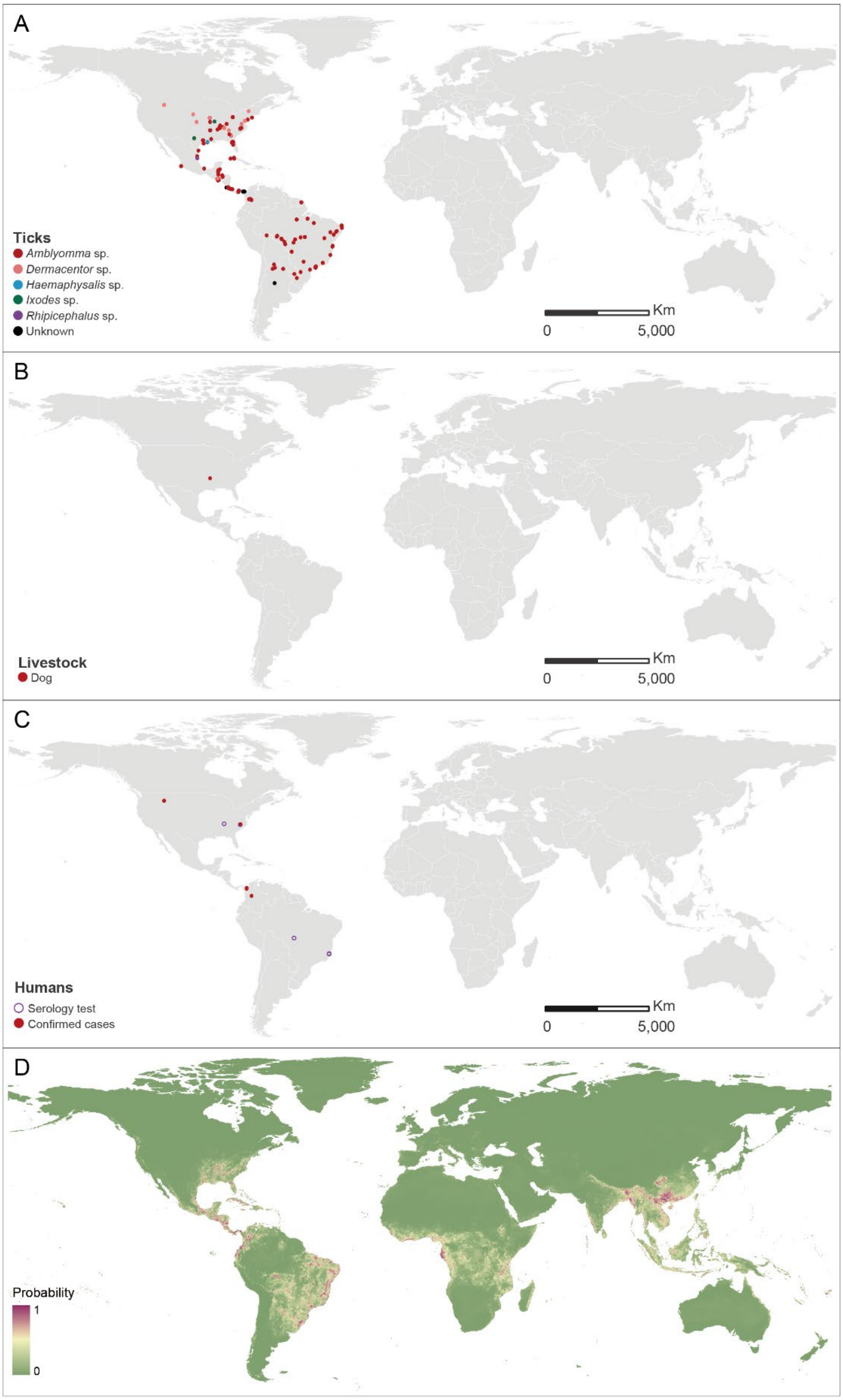

Supplementary figure 28: The global recorded and predicted distributions of *R. conorii*. (A-C) recorded locations of *R. conorii* which was detected from arthropod vectors, animal hosts and human beings. (D) Heat map of predicted relative risk distribution based on BRT models about *R. conorii*. New regions the prediction map found included southern South America and southern Australia.

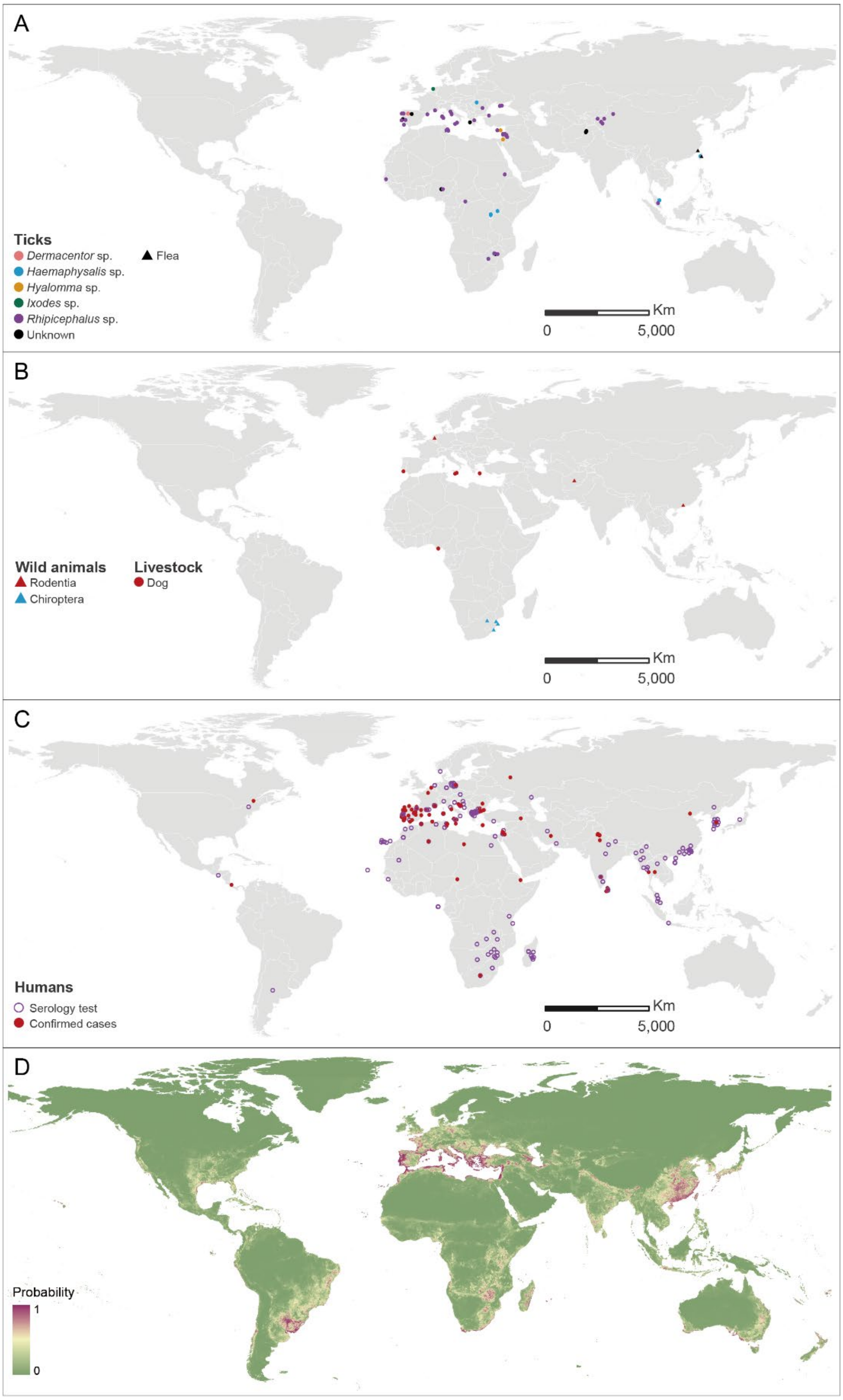

Supplementary figure 29: The global recorded and predicted distributions of *R. felis*. (A-C) recorded locations of *R. felis* which was detected from arthropod vectors, animal hosts and human beings. (D) Heat map of predicted relative risk distribution based on BRT models about *R. felis*. Every continent was at high risk.

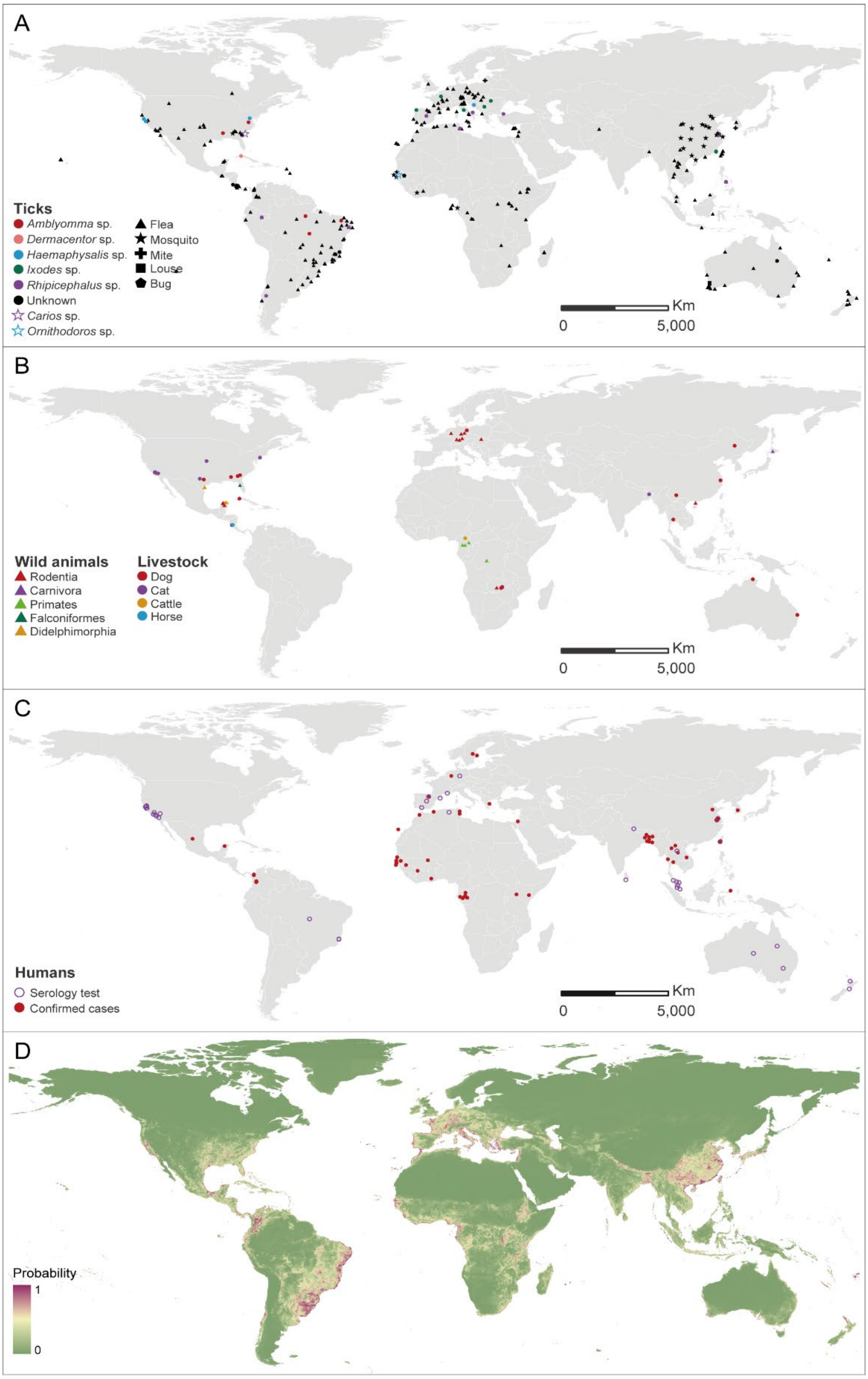

Supplementary figure 30: The global recorded and predicted distributions of *R. heilongjiangensis*. (A-C) recorded locations of *R. heilongjiangensis* which was detected from arthropod vectors, animal hosts and human beings. (D) Heat map of predicted relative risk distribution based on BRT models about *R. heilongjiangensis*. The prediction map found that most area of Europe and some parts of south America were at high risk.

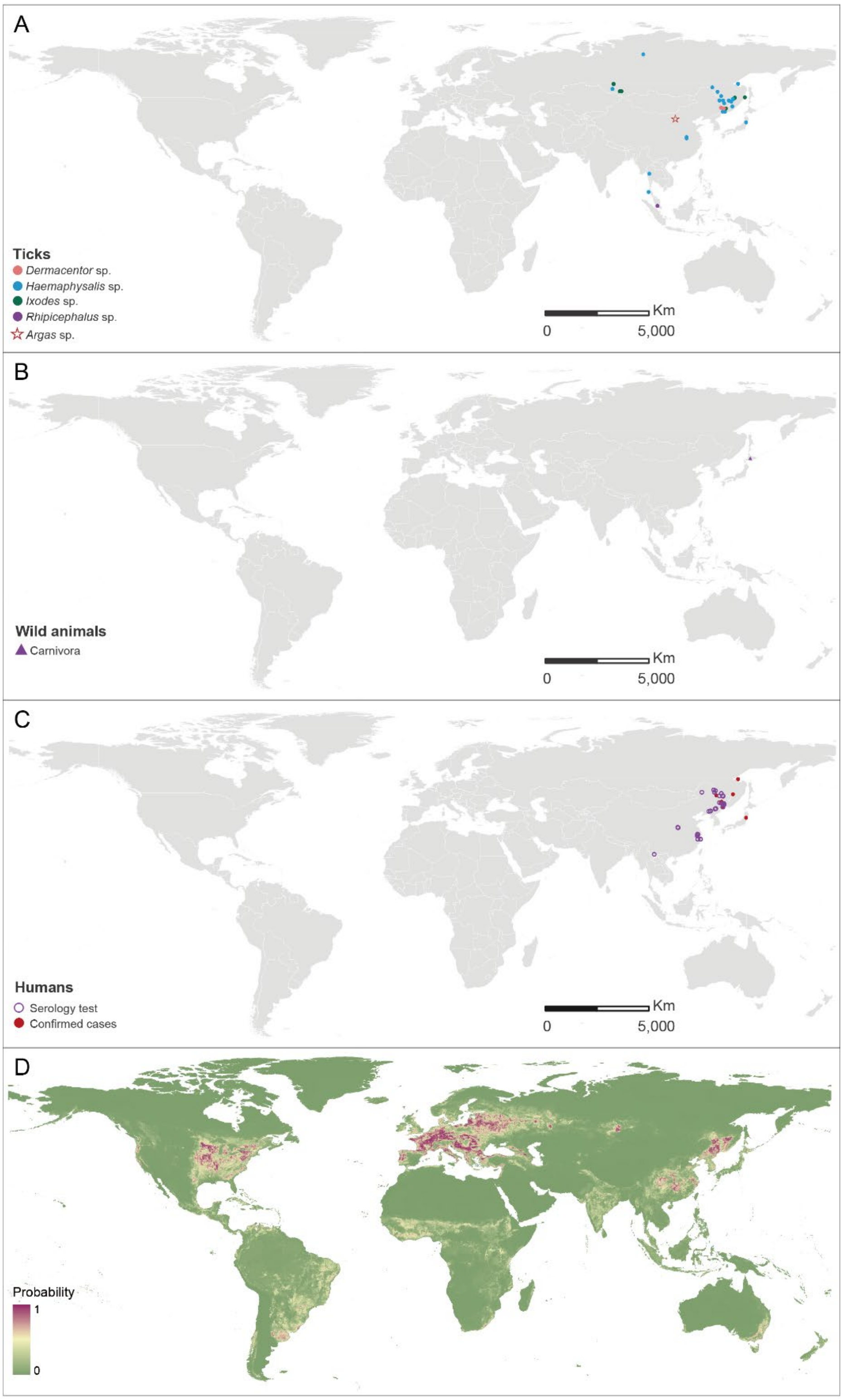

Supplementary figure 31: The global recorded and predicted distributions of *R. helvetica*. (A-C) recorded locations of *R. helvetica* which was detected from arthropod vectors, animal hosts and human beings. (D) Heat map of predicted relative risk distribution based on BRT models about *R. helvetica*.

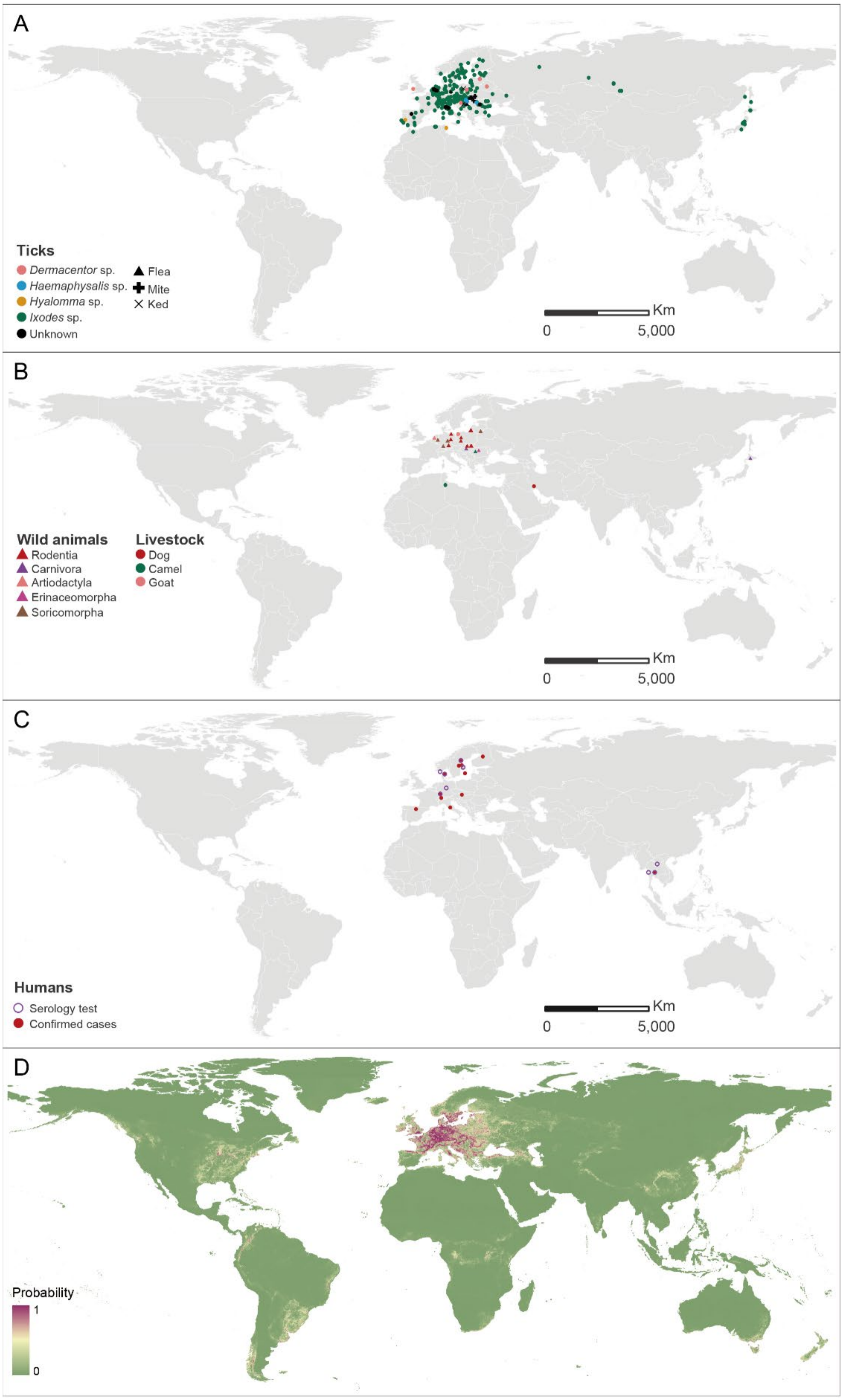

Supplementary figure 32: The global recorded and predicted distributions of *R. japonica*. (A-C) recorded locations of *R. japonica* which was detected from arthropod vectors, animal hosts and human beings. (D) Heat map of predicted relative risk distribution based on BRT models about *R. japonica*. The region of east and southeast Asia was at high risk.

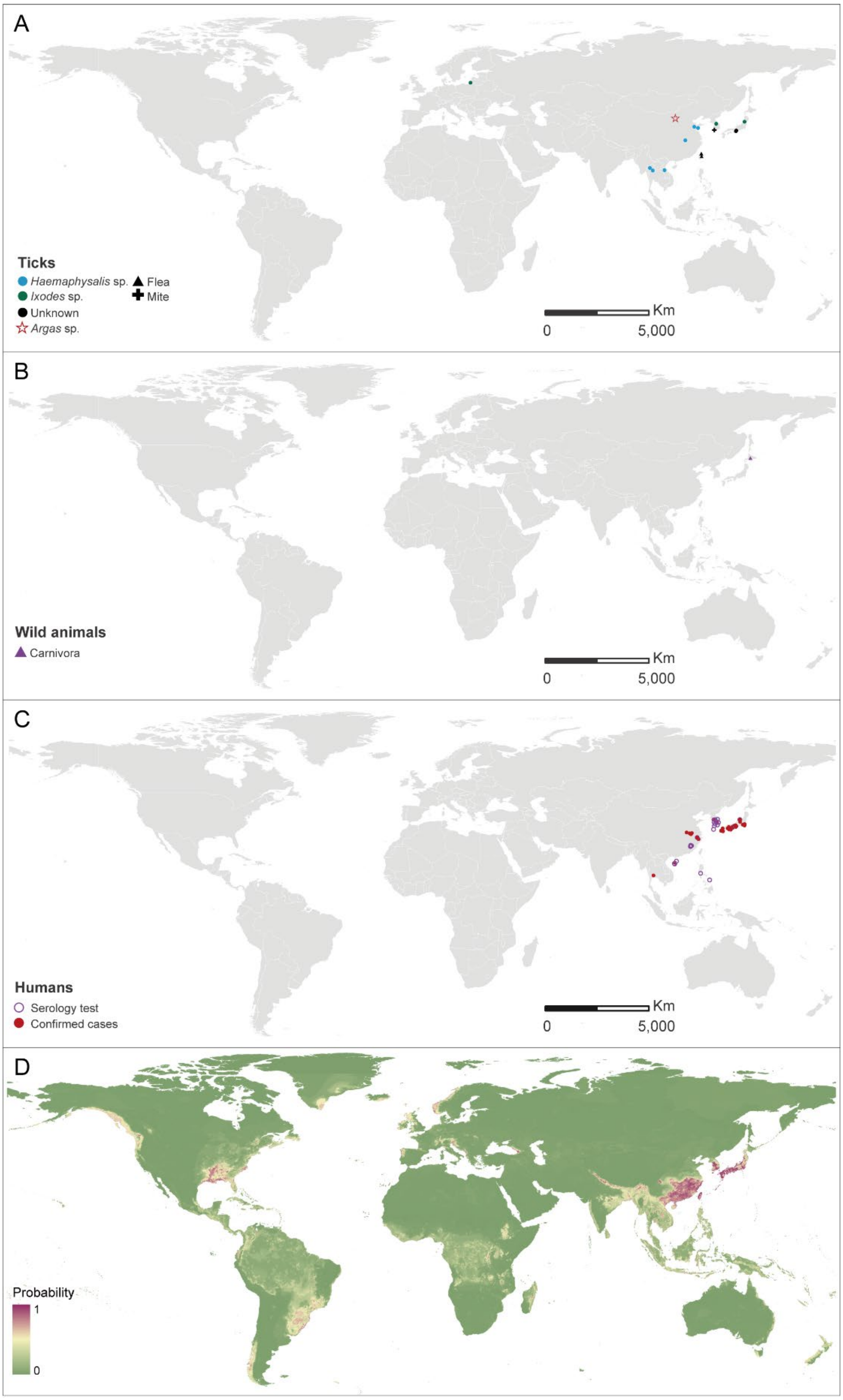

Supplementary figure 33: The global recorded and predicted distributions of *R. massiliae*. (A-C) recorded locations of *R. massiliae* which was detected from arthropod vectors, animal hosts and human beings. (D) Heat map of predicted relative risk distribution based on BRT models about *R. massiliae*. The new regions suitable for existence of *R. massiliae* by predicting included southern India and Australia.

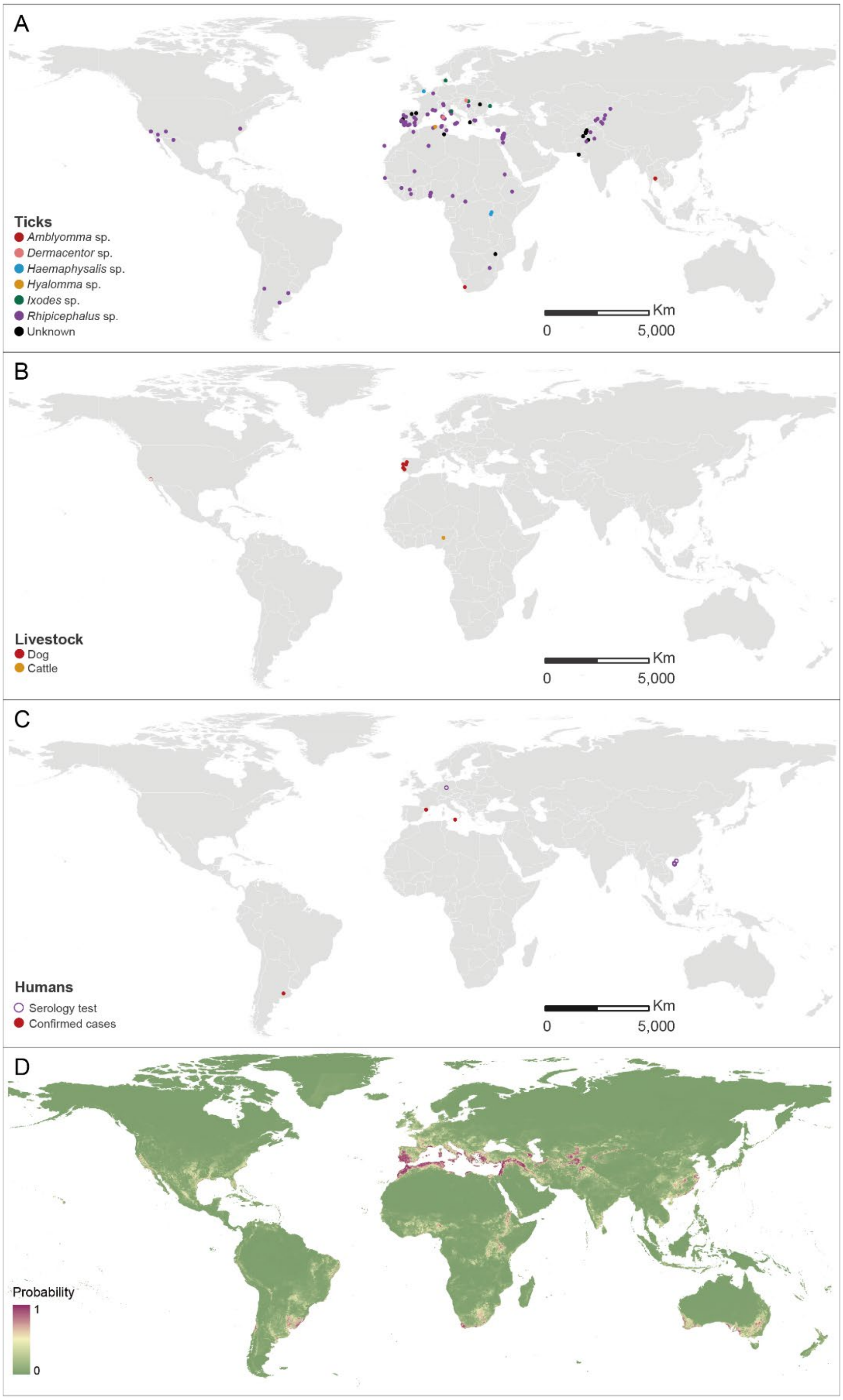

Supplementary figure 34: The global recorded and predicted distributions of *R. monacensis*. (A-C) recorded locations of *R. monacensis* which was detected from arthropod vectors, animal hosts and human beings. (D) Heat map of predicted relative risk distribution based on BRT models about *R. monacensis*.

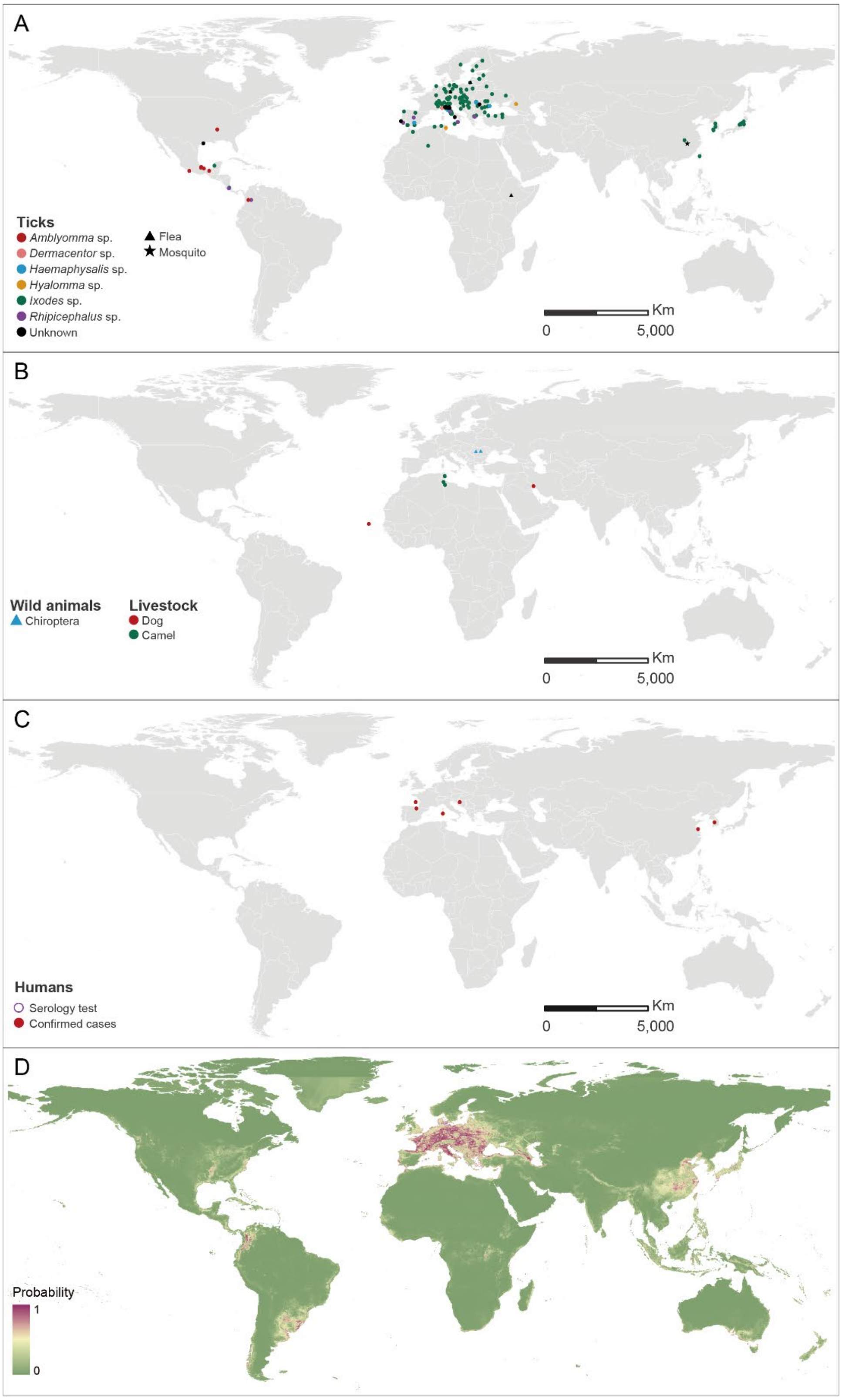

Supplementary figure 35: The global recorded and predicted distributions of *R. parkeri*. (A-C) recorded locations of *R. parkeri* which was detected from arthropod vectors, animal hosts and human beings. (D) Heat map of predicted relative risk distribution based on BRT models about *R. parkeri*. Some parts of southeast Asia were newly predicted at high risk.

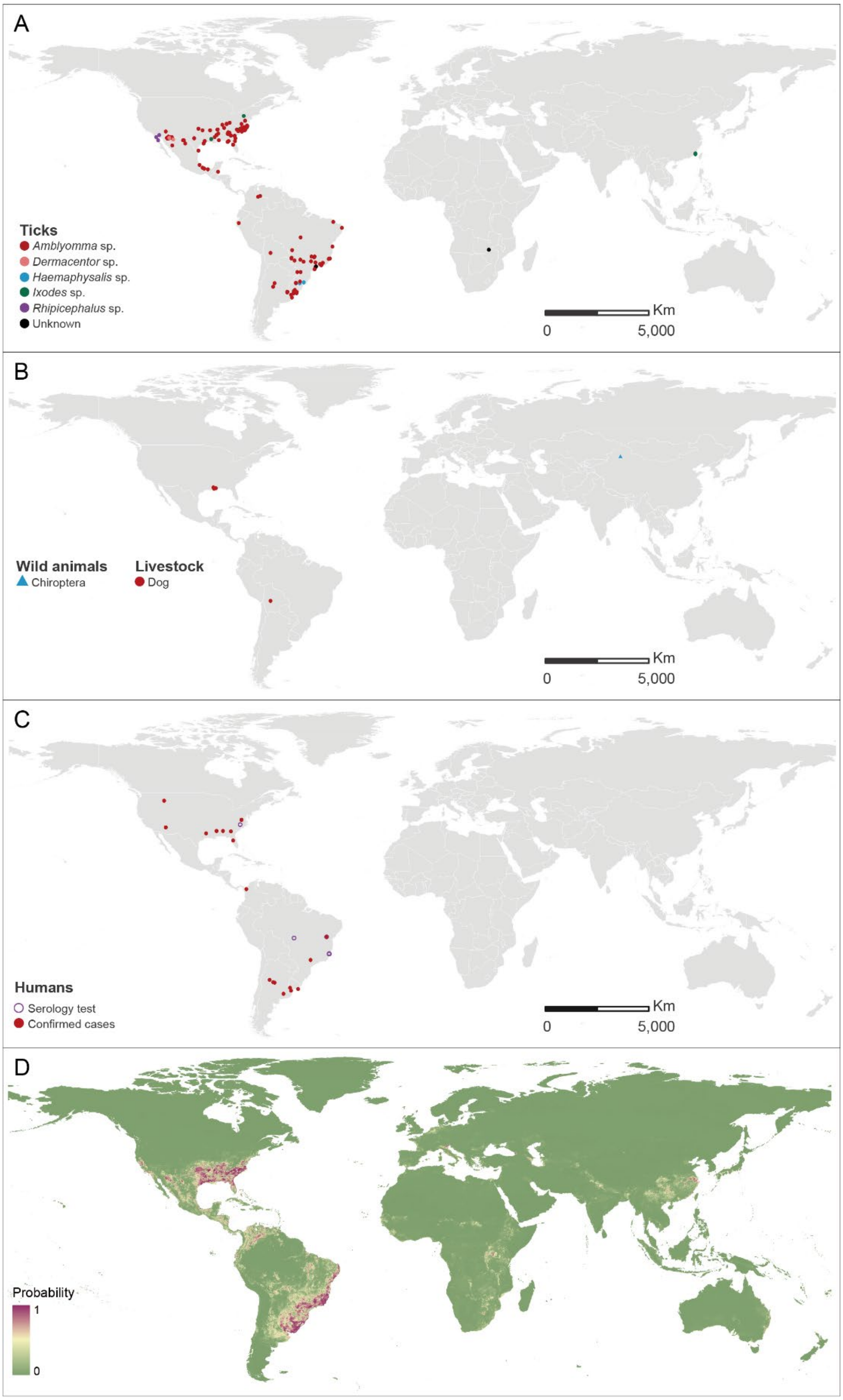

Supplementary figure 36: The global recorded and predicted distributions of *R. raoultii*. (A-C) recorded locations of *R. raoultii* which was detected from arthropod vectors, animal hosts and human beings. (D) Heat map of predicted relative risk distribution based on BRT models about *R. raoultii*. Western-central area of North America were newly predicted at high risk.

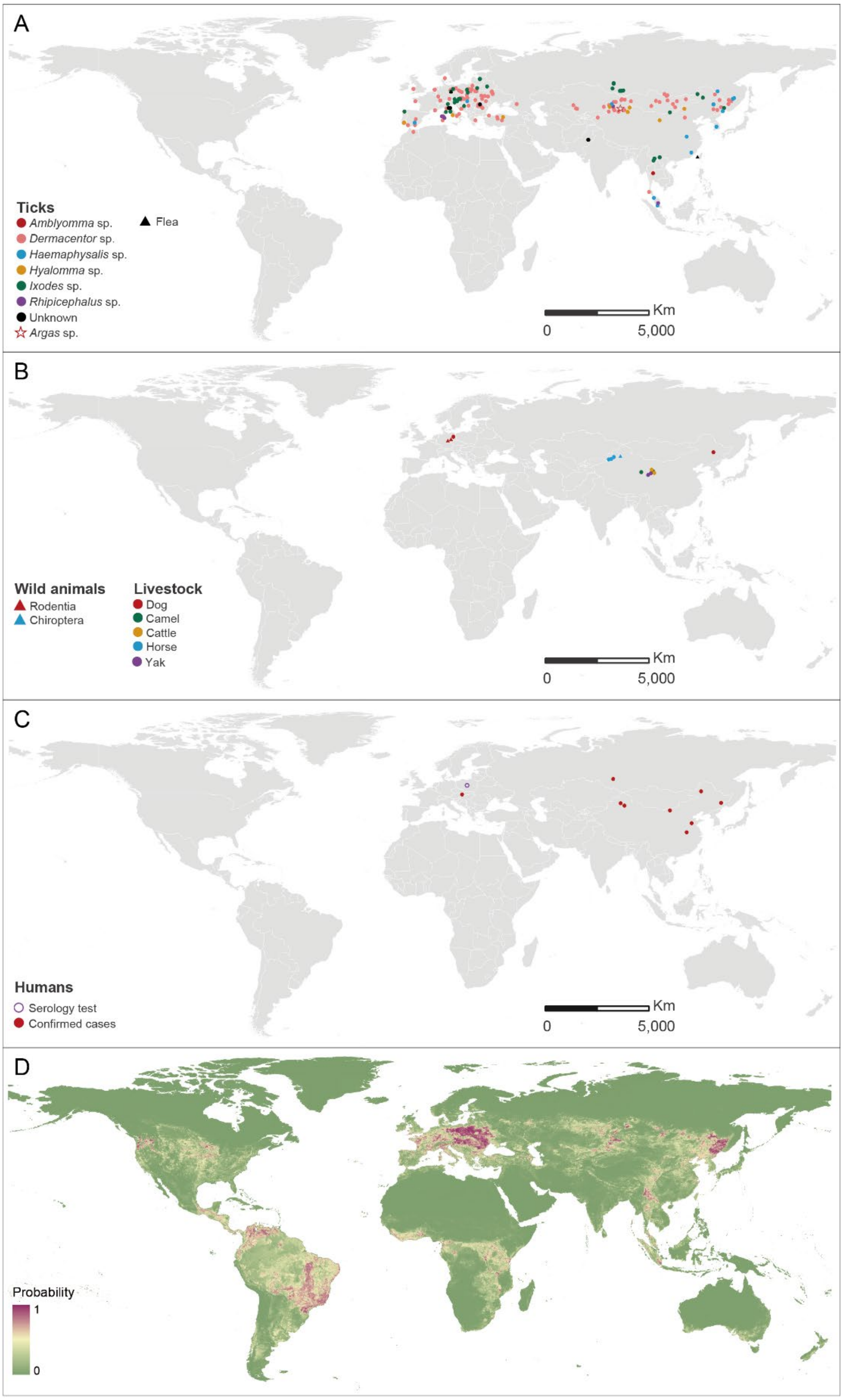

Supplementary figure 37: The global recorded and predicted distributions of *R. rhipicephali*. (A-C) recorded locations of *R. rhipicephali* which was detected from arthropod vectors, animal hosts and human beings. (D) Heat map of predicted relative risk distribution based on BRT models about *R. rhipicephali*. A coastal region of southwest South America was newly predicted with a high suitability of *R. rhipicephali*.

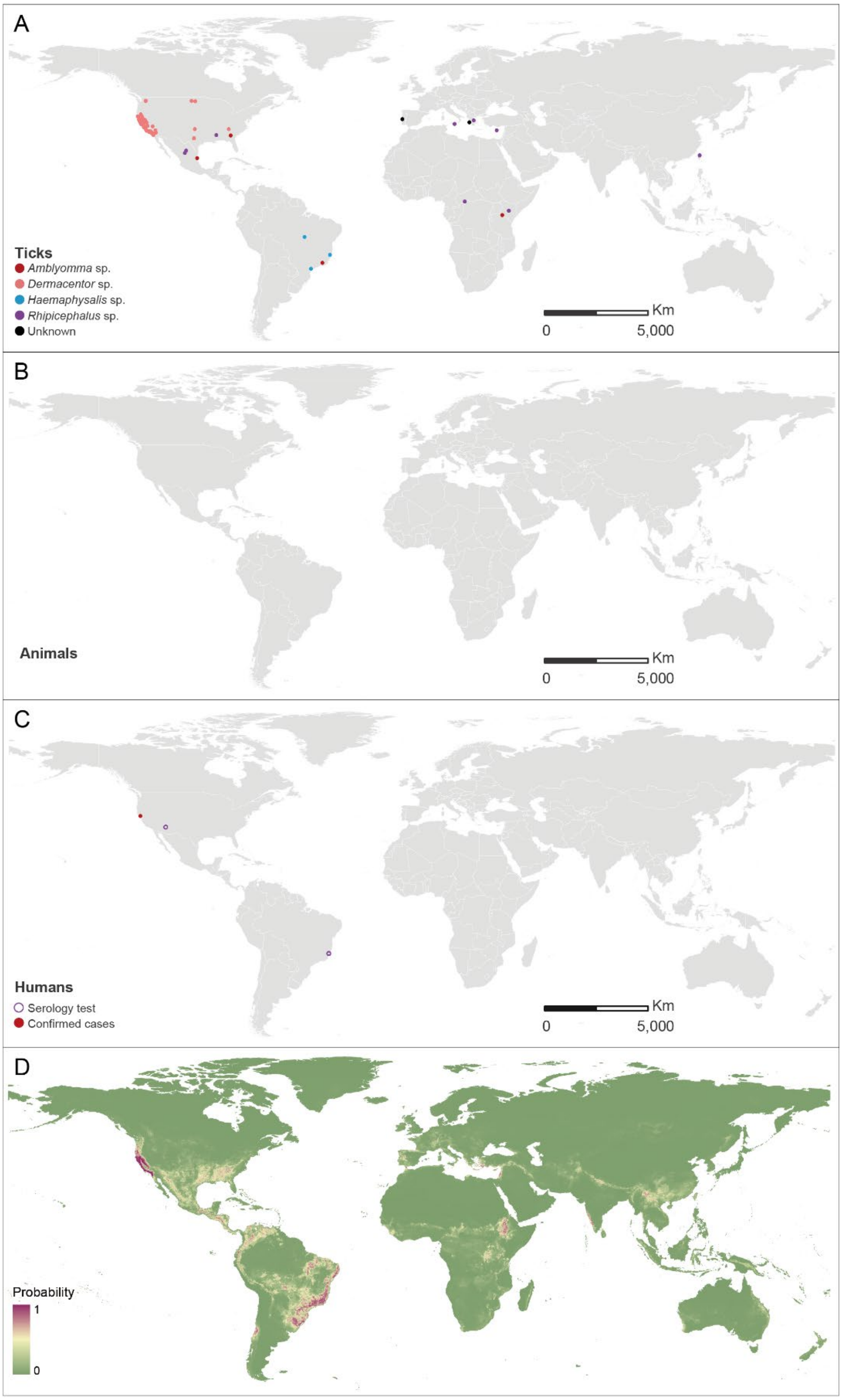

Supplementary figure 38: The global recorded and predicted distributions of *R. rickettsii*. (A-C) recorded locations of *R. rickettsii* which was detected from arthropod vectors, animal hosts and human beings. (D) Heat map of predicted relative risk distribution based on BRT models about *R. rickettsii*. The prediction map indicated that Western-central area of North America, Central America, Southern South America, southern Africa, European area, southeastern Asia were at high risk.

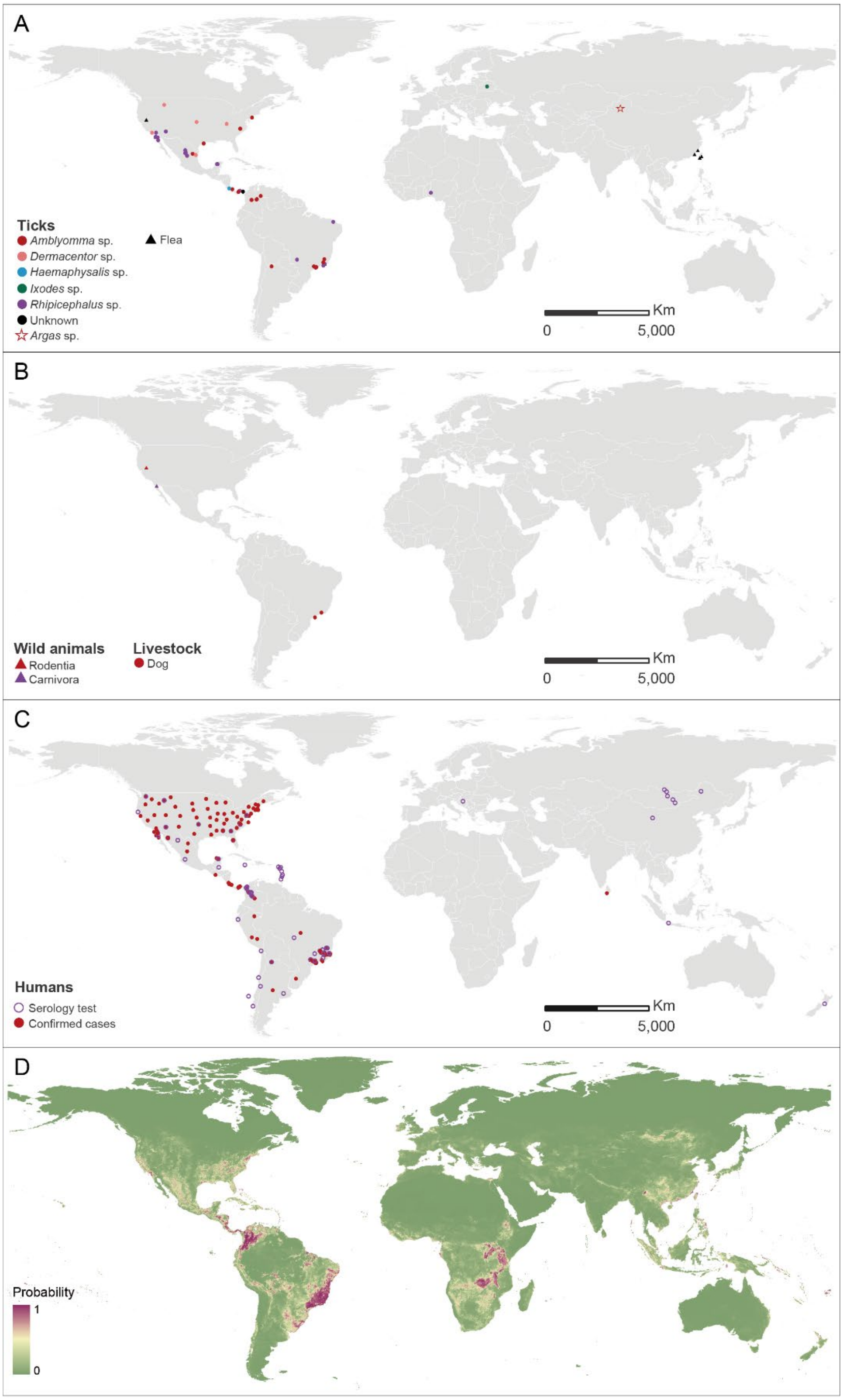

Supplementary figure 39: The global recorded and predicted distributions of *R. sibirica*. (A-C) recorded locations of *R. sibirica* which was detected from arthropod vectors, animal hosts and human beings. (D) Heat map of predicted relative risk distribution based on BRT models about *R. sibirica*. The prediction map indicated that Western-central area of North America, Southern South America, southern Africa, Mediterranean area, eastern and southern Asia were at high risk.

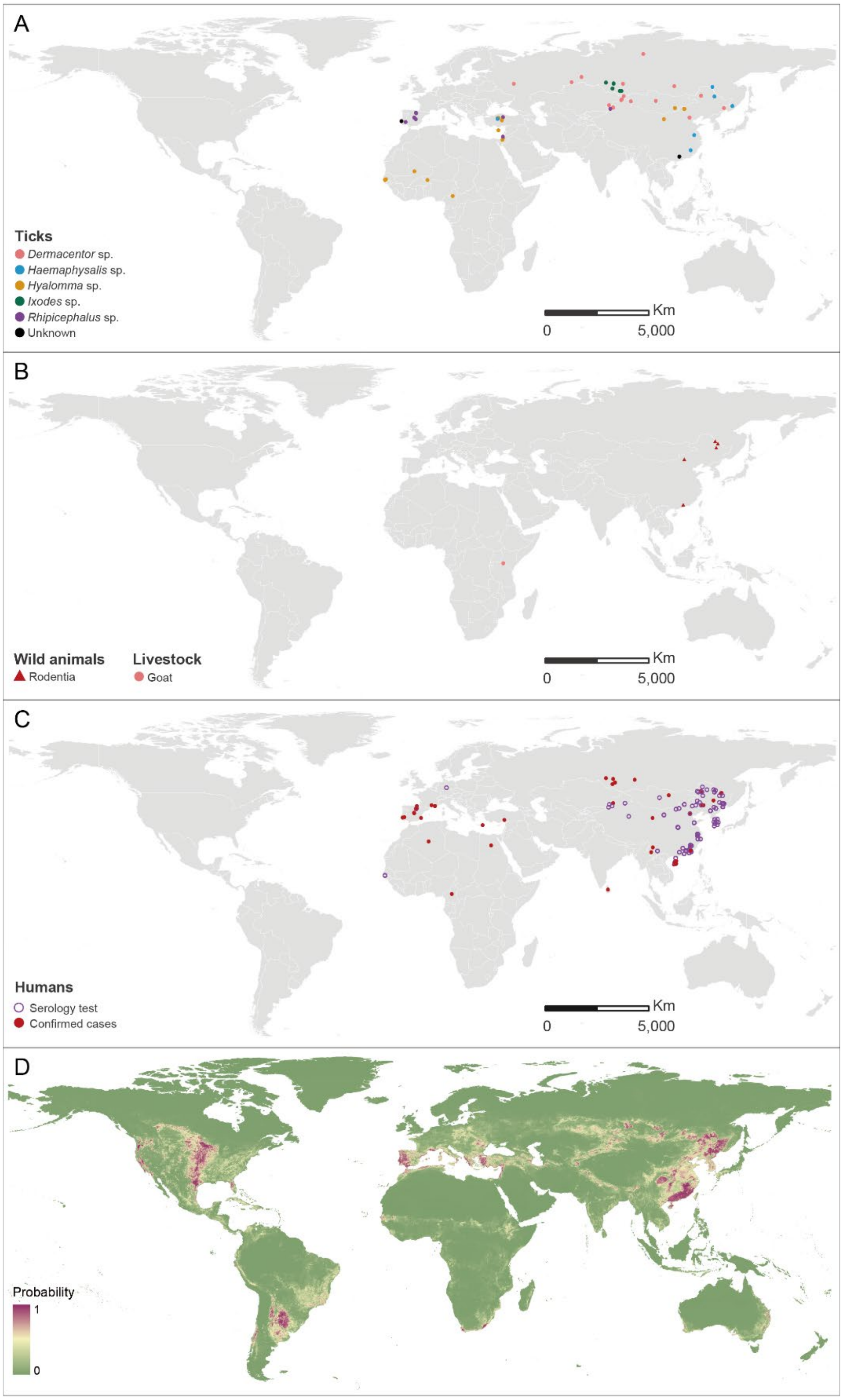

Supplementary figure 40: The global recorded and predicted distributions of *R. slovaca*. (A-C) recorded locations of *R. slovaca* which was detected from arthropod vectors, animal hosts and human beings. (D) Heat map of predicted relative risk distribution based on BRT models about *R. slovaca*. Many regions were newly at high risk by predicting including middle America, east coast and southern South America, southern Africa, east Asian and south Australia.

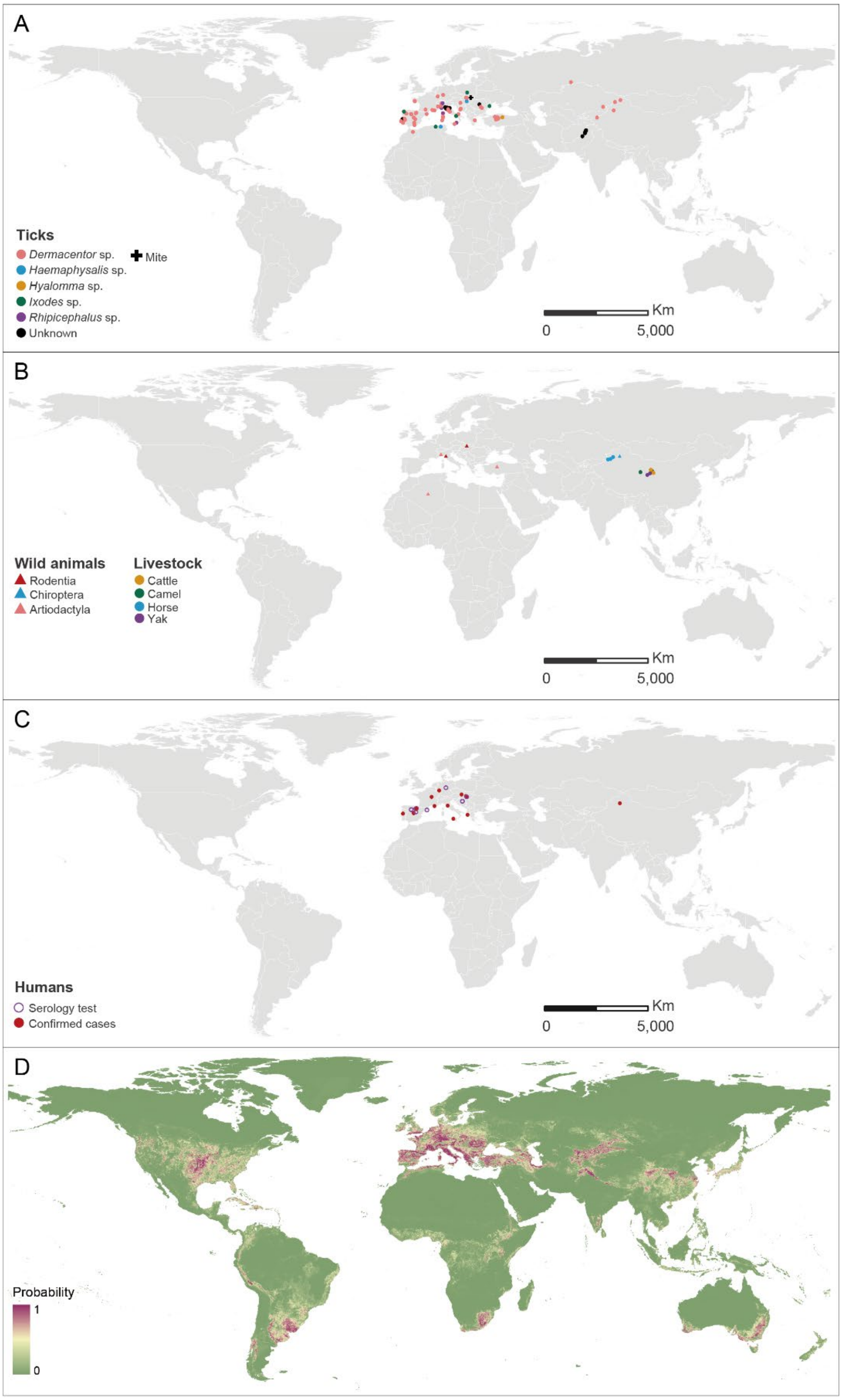

Supplementary figure 41: The global recorded and predicted distributions of *Candidatus* R. tarasevichiae. (A-C) recorded locations of *Candidatus* R. tarasevichiae which was detected from arthropod vectors, animal hosts and human beings. (D) Heat map of predicted relative risk distribution based on BRT models about *Candidatus* R. tarasevichiae. Central North America and some parts of eastern Asia were newly predicted at high risk.

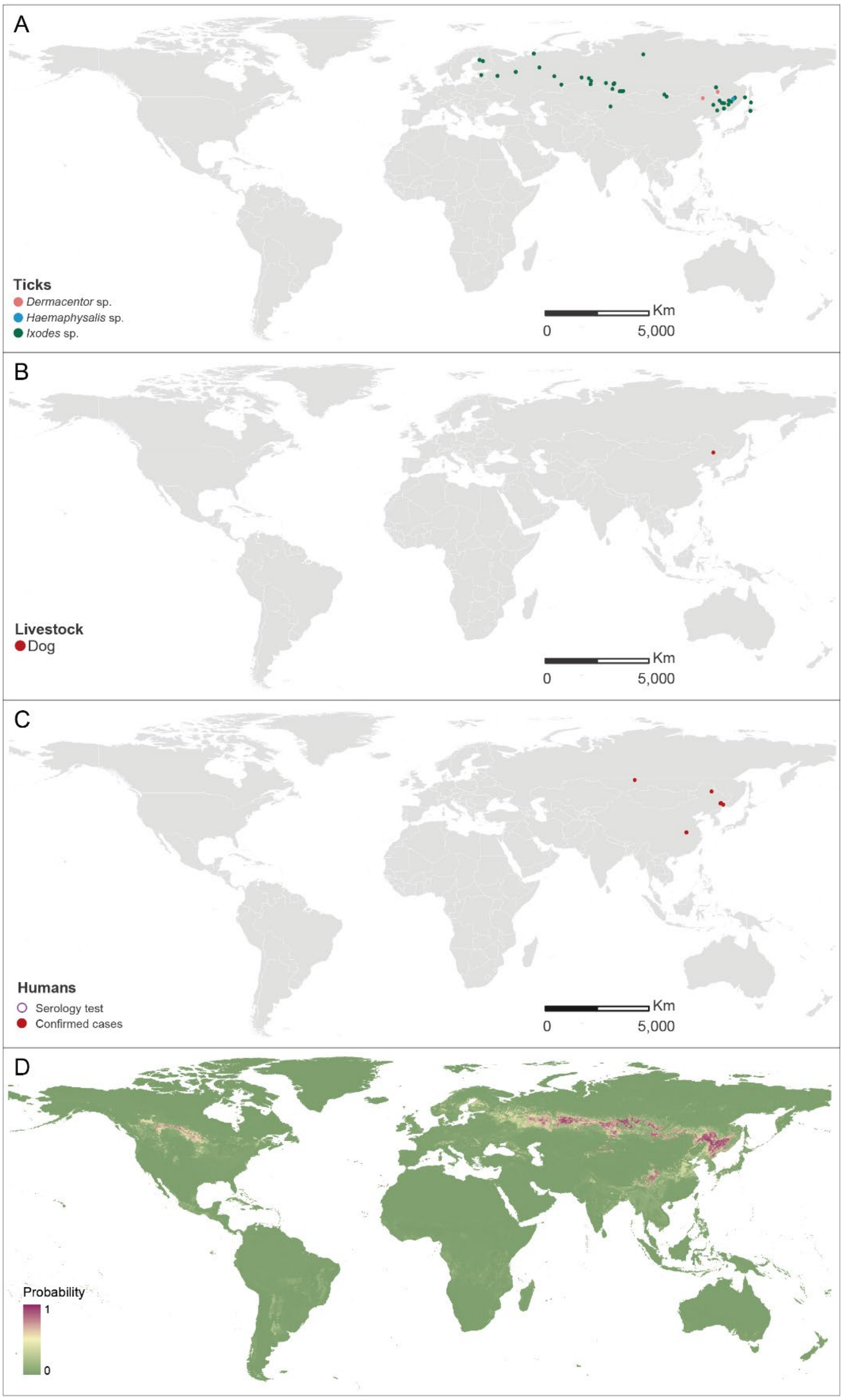

Supplementary figure 42: The global recorded distributions of six species rickettsiae. (A) *R. akari*, (B) *R. australis*, (C) *R. honei*, (D) *R. montanensis*, (E) *R. philipii*, (F) *R. asembonensis*.

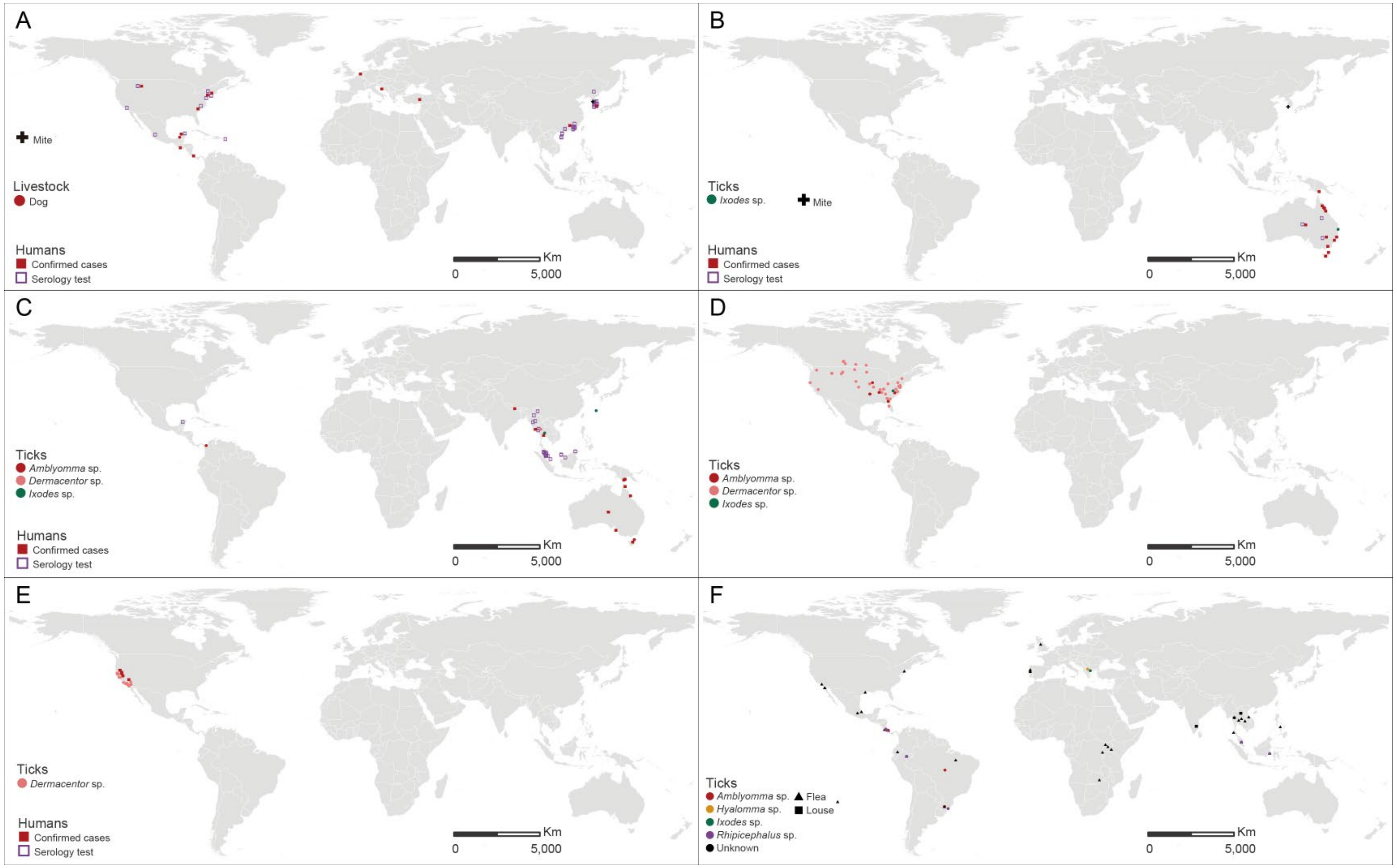

Supplementary figure 43: The global recorded distributions of other six species rickettsiae. (A) *R. buchneri*, (B) *R. cooleyi*, (C) *R.* endosymbiont *of I. scapularis*, (D) *R. gravesii*, (E) *R. hoogstraalii*, (F) *R. peacockii*.

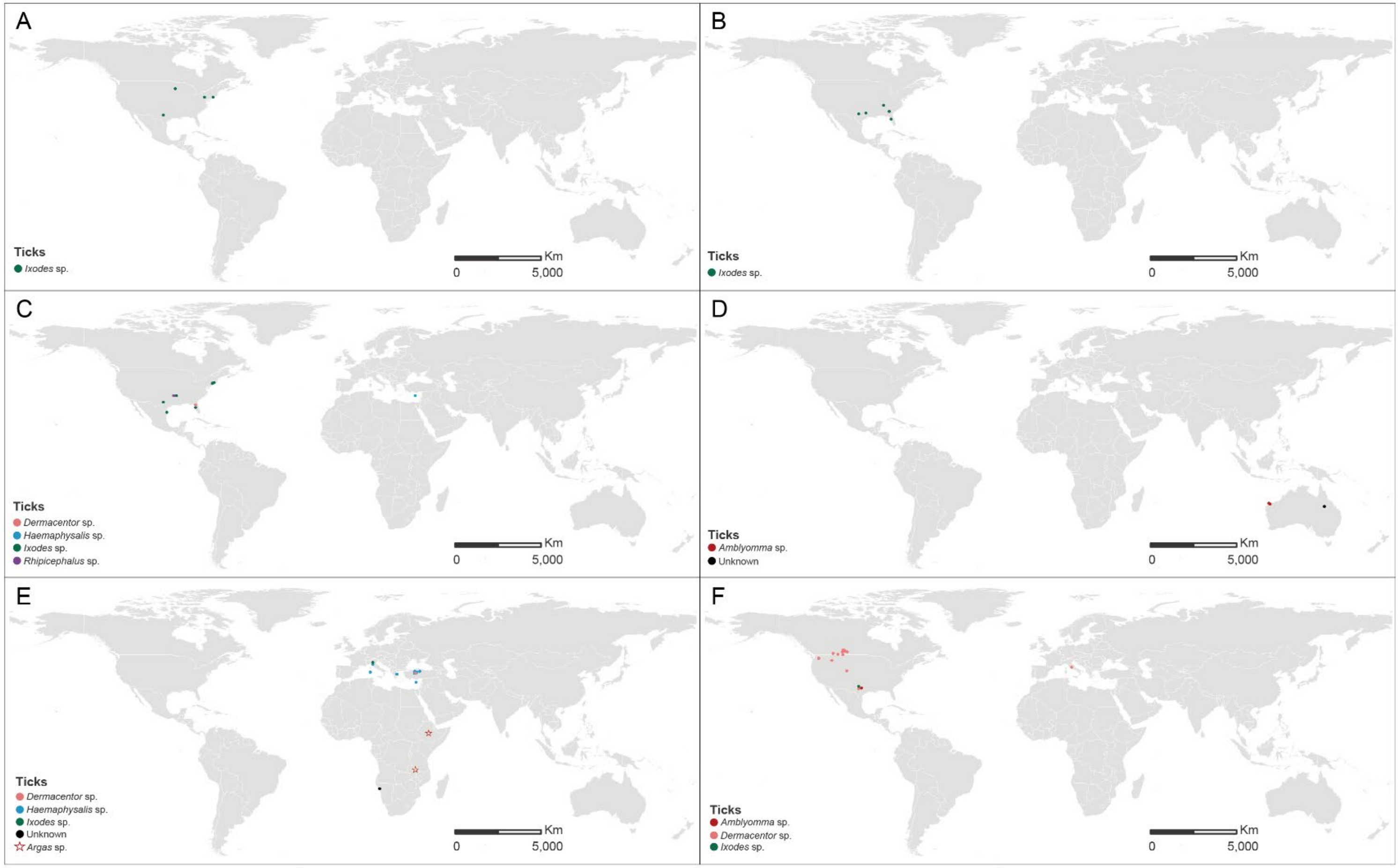

Supplementary figure 44: The global recorded distributions of three species rickettsiae. (A) *R. tamurae*, (B) *R. thailandii*, (C) *R. vini*.

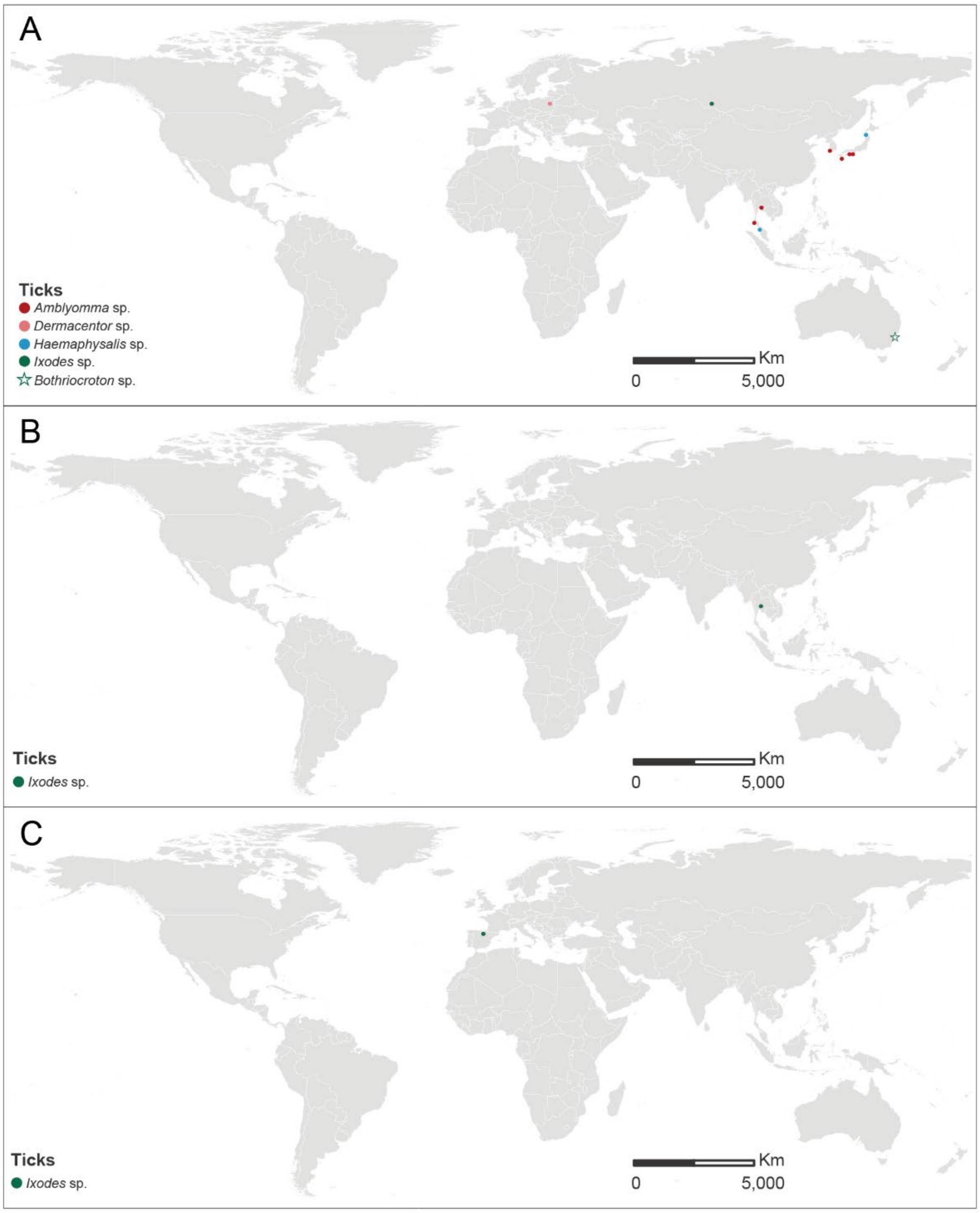

Supplementary figure 45: The global recorded distributions of six species *Candidatus* rickettsiae. (A) *Candidatus R.* rioja, (B) *Candidatus R.* kellyi, (C) *Candidatus R.* xinyangensis, (D) *Candidatus* R. andeanae, (E) *Candidatus R.* barbariae, (F) *Candidatus R.* colombianensi.

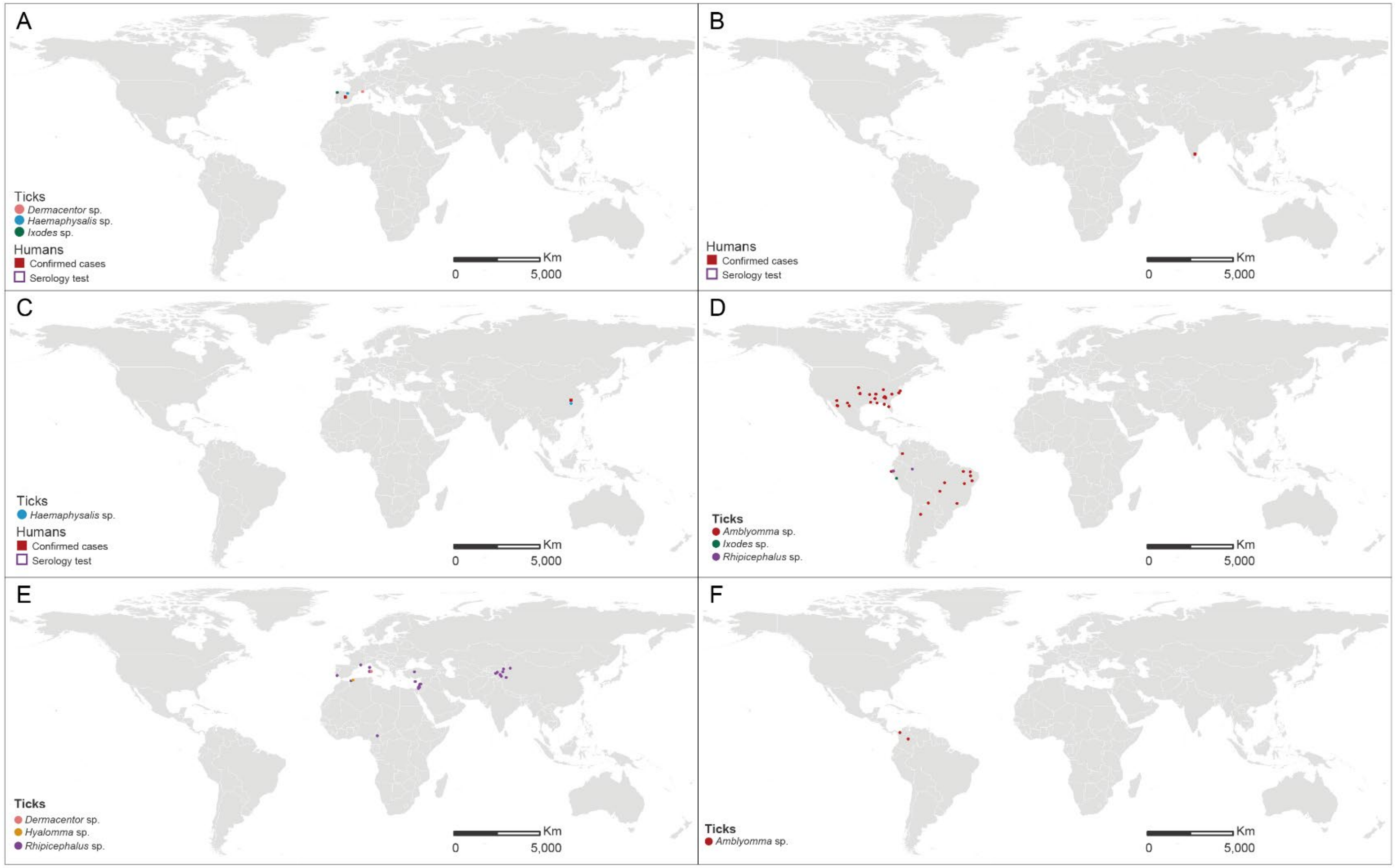

Supplementary figure 46: The global recorded distributions of other six species *Candidatus* rickettsiae. (A) *Candidatus R.* goldwasserii, (B) *Candidatus R.* jingxinensis, (C) *Candidatus R.* longicornii, (D) *Candidatus R.* mendelii, (E) *Candidatus R.* moyalensis, (F) *Candidatus R.* paranaensis.

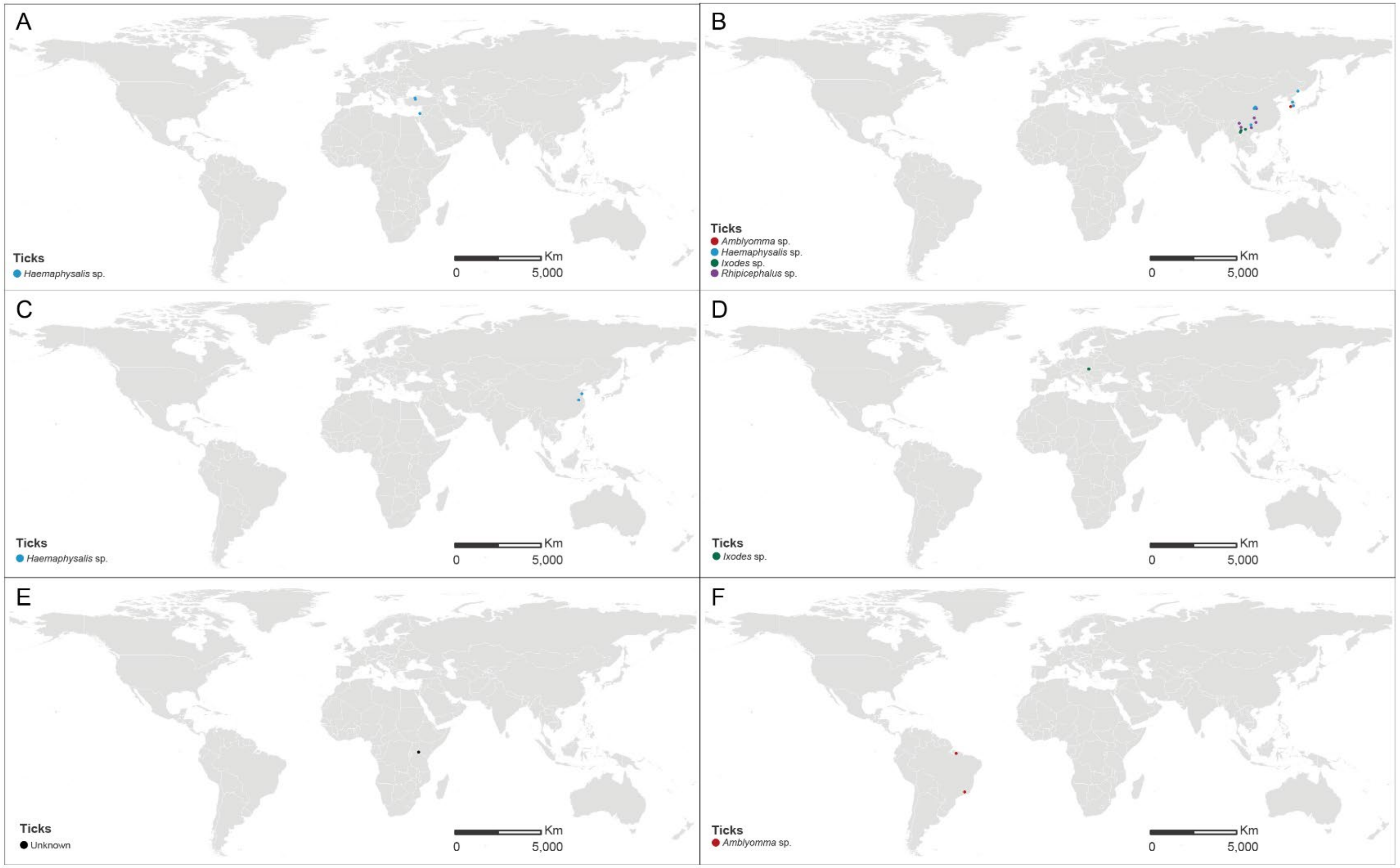

Supplementary figure 47: The global recorded distributions of four species *Candidatus* rickettsiae. (A) *Candidatus R.* principis, (B) *Candidatus R.* rara, (C) *Candidatus R.* senegalensis, (D) *Candidatus R.* uralica.

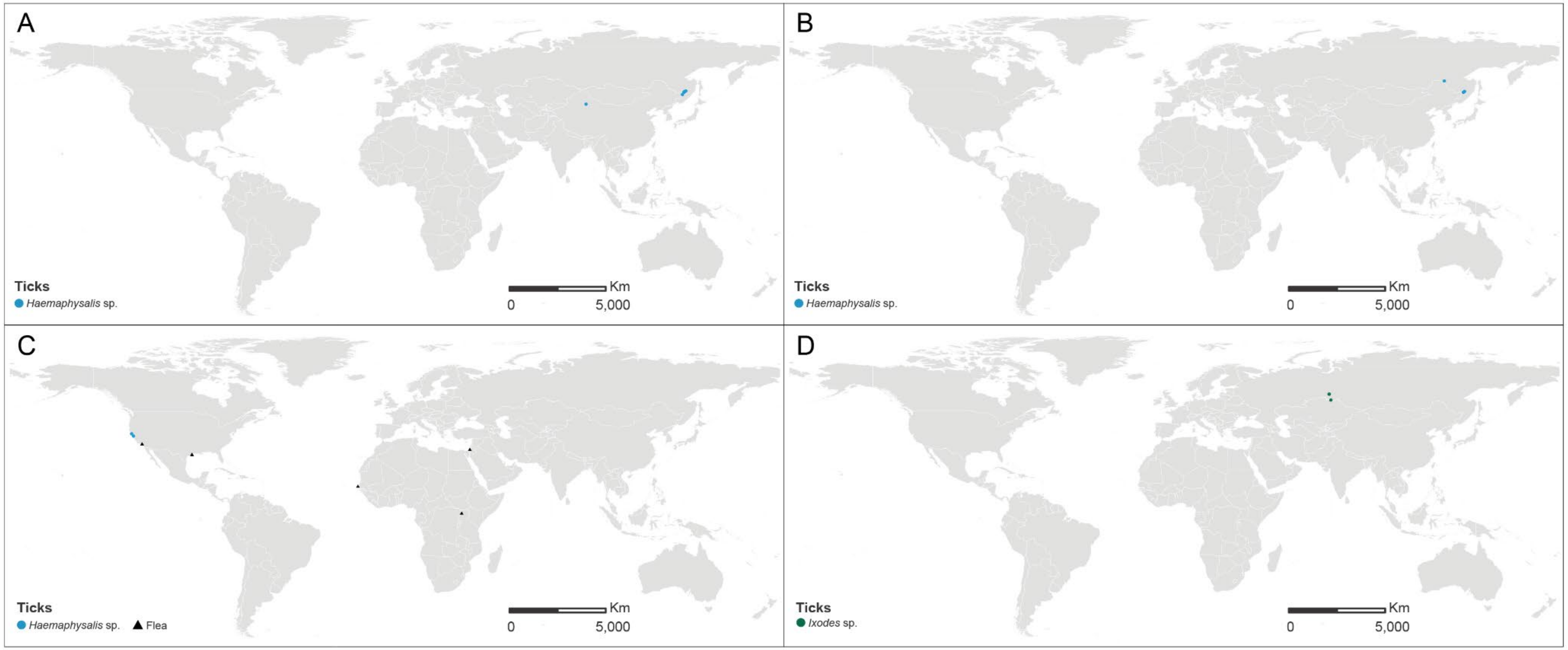

Supplementary figure 48: Clustering of SFGR species based on their ecological features (A) and spatial distributions of the clusters (B-F). The dendrogram in panel A displays the clusters I–V of SFGR species. The features used for clustering are three quantities associated with each predictor in the BRT models. Two of the three quantities were displayed in panel A: (1) relative contributions (colored in ascending order from yellow to red) and (2) position of the median value of the predictor among occurrence locations of the given SFGR species in reference to the quartiles of this predictor among all locations (1–4 indicate 1st-4th quartiles). Panels B–F indicate the spatial distribution of the five clusters (clusters I–V).

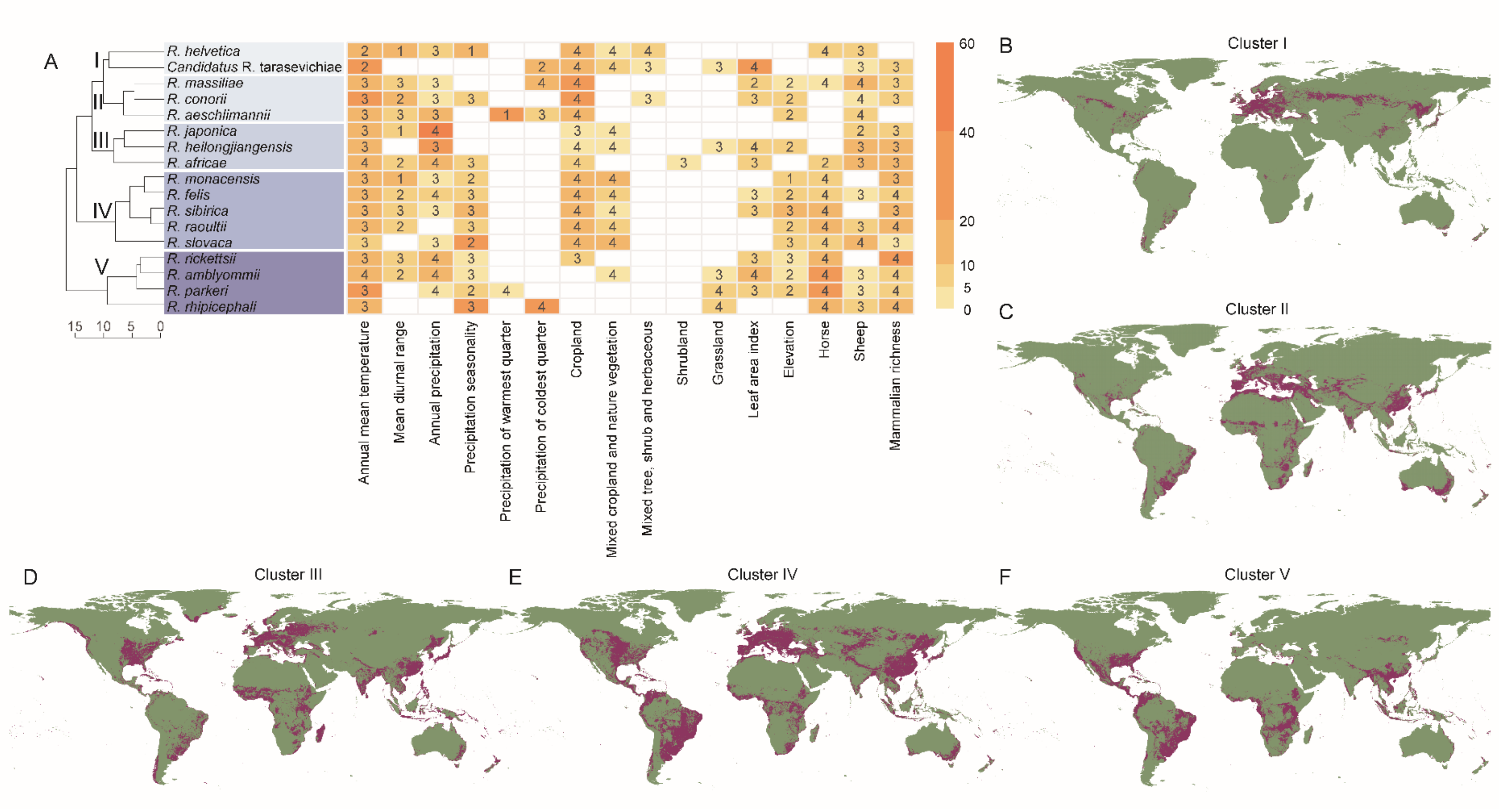

Supplement: 1 [file NIHMS1880530-supplement-1.pdf]
